# An organisational change intervention for increasing the delivery of smoking cessation support in addiction treatment centres: study protocol for a randomized controlled trial

**DOI:** 10.1186/s13063-016-1401-6

**Published:** 2016-06-14

**Authors:** Billie Bonevski, Ashleigh Guillaumier, Anthony Shakeshaft, Michael Farrell, Flora Tzelepis, Scott Walsberger, Catherine D’Este, Chris Paul, Adrian Dunlop, Andrew Searles, Peter Kelly, Rae Fry, Robert Stirling, Carrie Fowlie, Eliza Skelton

**Affiliations:** School of Medicine & Public Health, Faculty of Health and Medicine, University of Newcastle, Callaghan, NSW Australia; National Drug and Alcohol Research Centre, University of New South Wales, Sydney, NSW Australia; Tobacco Control Unit, Cancer Council New South Wales, Woolloomooloo, NSW Australia; National Centre for Epidemiology & Population Health, Australian National University, Canberra, ACT Australia; Drug and Alcohol Clinical Services, Hunter New England Local Health District, Newcastle, NSW Australia; Hunter Medical Research Institute, New Lambton, NSW Australia; School of Psychology, University of Wollongong, Wollongong, NSW Australia; Network of Alcohol and other Drug Agencies, Sydney, NSW Australia; Alcohol, Tobacco and Other Drug Association ACT, Canberra, ACT Australia

**Keywords:** Smoking, Addiction, Organisational change

## Abstract

**Background:**

The provision of smoking cessation support in Australian drug and alcohol treatment services is sub-optimal. This study examines the cost-effectiveness of an organisational change intervention to reduce smoking amongst clients attending drug and alcohol treatment services.

**Methods/design:**

A cluster-randomised controlled trial will be conducted with drug and alcohol treatment centres as the unit of randomisation. Biochemically verified (carbon monoxide by breath analysis) client 7-day-point prevalence of smoking cessation at 6 weeks will be the primary outcome measure. The study will be conducted in 33 drug and alcohol treatment services in four mainland states and territories of Australia: New South Wales, Australian Capital Territory, Queensland, and South Australia. Eligible services are those with ongoing client contact and that include pharmacotherapy services, withdrawal management services, residential rehabilitation, counselling services, and case management services. Eligible clients are those aged over 16 years who are attending their first of a number of expected visits, are self-reported current smokers, proficient in the English language, and do not have severe untreated mental illness as identified by the service staff. Control services will continue to provide usual care to the clients. Intervention group services will receive an organisational change intervention, including assistance in developing smoke-free policies, nomination of champions, staff training and educational client and service resources, and free nicotine replacement therapy in order to integrate smoking cessation support as part of usual client care.

**Discussion:**

If effective, the organisational change intervention has clear potential for implementation as part of the standard care in drug and alcohol treatment centres.

**Trial registration:**

Australian and New Zealand Clinical Trials Registry, ACTRN12615000204549. Registered on 3 March 2015.

**Electronic supplementary material:**

The online version of this article (doi:10.1186/s13063-016-1401-6) contains supplementary material, which is available to authorized users.

## Background

In Australia, 77–95 % of people entering drug and alcohol treatment smoke tobacco [1, 2]; this prevalence is five times that of the general adult population (12.8 % in 2013) [3]. A similar high prevalence of smoking has been reported in US [4] and UK addiction treatment facilities [5, 6]. People with substance use disorders report heavier nicotine dependence and smoke more cigarettes per day than the general population [2, 7, 8]. As a result, people seeking treatment for substance use and substance dependence experience a greater tobacco-related burden of illness [9].

Studies show that clients treated for substance use and dependence are very interested in quitting smoking and can be successful in quitting [2, 10]. Large-scale trials among alcohol-dependent patients show long-term smoking cessation of 10–15 % among those receiving counselling and pharmacotherapy [11–13]. Trials of patients on methadone maintenance programs also show cessation during treatment ranging from 9 % to 33 %, although with a high prevalence of relapse [14–16]. A review of 24 studies showed that smoking cessation intervention enhanced other drug treatment goals as well [17]. A meta-analysis comparing the safety and efficacy of quit interventions during and after addiction treatment found short-term smoking cessation was comparable for participants during treatment and those in sustained remission from substance use disorders [18]. Longer-term treatment benefits in the likelihood of abstinence from alcohol and drugs were found when smoking was addressed during, rather than after, treatment [18]. These results suggest addressing smoking with drug and alcohol clients does not impair treatment and can improve other treatment outcomes.

Tobacco treatment guidelines in Australia [19] and other countries [20, 21] recommend smokers with substance dependence be offered medication and counselling to assist quitting. Evidence-based approaches include assessing smoking status, advising smokers to quit, providing counselling/pharmacotherapy, considering treating substance abuse problems with medications that target these problems but may also help with smoking cessation (e.g., naltrexone with alcoholism), and follow up on quit attempts.

Despite the benefits, studies have found that drug and alcohol treatment centres do not routinely offer clients support with smoking cessation [22, 23]. The decision on whether they address client smoking is left to individual staff members within these centres [23–26]. Factors within treatment settings that reduce the probability of addressing client smoking include a lack of smoke-free policies, staff smoking, a smoking-permissive culture [24], and common beliefs that service users do not want to quit or that quitting will negatively impact the treatment [27].

The use of organisational change approaches to smoking cessation in drug and alcohol treatment centres is novel, and evaluations of its effectiveness in those settings remain preliminary. Organisational changes for smoking cessation involve (1) establishing systems for identifying and recording smoking status; (2) providing education, resources, and feedback to build the capacity of staff to support quit attempts; (3) dedicating staff to provide quit treatment; (4) implementing and promoting organisational policies to provide quit services; and (5) including effective smoking cessation support (both counselling and pharmacotherapy) as part of usual care [28]. Organisational change models also encourage the ‘denormalisation’ of smoking within the treatment environment. Small pilot studies suggest an organisational change approach can successfully integrate smoking cessation support into routine care provided by drug and alcohol clinics [29–32]. For example, Guydish et al. [29] report that 6 months following the introduction of the ‘Addressing Tobacco Through Organizational Change’ (ATTOC) model in three large treatment centres in the US, staff attitudes towards smoking cessation treatment, distribution of nicotine replacement therapy (NRT), and staff provision of tobacco treatments became significantly more positive. ATTOC included staff training in the core component of organisational change, policy development, leadership support, and access to NRT. The next iteration of organisational change research for smoking cessation needs to rigorously evaluate patient outcomes and costs. The protocol has been written following the SPIRIT advice (see Additional file 1).

### Study aims

The primary aim of the Tackling Nicotine Together (TNT) trial is to determine the effectiveness of an organisational change intervention in increasing smoking cessation (7-day-point prevalence of abstinence) amongst clients attending drug and alcohol treatment services. Secondary aims of the trial are to examine the cost-effectiveness of the organisational change intervention compared to usual care and to examine the effectiveness of an organisational change intervention in increasing prolonged smoking abstinence, quit attempts, and the use of cessation aids as well as reducing nicotine dependence amongst clients attending drug and alcohol treatment services. Changes in delivery of smoking cessation care to clients will also be measured.

### Hypotheses

1. Compared to clients in treatment centres allocated to minimal ethical care (control group), clients attending intervention services will have: (1) 9.5 % higher smoking cessation rate at the 6-week follow-up as measured by biochemically verified 7-day-point prevalence of abstinence (primary outcome measure) and (2) 8.5 % higher prolonged abstinence at the 6-month follow-up (secondary outcome measure).
2. The organisational change intervention will be more cost-effective than usual care at 6-month follow-up.

## Methods/design

**Fig. 1:**
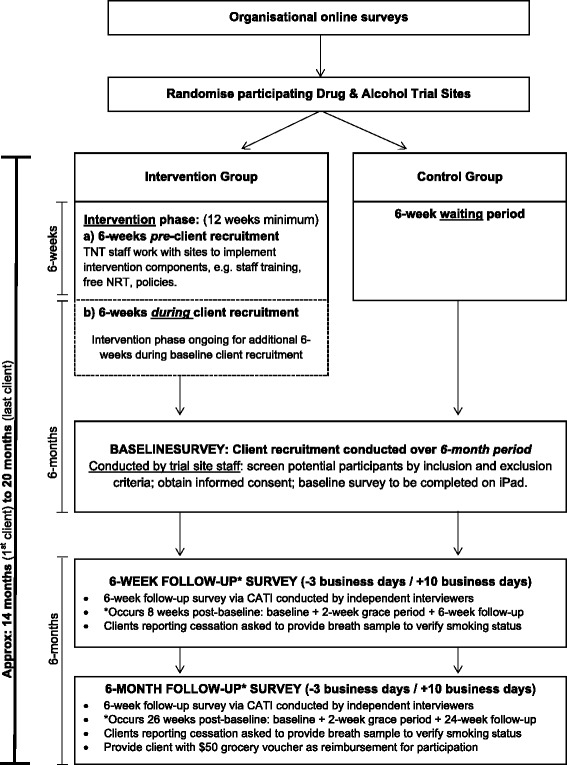
Schematic of the Tackling Nicotine Together (TNT) trial study design. CATI, computer-assisted telephone interview

A cluster-randomised trial will be conducted with drug and alcohol treatment services as the unit of randomisation. The primary outcome measure is biochemically verified client 7-day-point prevalence of abstinence at 6 weeks. As full concealment of allocation is difficult in behavioural cluster trials, this study will use partial blinding, whereby randomisation of the clusters will be performed by a biostatistician not involved with the study and who will be blind to the identity of the treatment centres. Similarly, follow-up assessments will be conducted by interviewers blind to the experimental condition. The flow chart of the TNT trial is displayed in Fig. 1. Ethical approval for this study was obtained from the Hunter New England Human Research Ethics Committee (HREC), Australian Capital Territory Health HREC, South Australia Health HREC, and University of Newcastle HREC.

### Setting

The study will be conducted in 33 governmental and non-governmental drug and alcohol treatment centres in four mainland states or territories of Australia: New South Wales (NSW), Australian Capital Territory (ACT), Queensland (QLD), and South Australia (SA).

### Recruitment and randomisation of treatment centres

#### Recruitment

Eligible drug and alcohol services were those providing services involving face-to-face client sessions to a large number of clients (minimum of 50 per year) in NSW, Queensland, ACT and SA. Recruitment was conducted through key contacts (such as Directors of Health Services or peak non-governmental organisations) who assisted in recruitment. Treatment services were invited to participate by writing and followed up by telephone and in person.

#### Randomisation

Randomisation was undertaken by an independent statistician who used a combination of stratified and restricted randomisation in Stata version 13 to ensure equitable distribution across the intervention groups of stratification factors including the state/territory (NSW, Queensland, ACT, and South Australia), service type (governmental or non-governmental organisation), and smoking policy (partial or total smoking ban). Stratification based on these factors produced ten non-zero strata. Five strata included an even number of sites (24 in total), with these sites randomised within strata to either the intervention or the usual care group. The five remaining strata with an uneven number of sites were combined to form two ‘meta’ strata, based on the state (either ACT or QLD, each with *n* = 4 services) and which were also homogeneous for the type of smoking policy, and one single site strata. The PERCOM module was then used to generate all the possible combinations of treatment allocations within these two meta-strata and restricted the set of all 36 combinations of treatment allocations within strata 1 and strata 2 to be those with balance of service type between treatment arms. One of these 18 balanced allocations was then randomly selected, and finally, the remaining single site was randomly allocated using simple randomisation to either intervention or usual care. This produced a final allocation sequence that, at most, had an imbalance of one site for each stratification factor—the best that could be achieved with an uneven number of sites. All random sampling and treatment allocation was achieved by generation of random uniform values.

### Participants

Treatment service clients eligible for inclusion in the trial will be those aged over 16 years who are attending their first of a number of visits (to allow for repeated exposure to the intervention), who are self-reported current smokers, sufficiently proficient in English, and do not have severe untreated mental illness as identified by treatment staff in order to give informed consent. Informed consent will be obtained from all participants.

### Client recruitment

A staff member at each treatment centre will be trained to conduct client recruitment. Baseline client recruitment will occur over a minimum 6-month period. Eligible clients will be provided with written information about the study and, if interested in participating, asked to complete the consent form and a baseline touchscreen survey. Eligible clients will be informed that the study aims to expand the services provided by the treatment centre, it involves completing a survey about their smoking, and their carer or case-worker may discuss their smoking with them.

### Data collection

Participants will be asked to complete a baseline survey via a touchscreen tablet at the time of recruitment and an additional two surveys over the telephone after 6 weeks and 6 months. At the 6-week and 6-month follow-up assessments, allowing for a 2-week grace period post-baseline, participants who report 7-day-point prevalence of abstinence will also be asked to return to the service to provide a breath sample to measure carbon monoxide (CO).

### Retention strategies

To maximise retention of the participating clients at the 6-week and 6-month follow-up, this study will employ a number of evidence-based strategies [33, 34]. Follow-up dates and times will be set during recruitment, and participants will be provided with a TNT ‘business card’ featuring all study contact details and the 6-week and 6-month follow-up times. Detailed contact information will be obtained from clients agreeing to participate, including their current home address, phone numbers, and email addresses. These contact details will also be collected for a nominated family member or friend. Ongoing contact with the participant via regular text messages, letters, or emails will be used to remind participants to keep their contact details up-to-date with the research team and to remind them when their follow-up survey is coming up. Finally, clients will be offered monetary reimbursement (AUD$50 grocery voucher) when all assessments are completed at the 6-month follow-up for costs incurred (including travel costs and child care).

### Intervention and comparator

#### Control group

Treatment services in the control group will provide usual care.

#### Intervention group

Intervention services will participate in an ‘organisational change’ intervention which aims to establish or improve routine screening, assessment, and delivery of smoking cessation care and treatment to clients. Organisational changes integrate identifying smokers and offering cessation treatment into routine care. The main goal of the intervention is to increase the level of evidence-based brief smoking cessation advice, education, and nicotine replacement therapy offered to clients as part of their usual treatment.

The conceptual framework for the intervention is based on organisational change literature that is tailored to smoking cessation—Fiore et al.’s System Changes approach [20, 28] and Ziedonis’ ATTOC model [32]. The organisational change strategies that will be used to help build the treatment services capacity to deliver the smoking cessation support to clients are outlined below.

1. ***Engaging organisational support***. High-level support for change within the organisation is crucial for implementing change across an entire site [35]. Advocacy, staff meetings, and communications (e.g. newsletters, noticeboards, and email) will be used to sequentially engage all levels of staff and address staff and systems barriers.
2. ***Identifying and supporting a smoking cessation****‘****champion****’*. Having a key staff member who takes the lead role in ensuring smoking cessation treatment is provided to clients can improve treatment delivery and compliance [28, 32]. During training, one staff member in each treatment centre will be nominated by the group to take a lead role. That staff member will be assisted by other support staff to deliver brief advice to clients who smoke, oversee client survey recruitment, and follow up client progress.
3. ***Promoting centre policies that support and provide tobacco dependence services***. Ensuring treatment centres are smoke-free environments will address the barrier of smoking-permissive social norms [36]. On a site-specific basis, project staff will assist with implementing smoke-free policies, smoke-free signage, support for staff to quit, and changes to processes to create a cessation-supportive environment. Services that have policies in place will be assisted in developing programs to maximise enforcement. Total smoke-free policies will be the goal.
4. ***Implementing a system of identifying smokers***. The goal of this strategy is to ensure that all patients are asked about tobacco use as part of every clinical encounter. Each TNT site will be asked to develop a way for assessing and recording client smoking status using their electronic or paper-based record system. Such prompts have been shown to increase the rate that clinicians intervene with smokers [37].
5. ***Providing education and resources****(****including staff training****)*. Training is necessary to ensure that staff has the skills and information to assist their clients in making a quit attempt [28, 32]. Staff will receive training on smoking cessation techniques, focusing on the 5As (ask, assess, advise, assist, and arrange follow-up) and appropriate NRT provision in the form of a 1-day face-to-face workshop with a credited smoking cessation trainer. It is designed to encourage and support smoking cessation practices as part of routine care. The Stages of Change [10] theoretical model is used to assess a smoker’s motivation to quit and tailor support.
6. ***Providing case****-****worker and client feedback***. Computerised feedback has been found to improve the delivery of preventive services in general practice [38]. For the service, newsletters and brief update reports will be used to motivate staff and keep the service engaged with the intervention. In addition, CO monitors will be used to provide motivational feedback to clients regarding their progress during quit attempts.
7. ***Providing evidence****-****based tobacco dependence treatments****(****including NRT****)*. Tobacco dependence treatment is both clinically effective and cost-effective [36]. Providing tobacco dependence treatment within the treatment centre setting reduces the cost barriers to cessation [39]. Existing resources such as QUITline® referrals will be used where appropriate. Treatment centres will be supplied with NRT free of cost during client treatment. This intervention will provide a flexible range of NRT options and extends the limited NRT subsidies currently available through the Australian Pharmaceutical Benefits Scheme for prescription nicotine patches only.
8. ***Planning for maintenance and follow****-****up***. Hospital-based research has found that a smoking treatment summary in the discharge plan aids cessation maintenance [40]. The goal of this strategy is to ensure that clients have a plan in place to maintain cessation or to follow-up on quit interest and intentions on discharge from the service. This may include sending smoking treatment summaries to the client’s primary healthcare provider or linking the client with telephone follow-up services such as Quitline.

Each intervention site will communicate regularly with project staff to ensure that these intervention components are implemented over a 12-week period (minimum). A printed intervention manual (full version – Additional file 2; brief version – Additional file 3) will be provided to each intervention site.

### Measurements

#### Primary outcome measure

The primary outcome measure is client smoking cessation at 6-week follow-up using biochemically verified 7-day-point prevalence. The 7-day-point prevalence of abstinence will be assessed using standard items to determine the proportion of participants who have not smoked any tobacco in the preceding 7 days: ‘Have you smoked a cigarette, even a puff in the last 7 days?’ [41]. Self-reported point-prevalence abstinence at 6 weeks will be verified using measures of CO in expired air.

#### Secondary outcomes

Prolonged abstinence will be assessed using the Russell Standard criteria [42]. The quit date will be determined at the 6-week follow-up and confirmed at the 6-month follow-up, while taking into account a 2-week grace period. Additional secondary outcomes to be collected at client 6-week and 6-month follow-ups include self-reported 7-day-point prevalence of abstinence, nicotine dependence (two-item heaviness of smoking index) [43], number of cigarettes smoked, self-reported quit attempts, and self-reported use of cessation aids.

#### Baseline characteristics

A number of client sociodemographic, smoking, and clinical characteristics will be obtained, including age, gender, marital status, Aboriginal or Torres Strait Islander status, housing status, income and source of income, education, postcode, smoking profile, and other dependencies (alcohol and other drug use).

#### Cost-effectiveness

A cost-effectiveness analysis will be undertaken from the perspective of healthcare providers and patients and will compare the cost and effect from the organisational change intervention to usual care. Costs will be stratified according to whether they fall to the health provider or patient. Health provider costs will include the cost of materials used for education and promoting smoking cessation (but not the research and development cost for preparing the materials), clinical time, case worker time, and the cost of resources committed to cessation treatments. Patient out-of-pocket costs will also be captured. These costs will be compared against the study’s primary outcome measure: 7-day-point prevalence for smoking abstinence at 6-week follow-up. The analysis will compare relative costs and outcomes in the intervention and control centres and report the incremental cost-effectiveness ratio (ICER). A sensitivity analysis will be conducted using variables with uncertain values. The results will be considered in context of strength of evidence, capacity of the intervention to reduce inequity, feasibility, sustainability, and potential for other consequences.

#### Organisational system and process measures

A number of service variables will be measured to examine the relationship between the organisational change intervention and different organisational structures. Structural variables, which will be collected from the head of each treatment service prior to randomisation using an online survey, will include the number of new patients each year, number of full-time and part-time staff, type of treatment provision (e.g. opiate, alcohol or combined) and governmental or non-governmental organisation. At 6-week follow-up, the clients will be asked to indicate which components of the smoking cessation intervention they were offered and/or received.

### Sample size

Within each participating cluster, recruitment will occur over a continuous 6-month period, with the aim of recruiting 35 eligible clients per week (approaching 60 patients with 60 % consent [44, 45]) and giving a sample size at baseline of approximately 450 per group. Although strategies will be employed to reduce attrition and maximise retention, based on previous research [46, 47] 40 % are expected to be lost to follow-up at 6 weeks, thereby providing a sample of 270 participants per experimental arm (an average of 16–17 per treatment centre). Assuming a 5 % significance level, 80 % power, 5 % smoking cessation in the control group for 7-day-point prevalence, a design effect of 1.4 due to correlation of observations within treatment services (an intra-class correlation coefficient of approximately 0.025), and a 10 % allowance for unequal sized clusters, this sample will allow detection of a 9 % difference in the 7-day-point prevalence of abstinence between groups at 6 weeks. For the secondary outcomes, the study will have 80 % power, with a 5 % significance level to detect difference between groups in the 6 week self-reported 7-day-point prevalence of abstinence, use of cessation aids of approximately 9 %, and approximately one quarter of a standard deviation in nicotine dependence and self-reported number of quit attempts. We anticipate a 6-month attrition of 50 %, with detectable differences of 10–11 % for binary outcomes and one third of a standard deviation for continuous outcomes.

### Data analysis

Data will be stored in password-protected files with group code concealed to those who conduct the analysis. A full statistical analysis plan will be developed a priori. Baseline characteristics will be presented for the intervention and control groups. Logistic regression will be used to compare the primary outcome and secondary binary outcomes, linear regression will be used to compare the heaviness of smoking index, and negative binomial regression will be used to compare the number of quit attempts and number of cigarettes smoked between the two groups at each of the two follow-up times. Mixed models will be used to adjust for correlation of outcomes within treatment centres, and models will also include stratification factors (state/territory, service type, smoking policy). Main analyses will involve complete cases, with sensitivity analyses conducted (1) using multiple imputation; and (2) treating missing outcomes as smokers.

The final report will follow the CONSORT 2010 guidelines as well as its extension to cluster trials.

## Discussion

This will be the first methodologically rigorous trial to investigate the efficacy of an organisational change smoking cessation intervention within the drug and alcohol treatment setting. The main expected outcomes are improved health and a cost-effective sustainable organisational change intervention. Most smoking cessation interventions target the individual; this intervention targets the treatment environment and system to produce quality improvement in healthcare provision. Organisational change interventions hold great potential as cost-effective, sustainable strategies. A limitation of individual-targeted approaches is limited reach. Changes to systems reach larger numbers of people and changes are maintained long-term. They also address the environmental context in which the behaviour occurs. Given the study is a multi-state trial, the generalisability of the study outcomes to the treatment centres across Australia is increased. The intervention is also able to be modified for other health behaviour or clinical outcomes of interest (e.g. obesity, alcohol use).

The study is translational in nature with direct implications for health service provision in drug and alcohol treatment centres, including publicly run and non-governmentally run services. If cost-effective, the organisational change intervention has potential for building the capacity of addiction treatment centres to address their clients smoking in the longer term, as well as the potential transferability to other settings, reaching disadvantaged smokers in prisons and mental health settings and through social services. The intervention addresses existing inequity by targeting smoking cessation amongst people in drug and alcohol treatment centres – a cohort five times more likely than the general population to smoke tobacco.

## Trial status

The trial is ongoing.

## Abbreviations

ACT, Australian Capital Territory; ATTOC, Addressing Tobacco through Organizational Change; CO, carbon monoxide; HREC, Human Research Ethics Committee; NRT, nicotine replacement therapy; NSW, New South Wales; SA, South Australia; TNT, Tackling Nicotine Together.

## Additional files

Additional file 1: Spirit checklist. A table specifying where any SPIRT item has been addressed in the protocol manuscript. (PDF 129 kb) Additional file 2: TNT Intervention Manual - FULL. An organisational change intervention manual for smoking cessation in drug and alcohol treatment centres. A comprehensive manual detailing the TNT intervention strategies with reference to the background literature supporting each of the intervention components and guiding theoretical frameworks. (PDF 3047 kb) Additional file 3: TNT Intervention Manual - BRIEF. TNT Project Working Manual. A brief working document containing 1–2 page user-friendly summaries of each of the eight components of the TNT intervention with links to resources developed and/or provided as part of the trial. (PDF 2612 kb)
