# Supplementary material for: An organisational change intervention for increasing the delivery of smoking cessation support in addiction treatment centres: study protocol for a randomized controlled trial
Source: Trials. 2016 Jun 14;17:290. doi: 10.1186/s13063-016-1401-6 (PMC4907075; doi:10.1186/s13063-016-1401-6)
Supplement: Additional file 2: — TNT Intervention Manual - FULL. An organisational change intervention manual for smoking cessation in drug and alcohol treatment centres. A comprehensive manual detailing the TNT intervention strategies with reference to the background literature supporting each of the intervention components and guiding theoretical frameworks. (PDF 3047 kb) [file 13063_2016_1401_MOESM2_ESM.pdf]

An Organisational Change Intervention Manual for Smoking Cessation in Drug and Alcohol Treatment Centres

This is a collaborative project between

Which is funded by

NHMRC and the Cancer Council NSW

TNT STUDY MANAGEMENT & ADVISORY TEAM

**Associate Professor Billie Bonevski – Chief Investigator**<br>School of Medicine & Public Health<br>University of Newcastle<br>billie.bonevksi@newcastle.edu.au	
**Prof. Anthony Shakeshaft**<br>National Drug & Alcohol Research Centre<br>University of New South Wales<br>a.shakeshaft@unsw.edu.au	**Prof. Michael Farrell**<br>National Drug & Alcohol Research Centre<br>University of New South Wales<br>michael.farrell@unsw.edu.au
**Dr. Flora Tzelepis**<br>School of Medicine & Public Health<br>University of Newcastle<br>flora.tzelepis@newcastle.edu.au	**Mr. Scott Walsberger**<br>Tobacco Control Unit<br>Cancer Council NSW<br>scottw@nswcc.org.au
**Prof. Catherine d'Este**<br>National Centre for Epidemiology and Population Health (NCEPH)<br>The Australian National University<br>catherine.deste@anu.edu.au	**A/Prof. Christine Paul**<br>School of Medicine & Public Health<br>University of Newcastle<br>chris.paul@newcastle.edu.au
**Dr. Jamie Bryant**<br>School of Medicine & Public Health<br>University of Newcastle<br>jamie.bryant@newcastle.edu.au	**Dr. Peter Kelly**<br>School of Psychology<br>University of Wollongong<br>pkelly@uow.edu.au
**A/Prof. Andrew Searles**<br>Hunter Medical Research Institute &<br>University of Newcastle<br>andrew.searles@newcastle.edu.au	**A/Prof. Adrian Dunlop**<br>Drug and Alcohol Services<br>Hunter New England Lower Health District<br>adrian.dunlop@hnehealth.nsw.gov.au
**Ms Rae Fry**<br>Tobacco Control Unit<br>Cancer Council NSW<br>raef@nswcc.org.au	**A/Prof. Judith Prochaska**<br>Stanford Prevention Research Centre<br>Stanford University<br>jprochaska@stanford.edu
**Ashleigh Guillaumier – Project Manager**<br>School of Medicine & Public Health<br>University of Newcastle<br>ashleigh.guillaumier@newcastle.edu.au	**Eliza Skelton – PhD Student**<br>School of Medicine & Public Health<br>University of Newcastle<br>eliza.skelton@newcastle.edu.au

Table of Contents

Objectives of the Manual 5

Background to the TNT Study 6

Components of the TNT intervention 10

1. Engage Organisational Support 12
2. Identify and Support a Champion 15
3. Promote Centre Policies that Support and Provide Tobacco Dependence Services 19
4. Implement a System of Identifying Smokers 23
5. Provide Education and Resources 26
6. Provide Case-Worker and Client Feedback 29
7. Include Evidence-Based Tobacco Dependence Treatments 32
8. Maintenance and Follow-Up 39

References 41

TNT Project Contacts 45

TNT Resource Kit 46

Resource 1.1: CCNSW Tackling Tobacco Pamphlet – "Incorporating smoking cessation into drug and alcohol treatment – Information for staff" 47

Resource 1.2: Agenda template for TNT briefing staff meeting 48

Resource 1.3: Newsletter article templates for TNT 49

Resource 1.4: Reminder email or flyer prompts for changes to tobacco-related polices & practices 50

Resource 2.1: Support Champion "Identifier" Poster 51

Resource 3.1: CCNSW "Tackling Tobacco Policy Toolkit" 52

Resource 4.1: Desktop reminder to assess smoking status 53

Resource 5.1: CCNSW Tackling Tobacco pamphlet – "Not ready to give up" 54

Resource 5.2: CCNSW Tackling Tobacco pamphlet – "Thinking about giving up" 55

Resource 5.3: CCNSW Tackling Tobacco pamphlet – "Ready to give up" 56

Resource 5.4: CCNSW Tackling Tobacco pamphlet – "Staying a non-smoker" 57

Resource 5.5: Quit kits and ordering information 58

Resource 6.1: Quitline Fax Referral Form 59

Resource 6.3: CO monitor protocol 61

Resource 7.1: 5A's Poster 62

Resource 7.2: Motivational Interviewing Strategies 63

Resource 7.3: The 5R's – for the smoker unwilling to quit 64

Resource 7.4: Stages of Change approach – decision branching tool 65

Resource 7.5: Nicotine Withdrawal – What is it? 66

Resource 7.6: Nicotine Replacement Therapy – General guide 67

Resource 7.7: NRT Protocol 70
Resource 7.8: What if the NRT is not working? 71
Resource 7.9: Relapse and Coping Strategies 72
Resource 7.10: NRT log 73
Resource 8.1: Letter to GP 74

Objectives of the Manual

The aim of this manual is to describe the evidence and theoretical basis of an organisational change intervention for smoking cessation in drug and alcohol treatment centres as well as the practical application of implementing these changes. The methods for the development of the manual include:

1. Literature reviews on organisational change and the intervention components in order to develop a well-rounded approach to ensure maximum implementation and long-term adoption of strategies.
2. Reference to the COM-B model of behaviour change[(1)] and the Consolidated Framework For Implementation Research (CFIR).[(2)]
3. Formative research including focus groups with clients of drug and alcohol services and interviews with staff.

Background to the TNT Study

Smoking is the main preventable burden of disease in Australia and a significant cause of high healthcare costs.[3, 4] In Australia 77-95% of people entering drug and alcohol treatment smoke tobacco – 5 times that of the general adult population.[5, 6] This population have heavier nicotine dependence and smoke more cigarettes than the general population.[6-8] As a result, they experience greater tobacco related burden of illness[9] including substantial social and financial costs to themselves, their families, and society.

Surveys show despite high smoking rates, clients treated for substance use are very interested in quitting smoking and are successful in doing so.[6, 10] Large scale trials among alcohol dependent patients show long term smoking quit rates.[8, 11, 12] Trials of methadone patients also demonstrated positive within treatment quit rates, although had a high rate of relapse.[13-15] A review of 24 studies showed smoking cessation enhanced other drug treatment goals as well as significantly reduced smoking rates compared to no treatment.[16] A meta-analysis comparing safety and efficacy of quit interventions during and after addiction treatment, found short term smoking cessation rates comparable.[17] Longer-term effects on alcohol abstinence were beneficial if smoking was addressed during rather than after treatment.

Addressing smoking with drug and alcohol clients does not threaten treatment and can improve other treatment outcomes. Tobacco treatment guidelines in Australia[17] and overseas[18] recommend smokers with chemical dependence be offered medication and counselling to assist quitting. Evidence based approaches include: identify smoking status at every visit; advise smokers to quit; assess readiness to quit; provide counselling / pharmacotherapy; consider substance abuse medications that may also help with smoking cessation; follow up on quit attempts.

Drug and alcohol centres rarely offer help to quit smoking and the decision to address client smoking or offer interventions is left to individual staff members.[19-22] Environmental or systems-based factors in treatment settings that reduce smoking being addressed include: lack of smoke-free policies; staff smoking; smoking permissive culture,[22] along with common beliefs that substance abusers don't want to quit or quitting will impact negatively on other treatment.[23]

A US audit of drug and alcohol facilities found NO systems for consistent, evidence based tobacco treatment or quality improvement.[24] System level changes for smoking involve: a) identifying and recording smoking status; b) promoting staff intervention to support quit attempts; c) dedicating staff to provide quit treatment; and d) promoting organisational policies supporting and providing quit services.[25] System change models also include the 'denormalisation of smoking'. Feasibility studies show a system change approach can successfully integrate tobacco treatment into routine care provided by drug and alcohol clinics.[26-28]

TACKLING NICOTINE TOGETHER: STUDY OVERVIEW

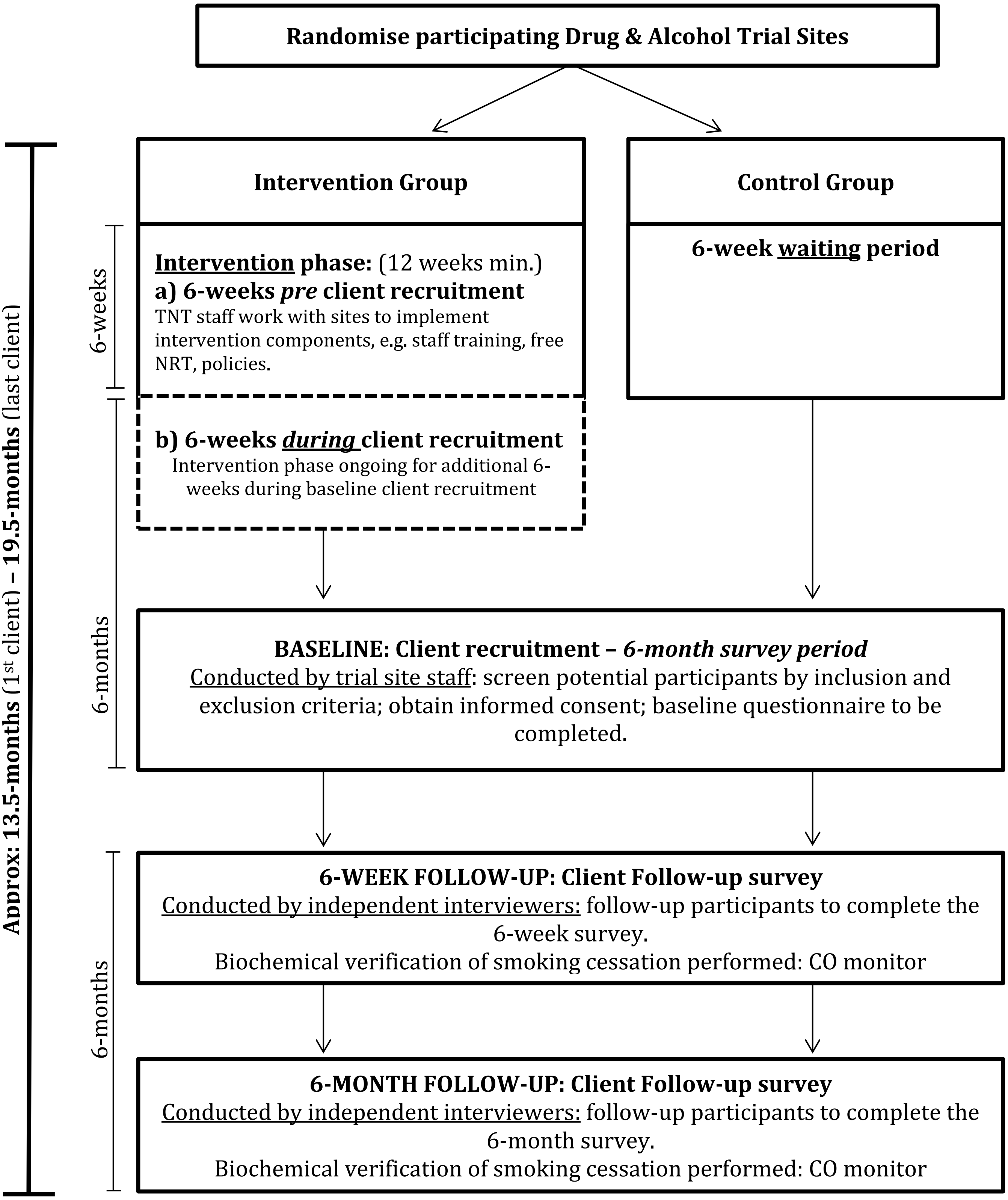

The following information has been adapted from "Literature Review of Organisational Change to Address Smoking in Community Mental Health Organisations" commissioned by Cancer Council NSW and the Mental Health Coordinating Council as part of the CCNSW Tackling Tobacco program.

What is Organisational Change?

Organisational change is a largely subjective concept that can be defined by different sectors and groups of individuals depending on the context, setting and purpose.

In the context of literature in the health care setting, it can be understood as:

1. a process that occurs over time as opposed to a single one off event[29]
2. the coordination of partnerships, training and culture change to implement and sustain evidence based practice[30]
3. a staged process of change with multiple layers[31]
4. a framework for managing the introduction of a new idea or practice[28, 31]

All of the above descriptions of organisational change are applicable to changing practices in the drug and alcohol treatment setting and the above descriptions have been consolidated to form the follow definition:

The planned process for managing change at all levels of an organisation for the introduction of a new practice that is sustainable and long-standing.

Why use an Organisational Change intervention?

Interventions targeting only clinicians and not the organization in which they practice are found to be limited in their effectiveness as well as reach and fail to integrate core smoking cessation components when treating tobacco use and dependence.[32] There is also a particularly pervasive culture of acceptance in not treating tobacco dependence in substance abuse treatment as it is not perceived as part of their core treatment which has further created the need for interventions to be intensive in approach, addressing a number of aspects. As such, organizational change interventions represent a useful and logical approach as they employ a multi-faceted design.

Theoretical Frameworks

The Consolidated Framework for Implementation Research (CFIR)(2) will be used. The CFIR encompasses a broad array of implementation theories into a unified and comprehensive framework with consistent terminology and definitions. The framework has the potential to enable researchers to understand what works where and why for the setting that they wish to conduct research within.

As the intervention is targeted at changing the organisation environment to elicit individual behaviour change, the CFIR will be complimented by a practical framework for behaviour change, The Behaviour Change Wheel based on the COM-B model of behaviour change(1) (Figure 2). The COM-B provides an efficient method for choosing appropriate intervention components to target a given behaviour specific to that given context and within the specific population.

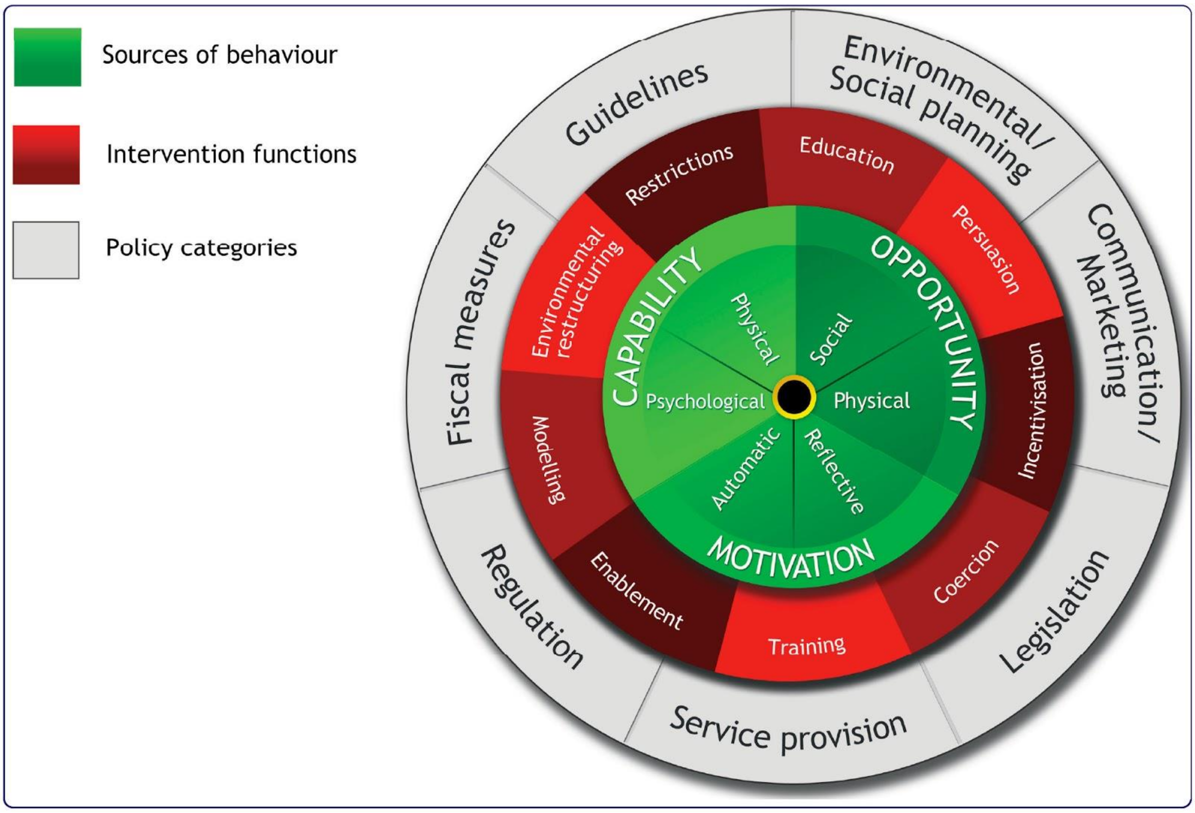

Figure 1. The Behaviour Change Wheel (Michie et al, 2011)

The following sections of the manual will explain in detail each of the TNT intervention strategies.

Components of the TNT intervention

The design and conceptual framework of the TNT intervention is based on the structure, aims and methods of two models of organisational change to address smoking: Fiore et al.'s 'Systems Changes Approach' and Ziedonis et al.'s 'Addressing Tobacco through Organisational Change' (ATTOC) model.

The resulting TNT organisational change intervention is devised of eight core components:

1. Engage Organisational Support
2. Identify and support a champion
3. Promote centre policies that support and provide tobacco dependence services
4. Implement a system of identifying smokers
5. Provide education and resources
6. Provide case-worker and client feedback
7. Include evidence-based tobacco dependence treatments
8. Maintenance and follow-up

These eight organisational change components can be each conceptualised as pieces of a puzzle that when combined are the Tackling Nicotine Together intervention (see figure 2).

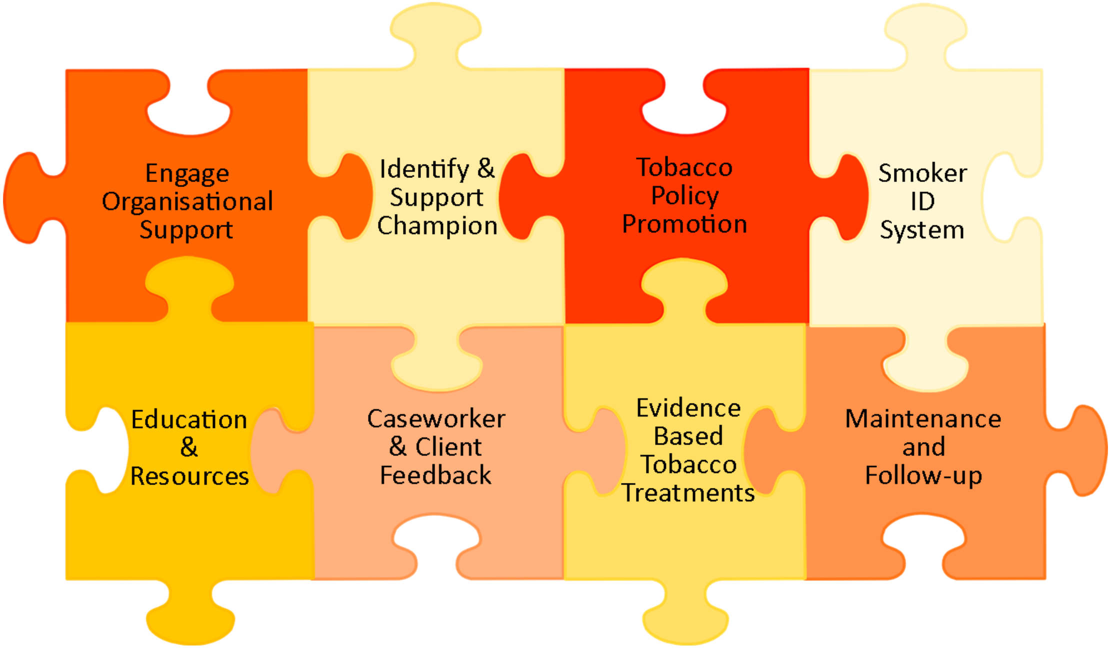

Figure 2. TNT organisational change intervention components

Flexibility of the TNT intervention

The TNT intervention is flexible and can be tailored to the needs of each of the individual drug and alcohol services participating in the study. Each participating service will have different needs relating to tobacco policy and provision of smoking cessation support. The TNT research team will work with each service to build the best combination of intervention components that builds on any existing policies, services and resources.

Below is a depiction of the ways the TNT intervention can be tailored to the individual needs of services participating in the study:

<u>Scenario 1: Service A</u>

**Current practice:**	**Requires assistance with:**	**Tailored TNT intervention**
• Written smoke-free policy<br>• Smoker identification system in place<br>• All staff recently completed smoking cessation training (including maintenance and follow-up strategies)	• Engaging high level organisational support<br>• Identifying and assisting a smoking support champion<br>• Provision of tobacco treatments and resources	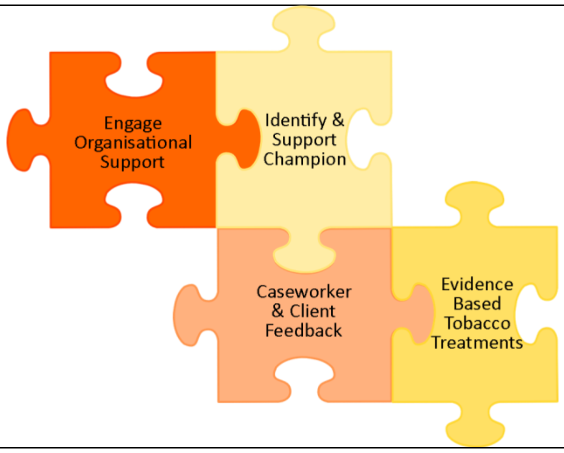

<u>Scenario 2: Service B</u>

**Current practice:**	**Requires assistance with:**	**Tailored TNT intervention**
• High level of organisational support<br>• Smoking support champion identified	• Written smoke-free policy<br>• Staff training<br>• Developing a system to identify smokers<br>• Provision of tobacco treatments and resources	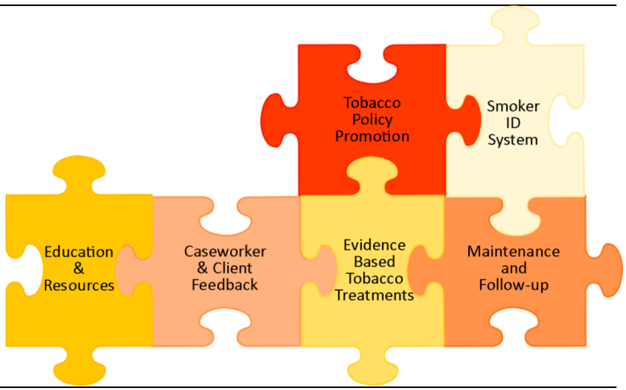

1. Engage Organisational Support

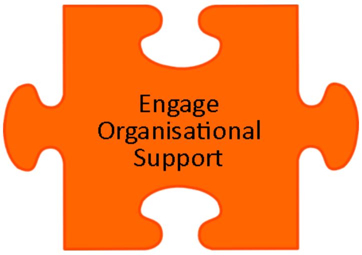

1.1. Goals of this strategy

To engage the support and commitment from management and staff throughout the project period (pre intervention, during implementation and post intervention) in order to increase the likelihood of adoption and maintenance of the Tackling Nicotine Together intervention.

Engaging the support of the organization and its employees is a critical factor for organizational transformation**.** Staff and clients that are highly engaged and supportive of the intervention will create a climate that is ready for change which will facilitate the integration of the intervention into practice and will assist in the maintenance and sustainability of the components. Engaged organisations are also found to be less likely to cease the change effort.

In line with the COM-B theory of behaviour change, seeking the commitment of an organisation's management and senior staff for a systems change intervention reduces the barriers to increasing that service's capability.

1.2. Evidence for this strategy

High level support for change within the organisation is crucial for implementing change across a whole site.(33) Engaging the broad support of management and senior staff is essential for implementing successful change across an organisation. It is important that staff in leadership roles clearly articulate their support for the change and lead by example. Organisational support should occur at multiple levels, fostering an environment of collaboration and engagement with the improvement aims.

Organisational climate or the readiness for change directly affects engagement in change and adoption of new practices.(34) The process of implementing begins with consideration of program needs and resources, structural and functional characteristics and general readiness to embrace innovations.(35) Individuals are more likely to engage in change if the evidence for the intervention comes from individuals working in a similar field or role. A study on mental health practitioners in the community setting found the information source for the change played an important role in their willingness to adopt and comply with the required changes and tasks.

Critical to successful engagement is good planning and project management. A good planning process involves: setting a clear vision for the change, which is aligned with the organisation's vision and mission, documenting the case for change and developing a change plan. An overall strategy by which an organisation can develop a comfort level in dealing with change is to reduce the perception that change is always scary, abrupt and fraught with negative consequences. One way to address this discomfort is to promote the development of change champions within the organisation.

Communication is vital to engage organisational support. Clear, well planned, high impact messages can help staff see the connect between their work and the intervention goals but also enable them to understand how their support will drive to an overall increase in organisational performance which is tied directly to their engagement levels. Poorly communicated changes results in rumours and resistance to change, exaggerating the negative aspects of the change.[36]

1.3. Main steps to implementation of this strategy

A number of strategies can be used to engage and demonstrate organisational support for the TNT intervention. Advocacy, staff meetings and communications (e.g. newsletters, noticeboards, email) will be used to engage all levels of staff and address any staff and/or systems barriers that arise. Initially, strategies will be addressed sequentially, then services will be encouraged to use as many of the strategies listed below as possible to encourage engagement with the smoking cessation program throughout and beyond the intervention period.

The following is an excerpt from the Queensland Government's "*Change Management Best Practices Guide*"[37] and lists the fundamentals of change communication:

- Clearly communicate the change vision and do it early
- Outline the benefits and impacts of the change
- Ensure the organisation's leaders are actively communicated with throughout the change process
- Use multiple channels to communicate the change message
- Provide opportunities for dialogue
- Repeat change messages often
- Monitor and measure the effectiveness of communications

1.3.1. **Advocacy**

Information regarding the need for addressing smoking cessation in the context of drug and alcohol treatment will be provided to management and staff (see Resource 1.1). Building a case for change can involve presenting evidence of the tobacco burden of disease among AOD clients and the improvements that can be achieved in the health and wellbeing of clients when smoking cessation support is offered and provided.

The evidence base for intervening and making changes to current practices needs to be presented to the organisation by an individual who is seen as a credible source of trustworthy and reliable information. The presentation of the intervention can be either verbal or visual and needs to be in favour of adopting the new practices and the importance of smoking cessation care. The use of a credible source to engage organisation support to implement intervention components is related to the behaviour change technique 9.1.

1.3.2. **Staff Meetings**

Initial staff briefing

Prior to the start of the intervention period, a staff meeting should be held to inform all staff in the service of the project and to discuss the best ways to maximise engagement with the intervention program.

Standing meeting agenda item

The smoking cessation intervention program could be added as a standing agenda item for staff meetings to provide an on-going forum for discussion of successes and challenges in implementing the change in practice as part of routine care (see Resource 1.2).

1.3.3. Organisation-wide communications

To promote on-going awareness of the change in policies and practices involved with the TNT intervention, organisations will be encouraged to make use of a number of communication strategies. Methods of message communication may include:

- Articles in existing organisation newsletters (see Resource 1.3)
- Brief reminders about new tobacco policies and practices – these prompts can take the form of emails or flyers (see Resource 1.4)

1.4. Resources for this strategy

RESOURCES FOR THIS STRATEGY	
Resource 1.1	CCNSW Tackling Tobacco pamphlet "Incorporating smoking cessation into drug and alcohol treatment – Information for staff"
Resource 1.2	Agenda template for TNT briefing staff meeting
Resource 1.3	Example newsletter articles
Resource 1.4	Reminder email or flyer prompts

2. Identify and Support a Champion

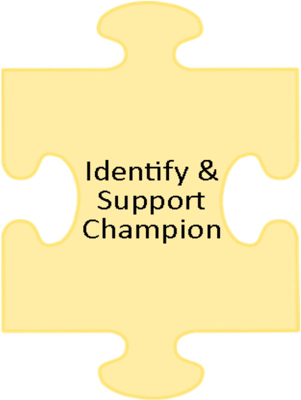

2.1. Goals of this strategy

To identify members of staff from each organization to be appointed the role of support champion who will facilitate the adoption of the organisational change intervention components. The aim is to identify and support staff that will champion the introduction and/or improvement in delivery of smoking cessation care as part of usual care.

2.2. Evidence for this strategy

Having a key staff member who takes the lead role in ensuring smoking cessation treatment is provided to patients can improve treatment delivery and compliance.[26, 28] In line with Michie and colleagues' theory of behaviour change, the COM-B, modelling is used as a motivational tool by engaging an individual's propensity to imitate.

Support champions are instrumental to ensuring intervention adoption. For organisations to successfully adopt innovations the organisational culture (norms, assumptions, values) need to be realigned with the new vision. A key strategy to firstly prepare and later diffuse the required changes is to utilize individuals who have personal and professional influence over their peers to actively encourage the changes. As such these individuals are generally referred to as support champions and their goal is to reorient the current culture as well as drive the implementation and overcome resistance towards the changes.

The Support Champion has defining personal attributes. Support champions must believe in the change, and can effectively communicate the vision while promoting engagement and assist in overcoming staff or client resistance.[38] They need to be seen as credible and supported by higher management within the organization and provided with the required resources to successfully enact changes.[38]

2.3. Main steps to implementation of this strategy

2.3.1. Selecting a Champion

The selection of a support champion will be dependent on the type of organisation. Appointment should occur prior to the beginning of the intervention as the support champions are important in the initial stages of engaging organisational support.

Support champions can be selected through one of two ways:

Formal: careful and thoughtful selection process of well-defined eligibility criteria created by managerial staff that individuals must satisfy

Informal: individuals are seen to naturally fulfil the role in their normal work capacity yet have had no formal appointment

The following criteria may be used as a guide to help each organisation choose their support champion(s):

- Current employee
- Degree in health-related field
- Well-known to staff
- Highly developed communication skills who is tactful and easily builds rapport with others
- Current opinion leader or individual who is regarded as influencing others
- Experienced in training staff
- Ability to lead and supports team dynamics
- Resilient and persistent in approach, a critical thinker who is able to be flexible in averse conditions
- Creative, innovative and a charismatic advocate

The support person will be formally recognised at the organisation as the TNT champion. In order to notify all staff of the individual who has fulfilled the role as the support champion a poster will be placed in the staff room of the organisation. The poster will have the name and picture of the support champion as well as a brief outline of their role and how they can help staff and clients (*see Resource 2.1*).

2.3.2. **Training of a Champion**

It is particularly important for the selected support champion to attend staff training on the provision of smoking cessation support (see *section 5. Provide Education and Resources*). Training sessions will take the format of a one-day workshop conducted by an accredited Tobacco Cessation Trainer with extensive experience in the drug and alcohol field.

Topics relevant to the champion that will be covered during the training are:

- The role of a support champion and their new responsibilities
- How to provide appropriate brief advice to clients about smoking cessation
- Instructions on how to disseminate the intervention information appropriately at their workplace.

Over the course of the research project support champions will meet regularly with the research staff via teleconference to receive ongoing assistance and troubleshoot any concerns they may have. The research team will also facilitate teleconferences for support champions from different organisations to exchange information and provide peer support.

2.3.3. **The Role of a Champion**

Advocate for change

The champion will advocate changes to organisation policies and practices that support the provision of smoking cessation support as part of routine care. The champion will encourage the creation of new ideas from staff members and provide the entry point for change, engaging individuals to make the required changes.

Primary contact for the research team

The champion will be the main point of contact between the research team and the organisation. This relationship will serve as the primary method for dissemination of project materials and key intervention messages. This line of communication will also be open for champions to troubleshoot issues or refer on any concerns relating to the research project.

The support champion will have direct contact with the research staff. The support champion will be able to request any further resources that they feel they need as well as provide feedback on current resources available to them. The individual will also have the direct email address and phone numbers of project research assistants if they wish to contact the research team prior to any scheduled meetings.

Maintain the TNT intervention as a priority

The champion will work to maintain the TNT project as a priority among staff and will lead the project activity at their organisation.

At staff meetings the champion can provide a recap of the intervention and feedback about how their organisation is performing. This information will be provided to the champion by the research team via regular email updates and monthly newsletters. The champion will also remind staff that they are able to approach them to speak to them about any concerns or assistance they require to help implement and adopt the TNT-related policy and practice changes.

The champion will also be responsible for TNT project-related equipment, such as survey data collection devices, paper-based information statements, equipment logs, etc.

Support staff development and troubleshooting

The champion will support other staff members to deliver smoking cessation care as part of routine care and will work to positively implement policy level and environmental changes to the workplace. The champion will provide advice and share information, disseminate knowledge, build relationships, navigate boundaries and facilitate consensus. The champion might achieve this by prompting discussions within the organisation and among staff to identify the barriers preventing them from achieving their goal.

2.4. Resources for this strategy

RESOURCES FOR THIS STRATEGY	
Resource 2.1	Support Champion “Identifier” Poster
TNT Monthly Newsletter	A monthly newsletter will be provided to the support champion to distribute to staff of the organisation. The newsletter will be created by the research team with the assistance of the support champion. The newsletter will contain information concerning the project objectives, current goals, resources available to staff/clients, upcoming training and ways to contact the support champion about issues/ concerns
Touchscreen Tablet (survey data collection tool)	A touchscreen tablet device will be allocated to each organisation. This device will be used to conduct surveys with clients. A safe lock box for the device will be provided as well as instructions on how to secure the device. The support champion will be responsible for the device and must ensure that it will not be at risk of theft.

3. Promote Centre Policies that Support and Provide Tobacco Dependence Services

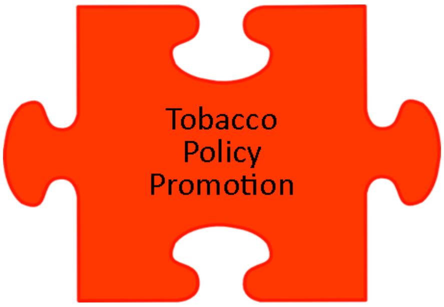

3.1. Goals of this strategy

Ensuring treatment centres are smoke-free environments will address the barrier of smoking-permissive social norms.[39] Project staff will assist with implementing smoke-free policies, smoke-free signage, support for staff to quit and changes to processes to create a cessation-supportive environment. Services that have policies in place will be assisted in developing programs to maximize enforcement.

An important goal of this strategy is to develop organisation-specific tobacco policies, and to ensure that all staff and clients of the organisation are aware of the policies and understand what they include.

3.2. Evidence for this strategy

Substance use treatment centres have been slower than other parts of health services to implement restrictions or smoking bans and provide tobacco dependence services.[40] Despite clinical guidelines and research evidence supporting tobacco free policies and the provision of tobacco dependence treatment they are infrequently implemented in substance use disorder facilities. In the US, estimates are that only around 10-20% of treatment centres have policies that completely prohibit smoking.[41]

Centres need to develop smoke free policies and social norms to promote and assist tobacco users to quit and to prevent initiation of tobacco use. The introduction of centre-based smoke free policies represent an important step is addressing the harm caused by tobacco smoking. Total smoking bans in hospitals and health services seek to protect the health and wellbeing of individuals while reducing the risks to staff and patients from harmful exposure to environmental tobacco smoke[40] and to ensure that staff advice in quitting smoking is not undermined by ensuring a healthy environment.[42] An important barrier to implementing tobacco policies and treatment services is the belief that such policy implementation will result in a decrease in the number of clients that enrol and an increase in clients who leave prematurely.[43] Kotz (1993) found initial patient and staff resistance after going tobacco free however the number of clients enrolled in the program was found to increase.[44] Other studies have found that clients in a stop smoking program remained in

inpatient treatment longer than those that did not[45] and early discharges were not found to increase.[43]

Total Smoking bans are better than partial restrictions**.** Evidence suggests that total smoking bans are more sustainable than partial smoking bans, more effective at reducing staff exposure to environmental tobacco smoke and less likely to result in patient complaints or verbal aggression. Tobacco free grounds support tobacco dependence treatment**.** Quitting tobacco smoking is difficult in a treatment setting that allows tobacco use because of the abundance of triggers in the environment.[46] Reinforcing health behaviours and drug-free environment is critical even if patients smoke after discharge. When tobacco use is permitted in these settings, it may cause ex-smokers to relapse back to smoking or allow others to experience smoking for the first time.[43] The move to tobacco-free grounds is similar to a workplace-smoking ban and such bans are shown to reduce workforce smoking.[47] Individuals who smoked when entering residential treatment were almost five times more likely to quit smoking in treatment due to the enforcement of the smoke free policy. Workplace bans are found to reduce exposure to second-hand smoke whether measured via self-report or biochemically, as well as being associated with improvement in employee respiratory health.[42]

3.3. Main steps to implementation of this strategy

The implementation of this strategy will be largely dependent on the current policies and practices of the organization. Services that do not currently have a smoke-free policy will be encouraged to work through the CCNSW Policy Toolkit (see Resource 3.1) to develop their own policies. Other services may already have comprehensive smoke-free policies in place. All services will be assisted in developing programs to maximize awareness of the service tobacco policy and enforcement strategies.

The research team will assist with the development and/or refinement of smoke-free policies, smoke-free signage, support for staff to quit and changes to processes to create a cessation-supportive environment. All services, including those that have comprehensive policies in place, will be assisted in developing programs to maximize enforcement.

3.3.1 Policy Development

Policy needs to consist of a statement, policy aims, policy components and a plan for implementation.

Policy aims:

- Reduce the harm associated with tobacco use, including exposure to environmental tobacco smoke, for both smokers and non-smokers
- Encourage and support staff and clients to quit smoking
- Provide a clear and consistent message to staff and patients about the hazards of smoking
- Position them as a leader in tobacco management
- Better align the centre’s anti-smoking policy with the government policy for the state

The policy will be achieved through:

- Education and communication
- Cessation support
- Environmental restrictions

Legislation currently bans smoking in a range of public settings, including enclosed area of the workplace, on public transport and in restaurants, bars and clubs. There restrictions are intended to protect people from the harm associated with second-hand smoke

3.3.2 Policy Promotion

The physical environment of the organisation can be restructured in order to be more supportive of the policy changes. Restricting access to areas that were once used for smoking or transforming them into areas that have a different purpose could take place. Restructuring the physical environment such as redesigning the use or purpose is related to the behaviour change technique 12.1.

Adding objects to the environment to help ensure non-smoking behaviour is maintained is an important step. Signs are an effective visual tool to highlight to individuals that the behaviour is inappropriate for the area that they are in. The addition of objects to the environment is related to the behaviour change technique 12.5.

3.3.3 Policy Enforcement

An aversive consequence should be arranged for when the smoking of cigarettes is performed. The punishment for tobacco use should be well known to staff and clients to further deter the potential for individuals to contemplating in the participation of the behaviour. Punishment is related to the behaviour change technique 14.2.

Rewarding non-smoking

Rewards are to be arranged for individuals not smoking in the organisation grounds. If the policy change has been to abolish smoking permitted areas (or hours) and individuals have not been found to be smoking in these areas for an amount of time then this feedback should be provided to clients/ staff and a reward decided upon and given for everyone to enjoy. A situation specific reward is related to the behaviour change technique 14.6

Pre-warning of the consequences of smoking

Both patients and staff are to be informed that there will be future punishment or the removal of a reward (group activity) as a consequence of being found smoking tobacco on the organisation’s grounds. Individuals are to be informed that by continuing to smoke that there will be penalties such as a written warning and consequently this could lead to a loss of job or being asked to leave the centre early. By providing a warning and the consequences of performing the behaviour the future punishment is the behaviour change technique 10.11.

Punishment for not abiding by the organisation's policy
The severity of the punishment for performing the prohibited behaviour of smoking at the organisation will depend on what the centre staff and management believe is appropriate. The appropriate punishment can be decided upon after the behaviour has been performed. It can be openly stated that there will be consequences but the exact consequence does not need to be publicly stated or known.

3.4. Resources for this strategy

RESOURCES FOR THIS STRATEGY	
Resource 3.1	CCNSW "Tackling Tobacco Policy Toolkit"

4. Implement a System of Identifying Smokers

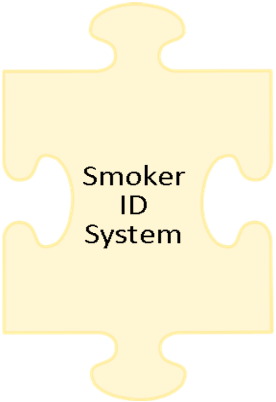

4.1. Goals of this strategy

The goal of this strategy is to develop an organisation-wide system to ensure that all patients are asked about tobacco use as part of every clinical encounter. Such a system would be implemented with the intention that clinicians and care-providers have more opportunity to offer and provide smoking-related care. Such prompts also encourage staff to approach tobacco use as a chronic disease requiring ongoing care similar to that offered to patients identified with other illnesses. In line with the COM-B theory of behaviour change measures such as changing systems improves Opportunity. Attitude and cultural change aims to increase motivation for practice change.

4.2. Evidence for this strategy

Smoker identification prompts have been shown to increase the rate that clinicians intervene with smokers.[(48)] There is significant evidence that implementing a clinic-based tobacco-use identification system increases the rate of smoker identification and facilitates the provision of advice to quit and possibly assistance in quitting. Screening patients for tobacco use and providing care has the potential to increase quit attempts and substantially reduce tobacco-related disease burden and healthcare costs. The US public health service clinical practice guidelines for tobacco makes a strong recommendation that all patients should have their tobacco use status identified at all visits and that brief evidence-based intervention such as the 5As (see *Section 7. Tobacco Dependence Treatments*) be routinely given to all those who are currently using or have recently quit tobacco use.[(49)]

The creation and employment of identification systems or patient registries has been specifically recognised as a means to improve patient care.[(50)] There has been considerable research on the effectiveness of office identification systems that prompt providers and patients regarding desired prevention interventions.[(51)] In relation to tobacco use, large randomized controlled trials of office identification tracking systems for smokers have established that these systems increase both the rate of clinician intervention and the quit rate in a population. In a multi-speciality medical group tobacco use status was significantly and more frequently identified in clinics with access to a registry. There was found to be a significant healthcare quality improvement.[(50)]

Past research has provided strong evidence not only for the effectiveness and cost effectiveness of screening and treating smokers in the course of everyday care but also for the impact of health systems change to support routine intervention.[(32)] The primary care visit is a particularly important time where smoking cessation can be addressed.[(52)] A proposed way to take advantage of this time is to change the electronic medical records or paper-based recording systems for clinicians to have prompts about smoking status and links for patients to cessation services. Other approaches may include expanding vital signs to include tobacco use. Following implementation of these systems, post-intervention, patients were more likely to report being asked about smoking status, being advised to quit and receiving specific advice on how to quit.[(26)]

4.3. Main steps to implementation of this strategy

4.3.1. **Information to assess and record**

In the initial assessment document that patients are provided with on admission/at first encounter questions related to tobacco use need to be early on in documents and ask about

- If they are a smoker
- If they had wanted this visit to provide them with a quit attempt
- If they would like support and information regarding quitting

4.3.2. **Decide on a service-appropriate method of assessment**

Treatment centres will develop a smoke identification system that is appropriate to their current operations to ensure that all patients are asked about their tobacco use at every clinical encounter. There are a range of options for incorporating assessment prompts and methods of documenting smoking status as well as any follow-up care provision into patient records. The first step is identifying the methods that work best for the service.

Smoking Status Assessment Options:

Updating current intake / initial assessment forms

If the service does not currently assess tobacco use at intake or initial assessment, include the recording of tobacco use on existing intake and/or assessment forms. For example:

**Tobacco use:**	**Current**	**Former**	**Never**	*(circle one)*

If tobacco use is already recorded as part of existing assessment:

- Move item for assessing smoking status closer to beginning of assessment to increase chances of it being asked and documented

Tobacco Use stickers

Where patient files are paper-based a sticker system may be employed. Stickers are to be placed on all patient files, which provide a quick and easy visual marker to signify that patient is a current tobacco smoker. For example, in hospital-based systems, stickers could be placed on patient charts, on the first page next to the patient Medical Record Number.

Electronic Prompt
In services that use electronic systems to store patient files and document progress, automated messages to appear on screen when a new file is created on a patient may be programmed. Reminders may also be set to track progress in follow-up consultations.

Subsequent Assessment Options to ensure tobacco-use is obtained:
Set a note in patient file for follow up discussion surrounding smoking once they have identified as a smoker. This note can be on the paper or electronic patient files.

- Record in clinical assessment or daily progress notes of patient NRT use (include type and dosage).
- Repeat assessment will not be necessary in the case of a patient who has never used tobacco or not used tobacco for many years.

4.3.3. Clarify staff responsibility for implementation
All staff members that are responsible for gathering initial information from clients, recording daily habits and subsequent follow up documentation should be informed of the importance of this activity.

4.3.4. Ways to increase awareness of smoker identification system
During the period of implementation of a smoker identification system, it may be helpful to use written, visual and verbal reminders prompting staff to assess and document patient smoking status. Prompts and reminders may vary depending on the systems of documentation each service uses. Examples may include desk reminders, posters or organisation wide emails or memos.
- Desk prompts (see Resource 4.1)
- Brief flyer or email reminders (see Resource 1.4)

4.4. Resources for this strategy

RESOURCES FOR THIS STRATEGY	
Resource 4.1	Desktop reminder to assess smoking status
Resource 1.4	Poster / email reminder to assess smoking status

5. Provide Education and Resources

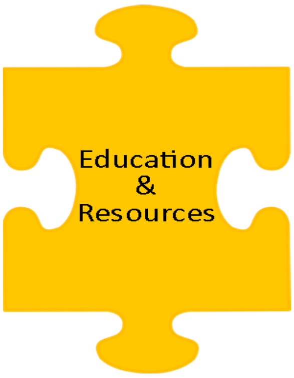

5.1. Goals of this strategy

The provision of education, training and resources is necessary to ensure that staff have the skills and information to assist their clients in making quit attempts.[26, 28] Staff will receive training on smoking cessation techniques through their local smoking cessation training services. For example, in NSW the CCNSW's Tackling Tobacco training is designed specifically to raise awareness of the link between smoking and social disadvantage and is modelled closely on the NSW Health smoking cessation training services for health professionals[53] which focuses on the 5As (Ask, Assess, Advise, Assist, and Arrange follow-up), and appropriate Nicotine Replacement Therapy (NRT) provision. It is designed to encourage and support smoking cessation practices as part of routine care. The Stages of Change theoretical model[8] is used to assess a smoker's motivation to quit and tailor support.

5.2. Evidence for this strategy

Staff in addictions treatment settings have often received little or no training in treating tobacco dependence and many are smokers themselves.[43] Providing training for staff to help patients with tobacco cessation is recommended for substance abuse clinicians.[54] Training should cover screening, assessment and development of treatment plans for tobacco dependence.[55] Staff training increases the frequency, quality and effectiveness with which providers deliver tobacco treatment.[56, 57] Cessation training can also positively influence staff attitudes concerning the value of tobacco treatment.[58, 59] In line with the COM-B theory of behaviour change, measures such as staff training improve Capability and the provision of resources and tools improves Opportunity.

5.3. Main steps to implementation of this strategy

5.3.1. **Clarify staff responsibility for implementation**

All staff members that are responsible for gathering initial information from clients, recording daily habits and subsequent follow up documentation should be informed of the importance of this activity.

5.3.2. **Staff Training**

A number of staff training days will be organised for each state. Depending on the needs of the services, each service will be asked to nominate staff members to attend the training on addressing client smoking and the delivery of smoking cessation care. Training will be provided by accredited trainers with content based on the CCNSW's Tackling Tobacco training program.

Aims of training:

- Raise awareness of the link between smoking and social disadvantage and is designed to encourage and support smoking
- The training is modelled closely on the NSW Health smoking cessation training for health professionals which focuses on the 5As (Ask, Assess, Advise, Assist, and Arrange Follow-up) and appropriate NRT provision
- The Stages of Change theoretical model is used to assess a smoker's motivation to quit and tailor support
- This training will cover how to address smoking in the drug and alcohol setting, motivational interviewing techniques, cessation counselling and how to administer nicotine replacement therapy. Staff will be asked to record in client case notes their smoking status and offer of TNT program enrolment.

Training conduct:

- One-day smoking cessation workshops will be held in each geographic region for service staff to attend. This training will be one of the first occasions that service staff will be exposed to the program, and as such, training will also serve to engage staff in the program and address any concerns they may have.

Trainer assessment

- Trainer knowledge will be assessed by completion of the National Centre for Smoking Cessation and Training knowledge test

5.3.3. **Provision of Resources**

The research team will provide educational material and resources to support implementation of the TNT intervention (contained in the TNT Resource Kit section of the manual). A number of self-help booklets and educational information flyers developed as part of the Cancer Council NSW 'Tackling Tobacco' Program will be provided. Intervention treatment centres will be routinely contacted by the research team to replenish intervention resources during the study period.

5.4. Resources for this strategy

RESOURCES FOR THIS STRATEGY	
Resource 1.1	CCNSW Tackling Tobacco pamphlet "Incorporating smoking cessation into drug and alcohol treatment – Information for staff"
Resource 5.1	CCNSW Tackling Tobacco pamphlet "*Not ready to give up*"
Resource 5.2	CCNSW Tackling Tobacco pamphlet "*Thinking about giving up*"
Resource 5.3	CCNSW Tackling Tobacco pamphlet "*Ready to give up*"
Resource 5.4	CCNSW Tackling Tobacco pamphlet "*Staying a non-smoker*"
Resource 5.5	Quit kits and ordering information

6. Provide Case-Worker and Client Feedback

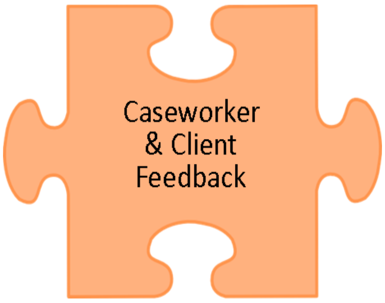

6.1. Goals of this strategy

Computerised feedback has been found to improve the delivery of preventive services in general practice.[60] For the service, newsletters and brief update reports will be used to motivate staff and keep the service engaged with the intervention and project. Where needed, services will be provided with carbon monoxide (CO) monitors to provide motivational feedback to clients regarding their progress during quit attempts. There is also substantial evidence and a theoretical base which suggests that evaluation is important for the successful implementation of health promotion programs. In line with the COM-B theory of behaviour change, providing feedback about behaviour and performance increases knowledge and understanding, facilitating Capability among individuals and organisations.

6.2. Evidence for this strategy

Healthcare providers are receptive to academic feedback.[61] Providing feedback increases the identification and intervention with smokers including the delivery of advice, assistance and arranging follow-up with patients who smoke. Performance goals and expectations can be communicated to organisations in a series of written and verbal formats including newsletters and emails for management and staff, progress reports, and agenda items in team meetings.[50] The use of reminders is also generally effective in assisting in behaviour change of healthcare providers.[62]

Study recruitment goals and performance can be communicated to organisations in a series of written and verbal formats.[50] Tips and news about the study can also accompany this quantitative feedback. The updates can be provided at a number of time points such as prior, during and post intervention. For some variables it will be important to provide fortnightly or monthly updates about recruitment and targets achieved. The use of comparison between other organisations is to facilitate a friendly competition among organisation sites to surpass performance of fellow sites in recruiting more individuals or achieving more aspects of the intervention components.

6.3. Main steps to implementation of this strategy

6.3.1. **Service: Staff Survey Findings**

Prior to the randomisation process where in services were allocated to the intervention or control groups, all participating service staff completed an online survey about current tobacco policies and practices. Services will be fed back aggregated data concerning:

- staff and manager attitudes to the provision of smoking care
- perceptions of current practice regarding smoking and tobacco use
- perceptions of barriers to implementing changes to smoking policy and provision of care

6.3.2. **Service: Intervention newsletter**

An intervention group newsletter that includes feedback on performance will be circulated by the research team to all intervention services. This newsletter will be separate from the general TNT newsletter for all participating services (intervention and control) that will focus on recruitment and other general issues.

6.3.3. **Service: Benchmarking**

By collating recruitment performance indicators for the top performing 10% of services, a benchmark of what recruitment goals can be achieved in a given period will be promoted to services. These benchmark figures will be specific to the size & type of the service (particularly government vs. non-government) and will be circulated via email attachment to the key contact at each site.

Benchmark feedback will include:

- Site recruitment numbers: the proportion of clients recruited into the project
- Visual aids: simple actual, expected and benchmark recruitment number graphs will be created, tracking the performance of each service.
- Comparable with peer performance: each service will have its recruitment performance tracked against the benchmark standard to see how they compare to other services
- Repeated over time: services will receive monthly progress updates during the recruitment phase

6.3.4. **Service: Quitline referrals**

Services will be asked to note their service name on any Quitline fax referral forms that are used throughout the project (see Resource 6.1 – Quitline Fax Referral Form). The number of Quitline referrals made by each service can then be tracked in coordination with Quitline research staff. This information will be provided as feedback to service sites.

6.3.5. **Service: Reminders**

Reminders about the activities required as part of the research project, as well as reminders of content presented in staff training can be circulated to service staff via emails, newsletters and desk top visual prompts. Content can include:

- Quick tips on providing brief advice to quit

- Reminders to record client smoking status in case notes
- Prompts to provide quit smoking pamphlets
- Reminders to use Quitline fax referral forms

6.3.6. **Client: Recording smoking status**

As part of the intervention, services are being encouraged to assess and record smoking status at all clinical encounters. Service providers can use smoking notes in a client's case files to prompt asking the question about smoking and following up on whether the client requires any further information or support about their smoking and interest in quitting.

6.3.7. **Use of quit plans**

Services will be encouraged to complete quit plans with their clients and refer back to these quit plans throughout the period of the client's engagement with the service. The quit plan can be used as a progress feedback tool with clients (see Resource 6.2 – Quit Plan).

6.3.8. **CO monitors**

Carbon monoxide (CO) monitors will be used to verify smoking status at the 6-month follow-up. However, many services have their own CO monitors; where this is the case, services will be encouraged to use these to provide motivational feedback to clients regarding their progress during quit attempts (see Resource 6.3 – CO monitor protocol).

6.4. Resources for this strategy

RESOURCES FOR THIS STRATEGY	
Resource 6.1	Quitline Fax Referral Form
Resource 6.2	TNT Quit Plan
Resource 6.3	CO monitor protocol (including instructions for use)

7. Include Evidence-Based Tobacco Dependence Treatments

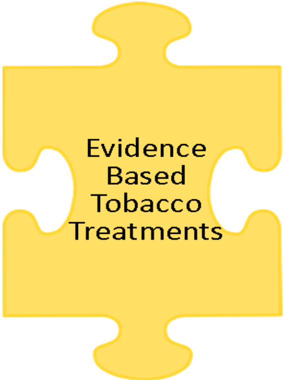

7.1. Goals of this strategy

Tobacco-dependence treatment is both clinically effective and cost-effective.[39] Providing tobacco dependence treatment within the treatment centre setting reduces the cost barriers to cessation.[63] Resources such as QUIT Pack, QUITline and QUIT Coach will be made use of where appropriate. Nicotine Replacement Therapy (NRT) will be provided free of cost during treatment. This intervention will provide a flexible range of NRT options and extends limited NRT subsidies currently available through the Pharmaceutical Benefits Scheme for prescription nicotine patches only. The goal is to ensure that service clients who are smokers have access to effective, evidence-based behavioural support and medication to aid in cessation. The provision of pharmacological interventions to aid smoking cessation enables an increase in services' Opportunity to engage in clinically meaningful smoking care, as well as increasing their Capability to be able to provide cessation support.

7.2. Evidence for this strategy

The majority of evidence supports concurrent treatment for tobacco and other substances. Combining treatment is the most effective way to address concurrent addiction. In substance abuse treatment, studies have shown that smoking cessation is effective with smoking cessation rates ranging from 4.7% at 6 months follow up to 23.4% at one week follow up.[54] Integrating cessation treatment into existing care results in greater engagement and greater use of cessation pharmacotherapy. Tobacco dependence treatment includes, singly or in combination, behavioural and pharmacological interventions such as brief advice and counselling, intensive support and administration of pharmaceuticals that contribute to reducing or overcoming tobacco dependence in individuals and in the population as a whole.[64]

Providing tobacco dependence services for persons with addictions is effective and needs to occur during recovery.[13] Tobacco dependence treatment does not jeopardize recovery from other substances. Smoking cessation interventions delivered during treatment increases the odds of post-treatment abstinence from patient's primary substance abuse[65] and greater engagement and use of

cessation pharmacotherapy.[13] Treatment increases the quality of life of these individuals immediately and in the long-term.[65]

Integrating cessation advice and care into routine medical visits and primary health care services capitalises on existing health professional networks. Simple advice to quit smoking by primary health care professionals significantly increases patient quit rates.[66] This method can also link smokers to other cessation support and tobacco control efforts such as telephone quit lines and pharmacotherapy. Telephone cessation-counselling hotlines, also known as 'quit lines', are widely accessible services for smokers seeking assistance to quit. The most successful quit lines use multiple call-back proactive counselling approaches following smokers up over time.[67, 68] Quit lines can also provide effective information on the use of pharmacological treatments that assist with cessation. Nicotine replacement therapies (NRT) such as patches, lozenges, gum and inhalers are readily available over-the-counter treatments that aid in reducing the withdrawal symptoms during a quit attempt by replacing the nicotine from cigarettes. The use of NRT increases the chances of a smoker successfully quitting by 50 – 70%.[69] Combination treatments pairing nicotine patches with faster acting NRTs or prescription cessation medications can further increase quitting rates.[69]

7.3. Main steps to implementation of this strategy

Staff will receive training on the delivery of evidence-based tobacco dependence treatment (see Section 5: Provide Education and Resources). Resources such as QUIT Pack, QUITline and QUIT Coach will be made use of where appropriate. Nicotine Replacement Therapy (NRT) will be provided free of cost during treatment.

This section of the manual provides a brief overview of these treatments, as well as useful resources covering delivery, use and troubleshooting for the implementation of smoking cessation as part of routine client care.

7.3.1. **Brief Advice – The 5A's**

Brief advice is a standardized procedure involving brief advice to quit smoking with brief counselling about methods recommended for smokers in primary care settings.[70] The approach requires relatively little training, assessment or time, and follows a format of assessing smoking, advising the person to quit, providing assistance with quitting and conducting follow-up or booster sessions.

The following table provides an overview of how to implement each of the 5A's, with links to further resources. The strategy table has been adapted from *"Treating Tobacco Use & Dependence: Clinical Practice Guideline 2008 Update"* US Department of Health and Human Services.

A1. ASK

Systematically identify all tobacco users at every visit

Strategies for Implementation	Resources
**Implement a service-wide system that ensures that, for *every* patient at *every* clinic visit, tobacco use status is queried and documented.**	Section 4: Implement a System of Identifying Smokers

A2. ADVISE

In a *clear, strong and personalised* manner, urge every tobacco user to quit

Strategies for Implementation	Resources
**Clear advice:**<br>"*It is important that you quit smoking now, and I can help you*"<br>"*Cutting down while you are ill is not enough*"<br>"*Occasional or light smoking is still dangerous*"<br><br>**Strong advice:**<br>"*The best thing you can do for your health is to stop smoking, and I would advise you to stop as soon as possible*"<br><br>**Personalised advice:**<br>Tie tobacco use to current situation, for example current symptoms and health concerns; social and economic costs; impact of tobacco use on children and others in the household.	**Resource 7.1:** 5A's poster

A.3 ASSESS

Determine every tobacco user's willingness to make a quit attempt at the time.

Strategies for Implementation	Resources
"Are you willing to give quitting a try?"<br><br>**YES: provide assistance (**see – A4. ASSIST**)**<br><br>**NO: provide motivational interviewing**	**Resource 7.2:** Motivational Interviewing Strategies<br><br>**Resource 7.3:** 5R's – the smoker unwilling to quit

A4. ASSIST

Aid the patient in quitting by providing counselling and medication

Strategies for Implementation	Resources
Looking at the patient's **readiness to change** may help in choosing an effective approach to take.	**Resource 7.4:** Stages of Change Approach – decision branching tool
Help the patient with a **QUIT PLAN**.<br><br>A patient's preparations for quitting may include:<br>Set a date ideally within 2 weeks<br>Tell others about quit plans<br>Anticipate challenges e.g. nicotine withdrawal symptoms<br>Remove tobacco products from your environment	**Resource 6.2:** Quit Plan<br><br>**Resource 7.5:** Nicotine Withdrawal Symptoms
**NICOTINE REPLACEMENT THERAPY**<br>Explain how these medications increase quitting success and reduce withdrawal symptoms.	**Resource 7.6:** NRT Use<br>**Resource 7.7:** NRT Protocol<br>**Resource 7.8:** What if the NRT isn't working?
Provide practical **COUNSELLING** (problem solving / training):<br>*Abstinence*. Striving for total abstinence is essential. Not even a single puff after the quit date.<br>*Past quit experience*. Identifying what helped/hurt in previous quit attempts and build on past success.<br>*Anticipate triggers or challenges*. Discuss how the patient will overcome these (e.g. avoid triggers, alter routines).<br>*Alcohol*. Alcohol is associated with relapse and should be avoided while quitting.<br>*Others in the household*. Quitting is more difficult when there is another smoker in the household. Patients should encourage housemates to quit with them or to not smoke in their presence.	**Resource 7.9:** Relapse & Coping Strategies<br>**Resource 7.5**: Nicotine Withdrawal Symptoms
**EXISTING QUIT RESOURCES**<br>These resources will be provided to services by the TNT team.	**Resource 5.5:** Quit kit<br>**Resources 1.1, 5.1, 5.2, 5.3, 5.4**: CCNSW Tackling Tobacco pamphlets<br>**Resource 5.4**: Quitline information

A5. ARRANGE

Ensure follow-up contacts, either in person or via telephone.

Strategies for Implementation	Resources
**Clients who <u>do</u> have on-going contact with the service:**<br><br>*Timing*: follow-up soon after the quit date, preferably in the first week.<br><br>*Actions during follow-up contact*:<br>Identify challenges in the immediate future.<br>Assess medication use and problems.<br>Remind patients of quitline support.<br>Address tobacco use at next clinical visit (treat tobacco use as a chronic disease).<br>For those patients who are abstinent, congratulate them on their success.<br>If tobacco use has occurred, review circumstance and elicit recommitment to total abstinence.	**Resource 6.2:** Quit Plan<br>**Resource 7.9:** Relapse & Coping Strategies<br>**Resource 7.5:** Nicotine Withdrawal Symptoms<br>**Resource 7.7:** NRT Protocol<br>**Resource 7.8:** What if the NRT isn't working?
**Clients who <u>do not</u> have on-going contact with the service:**<br>Arrange for follow-up contact with other existing quit services, such as Quitline, by using the Quitline Fax Referral form. If the client has contact with a GP, you might also consider sending a letter to their GP to inform them of quit interest, attempts and need for further follow-up.	**Resource 6.1:** Quitline Fax Referral Form<br>**Resource 8.1:** GP Letter

7.3.2. **Use of existing QUIT resources**

Resources such as QUIT Pack, QUITline and QUIT Coach will be made use of where appropriate.

- Telephone helplines or quitlines are effective. A simple referral requires little effort from treatment professionals.
- Telephone support aftercare: Tobacco cessation is strengthened by revisitation in aftercare programs.

7.3.3. **Provision of Nicotine Replacement Therapy (NRT)**

Nicotine Replacement Therapy (NRT) will be provided free of cost during treatment. This intervention will provide a flexible range of NRT options. An NRT log will be provided to track usage of the NRT provided to the service (see Resource 7.10 – NRT log). NRT can be in the form of a gum, patch, nasal spray, inhaler and a lozenge. These are first line therapy, and varenicline or bupropion (prescription only; not provided as part of TNT) can be used along or as an adjunct to NRT. Many smokers are unaware of these effective cessation methods and most underestimate their benefit.

Staff and patients are to be provided with access to and encouragement in using medications and nicotine replacement therapies that will enable them to quit smoking. Individuals that are using these supports already should be further encouraged to adhere to the treatment in order to maintain their quit attempt as it will increase their likelihood of successfully quitting. The use of pharmacological support is related to the behaviour change technique 11.1.

7.4. Resources for this strategy

**RESOURCES FOR THIS STRATEGY**	
Resource 1.1	CCNSW Tackling Tobacco pamphlet "*Incorporating smoking cessation into drug and alcohol treatment – Information for staff*"
Resource 5.1	CCNSW Tackling Tobacco pamphlet "*Not ready to give up*"
Resource 5.2	CCNSW Tackling Tobacco pamphlet "*Thinking about giving up*"
Resource 5.3	CCNSW Tackling Tobacco pamphlet "*Ready to give up*"
Resource 5.4	CCNSW Tackling Tobacco pamphlet "*Staying a non-smoker*"
Resource 5.5	Quit kits and ordering information
Resource 6.1	Quitline Fax Referral Form
Resource 6.2	TNT Quit Plan
Resource 7.1	5A's poster
Resource 7.2	Motivational Interviewing strategies
Resource 7.3	The 5R's (for the smoker unwilling to quit)
Resource 7.4	Stage of Change approach – decision branching
Resource 7.5	Nicotine Withdrawal – What is it?
Resource7.6	NRT Use
Resource 7.7	NRT protocol
Resource 7.8	What if the NRT is not working?

Resource 7.9	Relapse and coping strategies
Resource 7.10	NRT use log (tracking sheet for NRT provided to service by TNT)
Resource 8.1	Letter to GP

8. Maintenance and Follow-Up

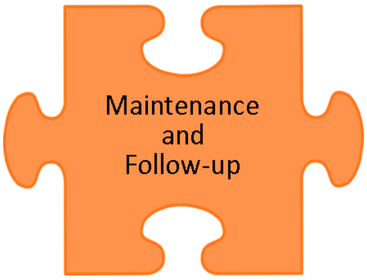

8.1. Goals of this strategy

The goal of this strategy is to ensure that clients have a plan in place to maintain cessation or follow-up on quit interest and intentions upon discharge from the service. This may include sending smoking treatment summaries to the client's primary healthcare provider, or linking the client with follow-up services such as Quitline.

8.2. Evidence for this strategy

Most smokers make repeated quit attempts before stopping for good. Arranging follow-up for post-discharge care can help smokers maintain cessation, or plan for their next quit attempt if they have relapsed. Ideally, follow-up care should be arranged for within one week of the discharge date. Hospital-based research has found that a smoking treatment summary in the discharge plan aids cessation maintenance.[71] The provision of printed self-help materials upon discharge may be of assistance to some clients.[72]

Smokers who are at high risk of relapse may require more intensive assistance. Prompting a primary healthcare provider involved in a client's care may assist in maintenance of cessation or quit interest. Simple advice to quit smoking by primary health care professionals significantly increases patient quit rates.[66] Proactive telephone call-back counselling services are also an effective way of ensuring follow-up client care. 'Quitlines' are widely accessible services for smokers seeking assistance to quit. The most successful quit lines use multiple call-back proactive counselling approaches following smokers up over time.[67, 68]

8.3. Main steps to implementation of this strategy

8.3.1 **Quit Plan**

Services will be encouraged to work through a Quit Plan (see Resource 6.2) with their clients and include a copy of this in their case notes, so that progress can be tracked over time. A copy of the quit plan should also be provided to the client upon discharge from the service, so they can refer to it in order to maintain cessation, or to help plan future quit attempts.

8.3.2 **Linking to Primary Healthcare Providers**

With the client's permission, a copy of their smoking information with instructions for post-discharge management will be faxed to their primary health care provider (see Resource 8.1 – Letter to GP).

8.3.3 Quitline Fax Referrals

Clients will be offered to be linked to smoking cessation services, in particular the Quitline. Services will be asked to use Quitline fax referral forms, the use of which can be tracked over the lifespan of the project (see Resource 6.1).

8.4. Resources for this strategy

RESOURCES FOR THIS STRATEGY	
Resource 6.1	Quitline Fax Referral Form
Resource 6.2	TNT Quit Plan
Resource 8.1	Letter to GP

References

1. MICHIE S, VAN STRALEN MM, WEST R. The behaviour change wheel: a new method for characterising and designing behaviour change interventions. Implementation science : IS. 2011;**6**:42.
2. DAMSCHRODER LJ, ARON DC, KEITH RE, KIRSH SR, ALEXANDER JA, LOWERY JC. Fostering implementation of health services research findings into practice: a consolidated framework for advancing implementation science. Implementation science : IS. 2009;**4**:50.
3. AUSTRALIAN INSTITUTE OF HEALTH AND WELFARE. 2010 National Drug Strategy Household Survey Report. Canberra: AIHW 2011 Contract No.: Cat. no. PHE 145.
4. NATIONAL PREVENTATIVE HEALTH TASKFORCE. Australia: The healthiest country by 2020 - National Preventative Health Strategy - the roadmap for action: Commonwealth of Australia 2009.
5. BAKER A, IVERS RG, BOWMAN J, BUTLER T. Where there's smoke, there's fire: high prevalence of smoking among some sub-populations and recommendations for intervention. Drug and alcohol review. 2006;**25**:85-96.
6. KELLY PJ, BAKER AL, DEANE FP, KAY-LAMBKIN FJ, BONEVSKI B, TREGARTHEN J. Prevalence of smoking and other health risk factors in people attending residential substance abuse treatment. Drug and alcohol review. 2012;**31**:638-44.
7. DEPARTMENT OF HEALTH. Smoking Kills: A White Paper on Tobacco. London: The Stationary Office; 1998.
8. PROCHASKA JJ, ROSSI JS, REDDING CA, et al. Depressed smokers and stage of change: implications for treatment interventions. Drug and alcohol dependence. 2004;**76**:143-51.
9. RANDALL D, DEGENHARDT L, VAJDIC CM, et al. Increasing cancer mortality among opioid-dependent persons in Australia: a new public health challenge for a disadvantaged population. Australian and New Zealand journal of public health. 2011;**35**:220-5.
10. HAYS JT, SCHROEDER DR, OFFORD KP, et al. Response to nicotine dependence treatment in smokers with current and past alcohol problems. Annals of behavioral medicine. 1999;**21**:244-50.
11. BURLING TA, SEIDNER BURLING A, LATINI D. A controlled smoking cessation trial for substance-dependent inpatients. Journal of consulting and clinical psychology. 2001;**69**:295.
12. KALMAN D, HAYES K, COLBY SM, EATON CA, ROHSENOW DJ, MONTI PM. Concurrent versus delayed smoking cessation treatment for persons in early alcohol recovery: a pilot study. Journal of substance abuse treatment. 2001;**20**:233-8.
13. JOSEPH AM, WILLENBRING ML, NUGENT SM, NELSON DB. A randomized trial of concurrent versus delayed smoking intervention for patients in alcohol dependence treatment. Journal of studies on alcohol and drugs. 2004;**65**:681.
14. REID MS, FALLON B, SONNE S, et al. Smoking cessation treatment in community-based substance abuse rehabilitation programs. Journal of substance abuse treatment. 2008;**35**:68-77.
15. SHOPTAW S, ROTHERAM-FULLER E, YANG X, et al. Smoking cessation in methadone maintenance. Addiction. 2002;**97**:1317-28.
16. STEIN MD, WEINSTOCK MC, HERMAN DS, ANDERSON BJ, ANTHONY JL, NIAURA R. A smoking cessation intervention for the methadone-maintained. Addiction. 2006;**101**:599-607.
17. NSW MINISTRY OF HEALTH. The Guide for the Management of Nicotine Dependent Inpatients. Gladesville, NSW: Better Health Centre; 2002.
18. FIORE M. Treating tobacco use and dependence: 2008 update: Clinical practice guideline: DIANE Publishing; 2008.
19. BOWMAN J, WALSH R. Smoking intervention within alcohol and other drug treatment services: a selective review with suggestions for practical management. Drug and alcohol review. 2003;**22**:73-82.
20. CURRIE SR, NESBITT K, WOOD C, LAWSON A. Survey of smoking cessation services in Canadian addiction programs. Journal of substance abuse treatment. 2003;**24**:59-65.
21. RATSCHEN E, BRITTON J, MCNEILL A. The smoking culture in psychiatry: time for change. The British Journal of Psychiatry. 2011;**198**:6-7.
22. WALSH RA, BOWMAN JA, TZELEPIS F, LECATHELINAIS C. Smoking cessation interventions in Australian drug treatment agencies: a national survey of attitudes and practices. Drug and alcohol review. 2005;**24**:235-44.

23. FRIEDMANN PD, JIANG L, RICHTER KP. Cigarette smoking cessation services in outpatient substance abuse treatment programs in the United States. Journal of substance abuse treatment. 2008;**34**:165-72.
24. HALL SM, PROCHASKA JJ. Treatment of smokers with co-occurring disorders: emphasis on integration in mental health and addiction treatment settings. Annual Review of Clinical Psychology. 2009;**5**:409.
25. HUNT JJ, CUPERTINO AP, GARRETT S, FRIEDMANN PD, RICHTER KP. How is tobacco treatment provided during drug treatment? Journal of substance abuse treatment. 2012;**42**:4-15.
26. FIORE MC, KELLER PA, CURRY SJ. Health system changes to facilitate the delivery of tobacco-dependence treatment. American journal of preventive medicine. 2007;**33**:S349-S56.
27. SHARP JR, SCHWARTZ S, NIGHTINGALE T, NOVAK S. Targeting nicotine addiction in a substance abuse program. Science & practice perspectives / a publication of the National Institute on Drug Abuse, National Institutes of Health. 2003;**2**:33-40.
28. ZIEDONIS DM, WANG X, LI T, et al. Addressing Tobacco Through Organizational Change in a Hospital-Based Mental Health Center in China: The Intervention and Lessons Learned in a Pilot Implementation Project. Journal of Dual Diagnosis. 2012;**8**:148-57.
29. SKINNER N. Organisational Change. In: Skinner N, Roche AM, O'Connor J, editors. Workfore Development TIPS (Theory Into Practice Strategies): A Resource Kit for the Alcohol and Other Drugs Field. Adelaide: National Centre for Education and Training on Addiction (NCETA), Flinders University; 2005.
30. JOHNSON M, AUSTIN MJ. Evidence-based practice in the social services: Implications for organizational change. Administration in Social Work. 2006;**30**:75-104.
31. HEWARD S, HUTCHINS C, KELEHER H. Organizational change—key to capacity building and effective health promotion. Health Promotion International. 2007;**22**:170-8.
32. KELLER PA, FIORE MC, CURRY SJ, ORLEANS CT. Systems change to improve health and health care: lessons from addressing tobacco in managed care. Nicotine & tobacco research : official journal of the Society for Research on Nicotine and Tobacco. 2005;**7 Suppl 1**:S5-8.
33. FREUND M, CAMPBELL E, PAUL C, et al. Increasing hospital-wide delivery of smoking cessation care for nicotine-dependent in-patients: a multi-strategic intervention trial. Addiction. 2009;**104**:839-49.
34. BECAN JE, KNIGHT DK, FLYNN PM. Innovation adoption as facilitated by a change-oriented workplace. J Subst Abuse Treat. 2012;**42**:179-90.
35. LEHMAN WE, SIMPSON DD, KNIGHT DK, FLYNN PM. Integration of treatment innovation planning and implementation: Strategic process models and organizational challenges. Psychology of Addictive Behaviors. 2011;**25**:252.
36. ELVING WJ. The role of communication in organisational change. Corporate Communications: An International Journal. 2005;**10**:129-38.
37. QUEENSLAND GOVERNMENT. Change Management Best Practices Guide.
38. HOFFMAN KA, GREEN CA, FORD JH, 2ND, WISDOM JP, GUSTAFSON DH, MCCARTY D. Improving quality of care in substance abuse treatment using five key process improvement principles. The journal of behavioral health services & research. 2012;**39**:234-44.
39. BONEVSKI B, BOWMAN J, RICHMOND R, et al. Turning of the tide: changing systems to address smoking for people with a mental illness. Mental Health and Substance Use. 2011;**4**:116-29.
40. HEHIR A, INDIG D, PROSSER S, ARCHER V. Implementation of a smoke-free policy in a high secure mental health inpatient facility: staff survey to describe experience and attitudes. BMC Public Health. 2013;**13**:315.
41. EBY L, GEORGE K, BROWN BL. Going tobacco-free: predictors of clinician reactions and outcomes of the NY State Office of Alcoholism and Substance Abuse Services tobacco-free regulation. J Subst Abuse Treat. 2013;**44**:280-7.
42. RICHTER KP, CHOI WS, ALFORD DP. Smoking policies in U.S. outpatient drug treatment facilities. Nicotine & tobacco research : official journal of the Society for Research on Nicotine and Tobacco. 2005;**7**:475-80.
43. WILLIAMS JM, FOULDS J, DWYER M, et al. The integration of tobacco dependence treatment and tobacco-free standards into residential addictions treatment in New Jersey. J Subst Abuse Treat. 2005;**28**:331-40.

44. KOTZ MM. A smoke-free chemical dependency unit. The Cleveland Clinic experience. J Subst Abuse Treat. 1993;**10**:125-31.
45. BURLING TA, MARSHALL GD, SEIDNER AL. Smoking cessation for substance abuse inpatients. Journal of substance abuse. 1991;**3**:269-76.
46. RUSTIN TA. Incorporating nicotine dependence into addiction treatment. Journal of addictive diseases. 1998;**17**:83-108.
47. GUYDISH J, ZIEDONIS D, TAJIMA B, et al. Addressing Tobacco Through Organizational Change (ATTOC) in residential addiction treatment settings. Drug and alcohol dependence. 2012;**121**:30-7.
48. AHLUWALIA JS, GIBSON CA, KENNEY RE, WALLACE DD, RESNICOW K. Smoking status as a vital sign. Journal of General Internal Medicine. 1999;**14**:402-8.
49. FIORE MC. US public health service clinical practice guideline: treating tobacco use and dependence. Respiratory care. 2000;**45**:1200-62.
50. ROSKI J, JEDDELOH R, AN L, et al. The impact of financial incentives and a patient registry on preventive care quality: increasing provider adherence to evidence-based smoking cessation practice guidelines. Prev Med. 2003;**36**:291-9.
51. MCAFEE T, GROSSMAN R, DACEY S, MCCLURE J. Capturing tobacco status using an automated billing system: steps toward a tobacco registry. Nicotine & tobacco research : official journal of the Society for Research on Nicotine and Tobacco. 2002;**4 Suppl 1**:S31-7.
52. BOYLE R, SOLBERG L, FIORE M. Use of electronic health records to support smoking cessation. The Cochrane database of systematic reviews. 2011:CD008743.
53. ZWAR N, RICHMOND R, BORLAND R, STILLMAN S, CUNNINGHAM M, LITT J. Smoking cessation guidelines for Australian general practice. Canberra: Australian Government; 2004.
54. BACA CT, YAHNE CE. Smoking cessation during substance abuse treatment: what you need to know. J Subst Abuse Treat. 2009;**36**:205-19.
55. ZIEDONIS DM, ZAMMARELLI L, SEWARD G, et al. Addressing tobacco use through organizational change: a case study of an addiction treatment organization. Journal of psychoactive drugs. 2007;**39**:451-9.
56. PAYNE TJ, GAUGHF NW, SUTTON MJ, et al. The impact of brief tobacco treatment training on practice behaviours, self-efficacy and attitudes among healthcare providers. International journal of clinical practice. 2014;**68**:882-9.
57. SUTTON MJ, PAYNE TJ, GAUGHF NW, et al. Tobacco dependence treatment: influence of training experiences on clinical activities among otolaryngologists. The Laryngoscope. 2013;**123**:3005-9.
58. KATZ DA, HOLMAN J, JOHNSON S, et al. Implementing smoking cessation guidelines for hospitalized veterans: effects on nurse attitudes and performance. J Gen Intern Med. 2013;**28**:1420-9.
59. SHEFFER CE, BARONE CP, ANDERS ME. Training health care providers in the treatment of tobacco use and dependence: pre- and post-training results. Journal of evaluation in clinical practice. 2009;**15**:607-13.
60. BONEVSKI B, SANSON-FISHER R, CAMPBELL E, CARRUTHERS A, REID A, IRELAND M. Randomized controlled trial of a computer strategy to increase general practitioner preventive care. Preventive medicine. 1999;**29**:478-86.
61. CURRY SJ, KELLER PA, ORLEANS CT, FIORE MC. The role of health care systems in increased tobacco cessation. Annu Rev Public Health. 2008;**29**:411-28.
62. GRIMSHAW JM, SHIRRAN L, THOMAS R, et al. Changing provider behavior: an overview of systematic reviews of interventions. Medical care. 2001:II2-II45.
63. BONEVSKI B, BRYANT J, PAUL C. Encouraging smoking cessation among disadvantaged groups: A qualitative study of the financial aspects of cessation. Drug and alcohol review. 2011;**30**:411-8.
64. RAW M, REGAN S, RIGOTTI NA, MCNEILL A. A survey of tobacco dependence treatment services in 36 countries. Addiction. 2009;**104**:279-87.
65. PROCHASKA JJ, DELUCCHI K, HALL SM. A meta-analysis of smoking cessation interventions with individuals in substance abuse treatment or recovery. Journal of consulting and clinical psychology. 2004;**72**:1144.
66. STEAD LF, BUITRAGO D, PRECIADO N, SANCHEZ G, HARTMANN-BOYCE J, LANCASTER T. Physician advice for smoking cessation. Cochrane Database of Systematic Reviews. 2013.
67. CUMMINS SE, BAILEY L, CAMPBELL S, KOON-KIRBY C, ZHU S-H. Tobacco cessation quitlines in North America: a descriptive study. Tobacco Control. 2007;**16**:i9-i15.

68. STEAD LF, PERERA R, LANCASTER T. A systematic review of interventions for smokers who contact quitlines. Tobacco Control. 2007;**16**:i3-i8.
69. STEAD LF, PERERA R, BULLEN C, et al. Nicotine replacement therapy for smoking cessation. Cochrane Database of Systematic Reviews 2012.
70. RAW M, MCNEILL A, WEST R. Smoking Cessation Guidelines for Health Professionals—A guide to effective smoking cessation interventions for the health care system. Thorax. 1998;**53**:S1-S18.
71. FREUND M, CAMPBELL E, PAUL C, SAKROUGE R, WIGGERS J. Smoking care provision in smoke-free hospitals in Australia. Preventive medicine. 2005;**41**:151-8.
72. HARTMANN-BOYCE J, LANCASTER T, STEAD L. Print-based self-help interventions for smoking cessation. Cochrane Database of Systematic Reviews 2014.
73. THE CANCER COUNCIL QUEENSLAND, QUEENSLAND HEALTH. Smokeree Policy Guidelines for Workplaces. Brisbane: The Cancer Council Queensland 2006.
74. PROCHASKA JO, DICLEMENTE CC. Stages and processes of self-change of smoking: toward an integrative model of change. J Consult Clin Psychol. 1983;**51**:390-5.

TNT Project Contacts

Associate Professor Billie Bonevski
Chief Investigator
Phone: (02) 40335710
Email: billie.bonevski@newcastle.edu.au

Ashleigh Guillaumier
Project Manager
Phone: (02) 40335718
Email: Ashleigh.guillaumier@newcastle.edu.au

Eliza Skelton
PhD Candidate
Phone: (02) 40335711
Email: Eliza.Skelton@newcastle.edu.au

Project Toll Free Number (free call from landlines)
1800 993 603

Postal Address
CTNMH
PO Box 833
Newcastle NSW 2300

TNT Resource Kit

Resource 1.1: CCNSW Tackling Tobacco Pamphlet – "Incorporating smoking cessation into drug and alcohol treatment – Information for staff"

Available at: http://askthequestion.com.au/wp-content/uploads/2013/08/CAN10471_TT_Factsheet_Treatment_2.pdf

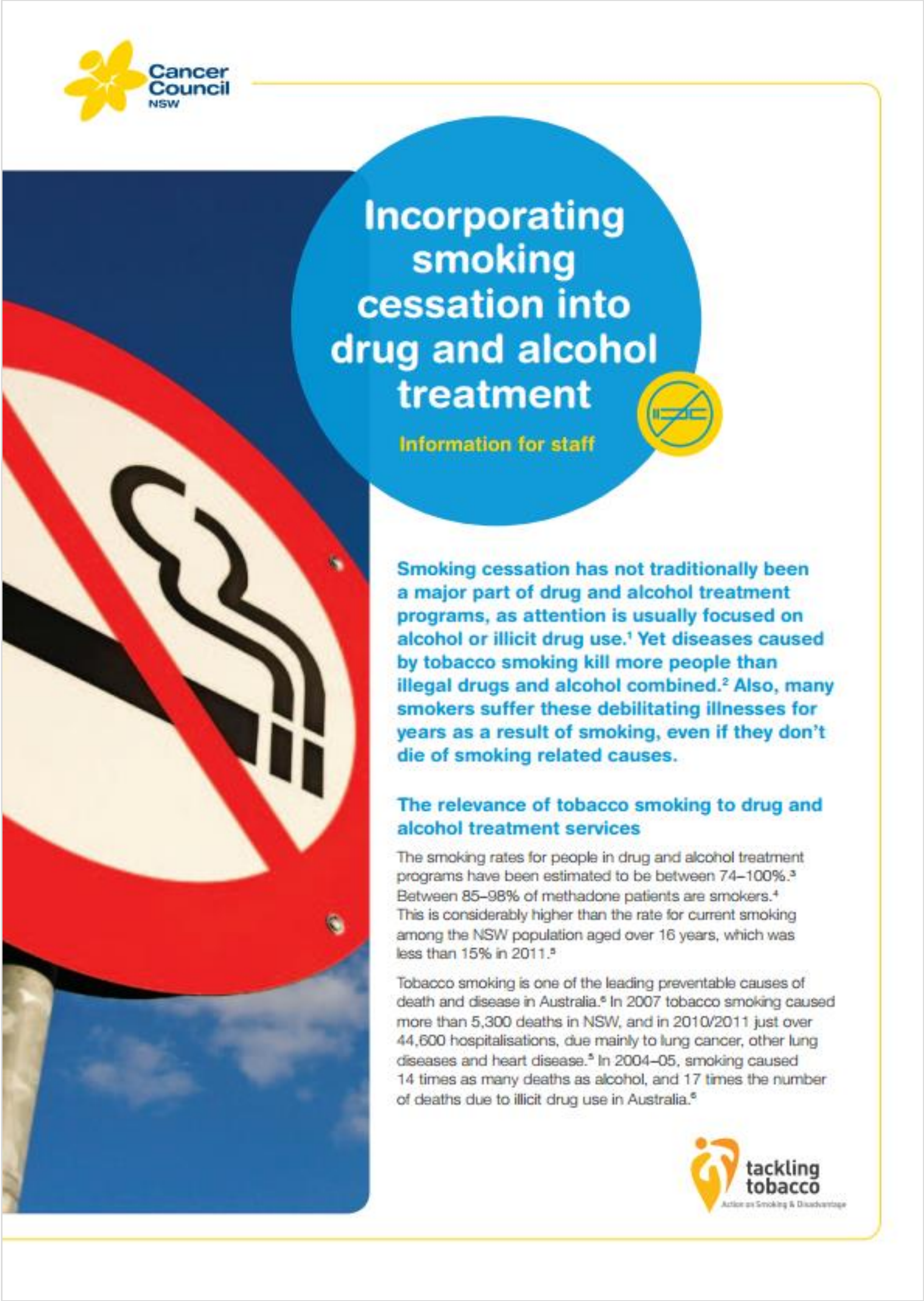

Cancer Council NSW

Incorporating smoking cessation into drug and alcohol treatment

Information for staff

Smoking cessation has not traditionally been a major part of drug and alcohol treatment programs, as attention is usually focused on alcohol or illicit drug use.[1] Yet diseases caused by tobacco smoking kill more people than illegal drugs and alcohol combined.[2] Also, many smokers suffer these debilitating illnesses for years as a result of smoking, even if they don't die of smoking related causes.

The relevance of tobacco smoking to drug and alcohol treatment services

The smoking rates for people in drug and alcohol treatment programs have been estimated to be between 74–100%.[3] Between 85–98% of methadone patients are smokers.[4] This is considerably higher than the rate for current smoking among the NSW population aged over 16 years, which was less than 15% in 2011.[5]

Tobacco smoking is one of the leading preventable causes of death and disease in Australia.[6] In 2007 tobacco smoking caused more than 5,300 deaths in NSW, and in 2010/2011 just over 44,600 hospitalisations, due mainly to lung cancer, other lung diseases and heart disease.[5] In 2004–05, smoking caused 14 times as many deaths as alcohol, and 17 times the number of deaths due to illicit drug use in Australia.[6]

tackling tobacco

Action on Smoking & Disadvantage

Resource 1.2: **Agenda template** for TNT briefing staff meeting

---

Date: dd/mm/yyyy

Agenda

Item No.	Agenda Item	Discussion
**1.**	**Attendance/Apologies:**<br>1.1. Attendance<br>1.2. Apologies	**1.1 Attendees** –<br><br>**1.2 Apologies** –
**2.**	**Action Items from the Previous Minutes**	
**3.**	**TNT project**	
**4.**	**AOB / Next Meeting**	Next meeting:

<u>Resource 1.3</u>: Newsletter article templates for TNT

The following templates have been adapted from The Cancer Council Queensland's *"Smokefree Policy Guide for Workplaces"*(73) available for download at: http://www.health.qld.gov.au/atod/documents/smokefreepolicy_work.pdf

Article 1 – <u>Informing staff of changes to tobacco policy</u>

Going Smokefree

(name of organization) is committed to providing employees with a healthy environment which encourages high staff morale and productivity and protects the health of all employees.

With this in mind, we are proposing that *(name of organisation)* creates a smokefree policy. This policy will help to promote the health and safety of all employees and clients.

(name of organization)'s management team is setting up a smokefree policy committee which will be made up of *(insert number)* management, employee and union representatives.

The committee welcomes any suggestions or questions that you may have. Please direct these to *(insert name).*

We will keep you up-to-date with the progress of *(name of organisation)'s* new smokefree policy.

Article 2 – <u>Promote implementation of tobacco policy</u>

Ready, set, go smokefree

(Name of organisation)'s much awaited smokefree policy is ready to go!

The smokefree policy will officially commence on *(insert date)*.

The policy will see *all areas/most areas* within the *(name of organisation)*'s premises become smokefree.

A copy of the new smokefree policy will be distributed to all staff. Signage has been displayed around the premises and we appreciate your assistance and cooperation in making *(name of organisation)* a smokefree, healthy environment.

<u>Resource 1.4</u>: Reminder email or flyer prompts for changes to tobacco-related polices & practices

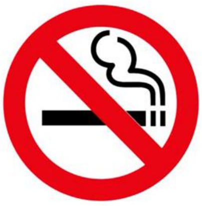

Just a reminder that *(insert organisation name)* is now smokefree.

You can find our written smokefree policy at *(insert location of policy document)*.

<u>Remember:</u>

- Ask every client if they smoke & record in notes
- Offer smokers help to quit (quit plan, NRT etc.)
- Arrange follow-up (next appointment, quitline etc.)

Resource 2.1: Support Champion "Identifier" Poster

TNT TACKLING NICOTINE TOGETHER

Support Champion

Name has been identified as the TNT project Support Champion!

If you have any questions about the TNT project, or would like some support in implementing smoking cessation care with clients, please ask me.

<u>Resource 3.1</u>: CCNSW "Tackling Tobacco Policy Toolkit"

Available at: http://askthequestion.com.au/wp-content/uploads/2011/05/CAN-1023-Addressing-Smoking-Toolkit.pdf

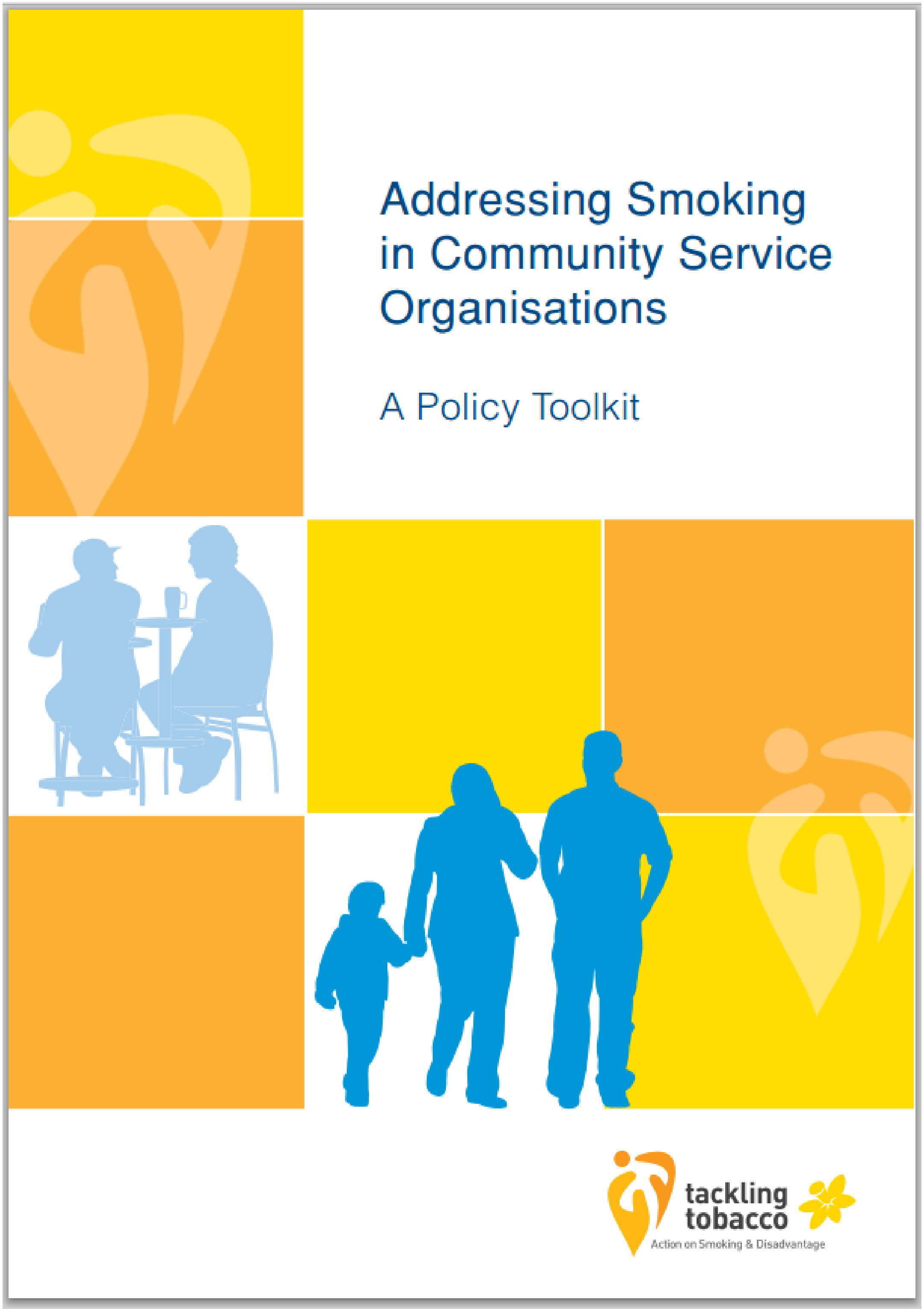

Resource 4.1: Desktop reminder to assess smoking status

The following desktop reminder can be folded in half to display the smoking status check reminder to service staff.

Remember to record in case notes!

Have you asked your client about their smoking status?

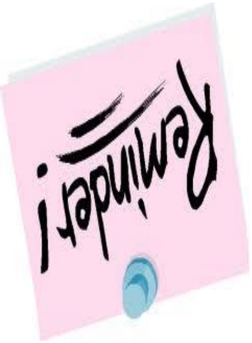

TNT TACKLING NICOTINE TOGETHER

Are you a smoker who is interested in quitting? Ask me how I can help.

<u>Resource 5.1</u>: CCNSW Tackling Tobacco pamphlet – "Not ready to give up"

Available at: http://askthequestion.com.au/wp-content/uploads/2011/05/Booklet-1.pdf

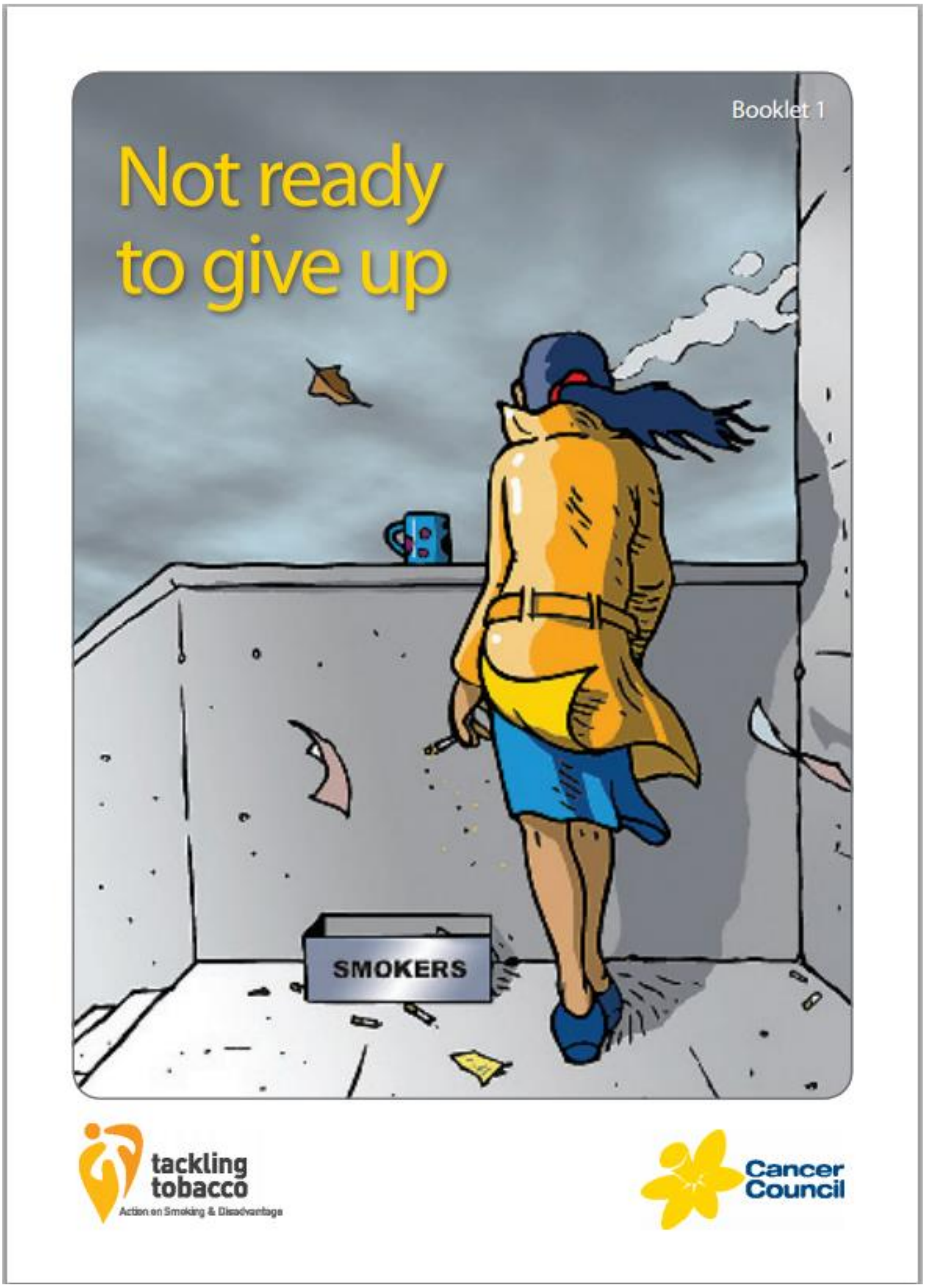

<u>Resource 5.2</u>: CCNSW Tackling Tobacco pamphlet – “Thinking about giving up”

Available at: http://askthequestion.com.au/wp-content/uploads/2011/05/Booklet-2-.pdf

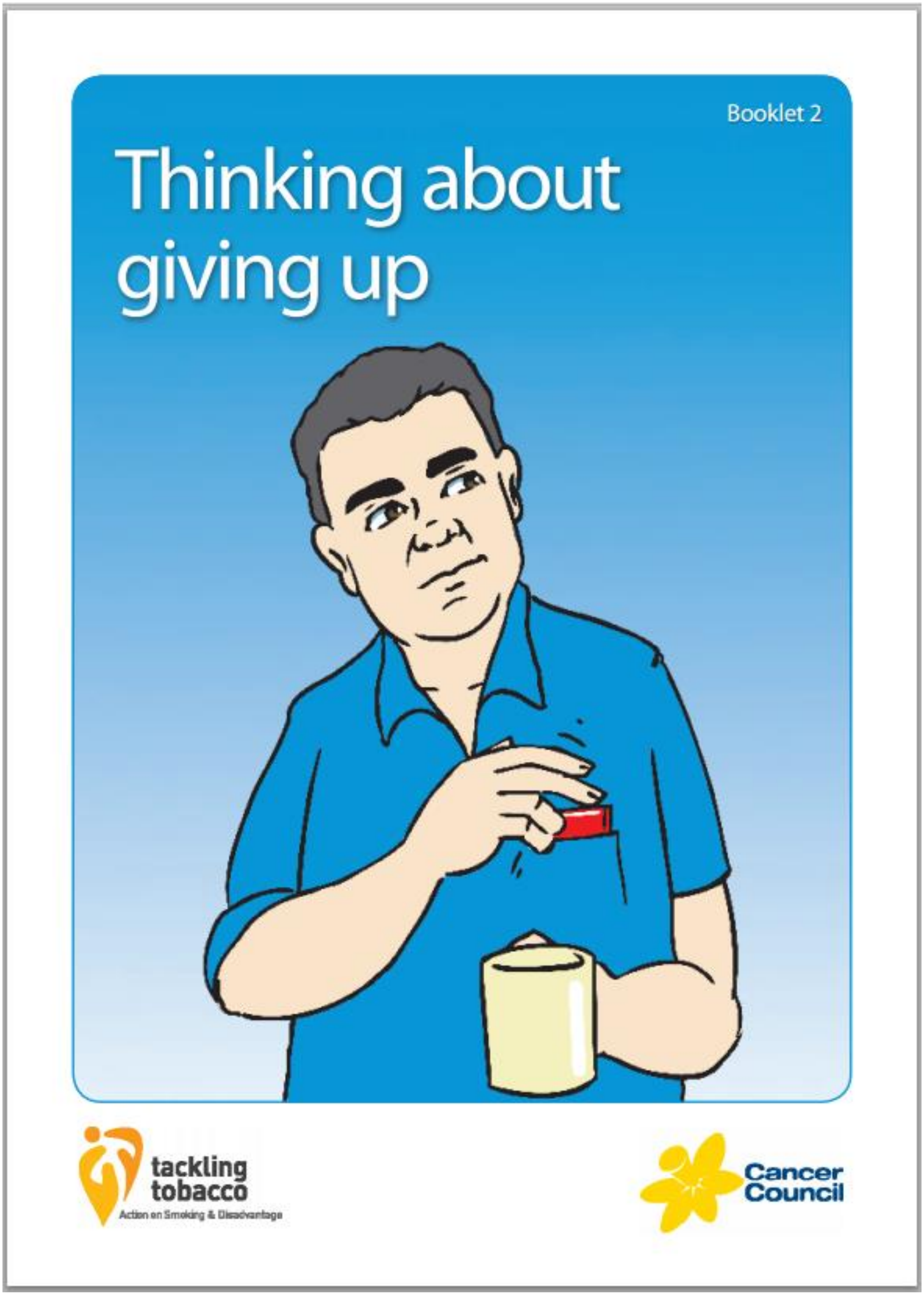

Resource 5.3: CCNSW Tackling Tobacco pamphlet – "Ready to give up"

Available at: http://askthequestion.com.au/wp-content/uploads/2011/05/Booklet-3.pdf

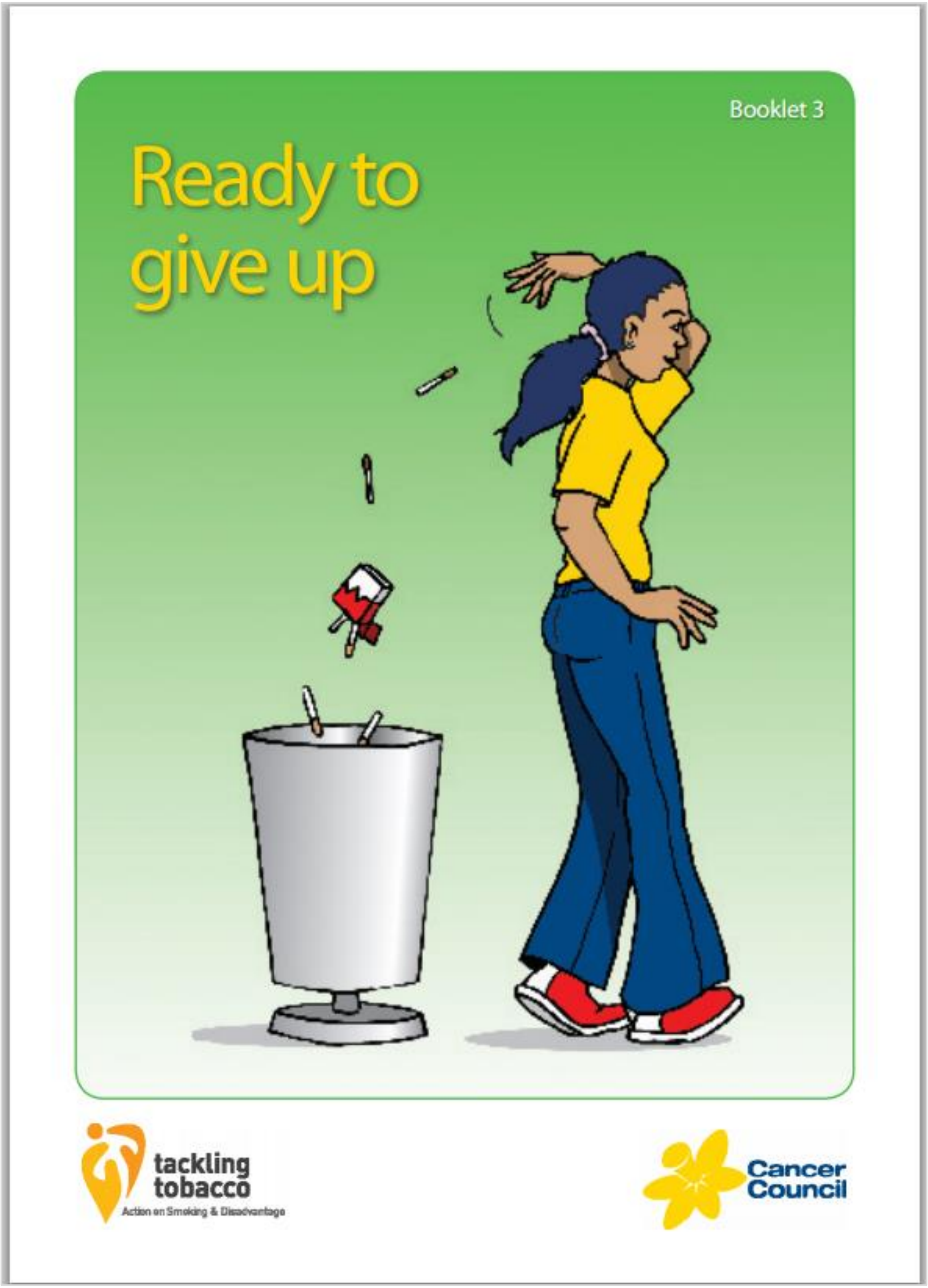

Resource 5.4: CCNSW Tackling Tobacco pamphlet – "Staying a non-smoker"

Available at: http://askthequestion.com.au/wp-content/uploads/2011/05/Booklet-4.pdf

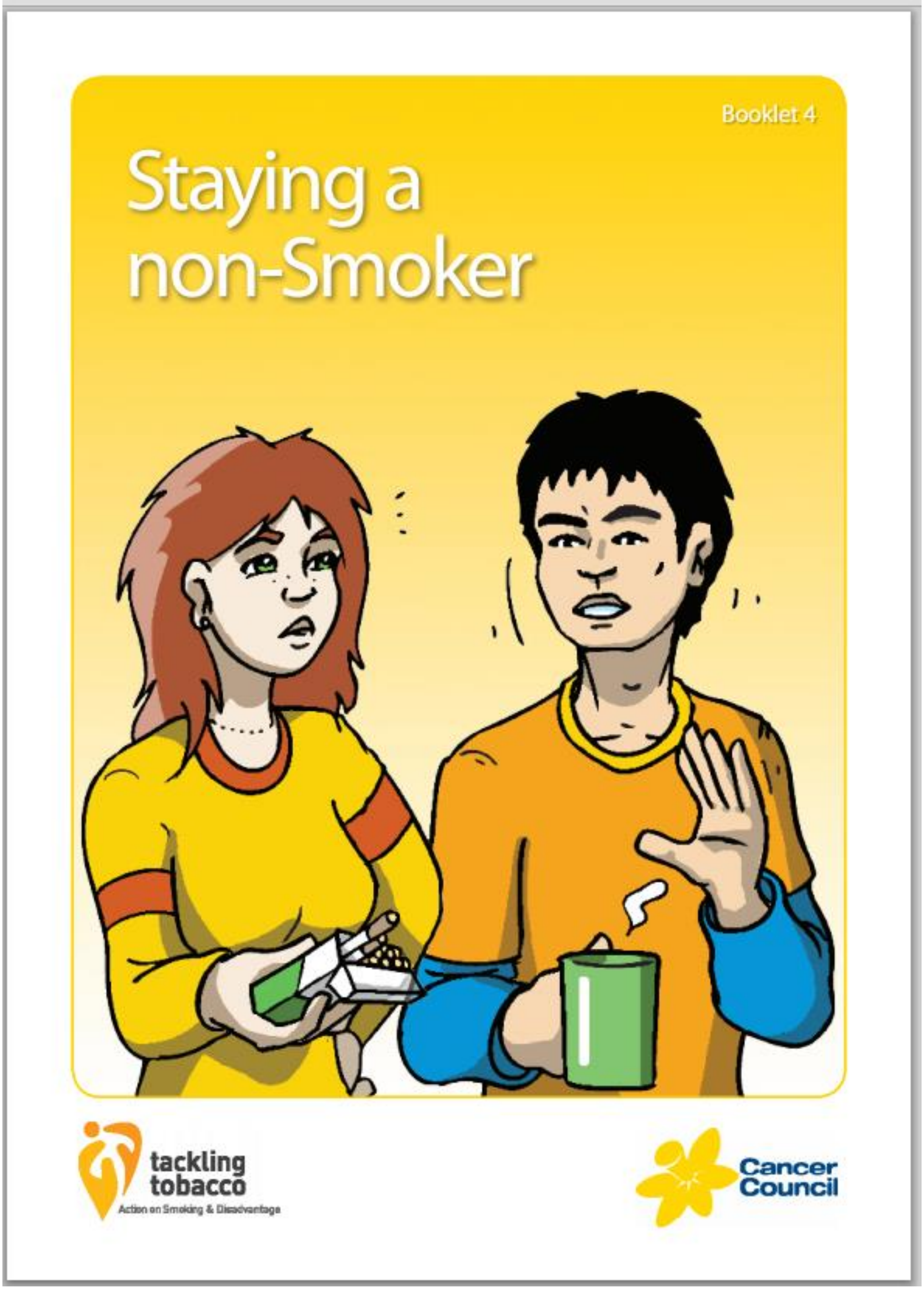

Quit Kit / Quit Pack Ordering Information

***Quit kits can be ordered from the Quitline **13 78 48**

NSW
Available online at:
http://www.icanquit.com.au/further-resources/online-quit-kit

QLD
Resource order form:
http://www.health.qld.gov.au/quitsmoking/documents/quit-orderform.pdf

ACT
Cancer Council ACT
Ph: (02) 6257 9999
E: tobaccocontrol@actcancer.org

Australian Government Campaign Resources
Request form available at:
http://www.quitnow.gov.au/internet/quitnow/publishing.nsf/Content/fact-sheets

Resource 6.1: Quitline Fax Referral Form

Available at:
http://www.quitnow.gov.au/internet/quitnow/publishing.nsf/Content/C267B0382618D7ECCA257A0D001F11DB/$File/smoking%20cessation%20form%20-%20August%202013.pdf

Smoking Cessation Referral Form

Last update August 2013

For use by health professionals to refer patients to Quitline

Fax Numbers:
ACT & NSW (02) 9361 8011 **NT** (07) 3837 5914 **Qld** (07) 3259 8217 **SA** (08) 8291 4280 **Tas** (03) 6242 8111 **Vic** (03) 9635 5520 **WA** (08) 9442 5020

Referrer Details

From: ______________________________

Address: ______________________________

Phone: ______________________________

Fax: ______________________________

Health Professional: ☐ General practitioner ☐ Dentist ☐ Pharmacist ☐ Nurse ☐ Mental health worker ☐ Aboriginal health worker

☐ Other (please specify) ....

Privacy Warning: The information contained in this fax message is intended for Quitline Staff only. If you are not the intended recipient you must not copy, distribute, take any action reliant on, or disclose any details of the information in this fax to any other person or organisation.

Patient Information – CONFIDENTIAL

Name: ______________________________ D.O.B __ __/__ __/__ __ __ __

Preferred Phone: (h) ____________ (w) ____________ (m) ____________

Email: ______________________________

Is the patient of Aboriginal or Torres Strait Islander origin?

☐ No ☐ Yes, Aboriginal ☐ Yes, Torres Strait Islander

What is the best time and day for Quitline to call?

Monday-Friday ☐ 9am-1pm ☐ 1pm-5pm ☐ 5pm-8pm

Is it okay for Quitline to leave a message?

☐ Yes ☐ No

Smoking status

☐ Daily ☐ Weekly ☐ Less than weekly Number per day [ ]

What stage is your patient at with quitting?

☐ Not ready (not currently thinking of quitting)
☐ Unsure (thinking about quitting within 6 months)
☐ Ready (planning to quit within 1 month)
☐ Recent quitter (within the last year)

Use of Medication?

☐ Currently using/ planning to use Bupropion Hydrochloride (Zyban®)
☐ Currently using/ planning to use Varenicline (Champix®)
☐ Currently using/ planning to use nicotine patches/ gum/ inhaler/ lozenge/ microtab

What are the patient's health issues relevant to Quitline counsellors?

☐ Heart/lung disease ☐ Respiratory disease ☐ Diabetes ☐ Depression ☐ Anxiety
☐ Psychosis ☐ Pregnancy ☐ Other – please specify ____________

Please note
The interaction of chemicals in cigarettes and some medications e.g. Insulin, some antidepressants / antipsychotics, and the interplay between the chemicals and some symptoms can mean some smokers need monitoring of drug levels and symptoms by their GP through the quitting process.

Health Professional is monitoring the above
☐ Yes
☐ No

I consent to this information being faxed to Quitline and for Quitline Staff to call me at a time that I have suggested on this form. I understand that persons within the organisation with access to the fax machine, who may not be Quitline staff, might view this form. I understand that in Queensland my telephone calls will be recorded for the purposes of quality monitoring and service improvement.

____________ Health Professional Signature

____________ Patient's Signature

__ __/__ __/__ __ Date

For use by Quitline staff

Quitline Confirmation of Action on Referral Date: __ __/__ __/__ __, your referral for ______________________________
has been received by Quitline on __ __/__ __/__ __ , a call back time has been organised for __ __/__ __/__ __.

Referral feedback sent back to ______________________________ (referrer / GP name) on __ __/__ __/__ __.

www.quitnow.info.au
The Quitline is answered 24 hours a day. Counselling is available with hours varying dependent on State or Territory. Specialist staff will call your referred patient back at an agreed time within the next week to provide information, support and advice on smoking cessation.

Resource 6.2: TNT Quit Plan

Name: ____________________ Quit Date: ____________________

Reasons for Quitting

□ Health □ Family □ Money □ Other: ____________________

My QUIT GOAL is: ____________________

____

____

As part of my Quit Plan I will:

□ My support person will be: ____________

□ Tell people I'm quitting

□ Ask others for support

□ Throw away lighters, cigarettes, ashtrays

□ Identify situations where I get strong cravings

□ Plan weekly rewards: ____________

□ Save the money I spend on cigarettes.

Each week I will save $__________

I will use the money to__________

Preparing to Quit

Try this activity at least once or twice during the next week:

When you feel like a smoke, try not to have one, or at least hold off for a few minutes. Observe what happens – notice your craving; how you feel; how long it takes for the craving to pass; take note of what you do to get through the craving.

Quitline

Have the following resources been set up?

□ Quitline fax referral form completed

□ Quit kit supplied

□ QuitCoach signed up for

Identifying triggers and coping strategies

____

____

____

____

____

Nicotine Withdrawal Management

Please select one of the following nicotine withdrawal management plans for the patient to follow based on their Heaviness of Smoking Index score.

	Dependence	Combination Therapy	NRT Dosage
□	High	**Patches** 21mg (24hr)<br>AND<br>**Lozenge or Gum or Inhaler or Microtab**	**Patches:** 21mg/24hr x 1<br>**Lozenge:** 4mg x 6 (maximum)<br>**Gum:** 4mg x 6 (maximum)<br>**Inhaler:** up to 12 cartridges/day<br>**Microtab:** 2 x every 1-2 hrs
□	Moderate	**Patches** 21mg (24hr)<br>AND<br>**Lozenge or Gum or Inhaler or Microtab**	**Patches:** 21mg/24hr x 1<br>**Lozenge:** 4mg x 6 (maximum)<br>**Gum:** 4mg x 6 (maximum)<br>**Inhaler:** up to 12 cartridges/day<br>**Microtab:** 1 x every 1-2 hrs
□	Low	**Patches** 21mg (24hr) or 14mg/16hr<br>AND<br>**Lozenge or Gum or Inhaler or Microtab**	**Patches:** 1 x 21mg/24hr or 14mg/16hr<br>**Lozenge:** 2mg x up to 6/day<br>**Gum:** 2mg x 6 (maximum)<br>**Inhaler:** up to 6 cartridges/day<br>**Microtab:** 1 x every 1-2 hrs

Resource 6.3: CO monitor protocol

Instructions for using CO monitor

1. Confirm that participant consents to completing the CO breath analysis.

2. Explain to the participant that this machine only measures levels of carbon monoxide in the breath and cannot be used to test for other substances (for example use of alcohol or illicit substances).

3. Ask the participant if they are able to comfortably hold their breath for 15 seconds. If they are unsure, reduce the countdown timer to 10 or 5 seconds. If the participant is unable to comfortably hold their breath for 5 seconds do not continue with the CO breath analysis.

4. Using a pair of latex or rubber gloves, attach a new d-piece to the CO monitor unit.

   DO NOT carry out the CO analysis if there are no gloves available.

5. Follow the instructions per Bedfont manual. If the participant is unable to hold their breath for 15 seconds you can alter the countdown timer to go for 10 or 5 seconds. Refer to page 12 of the Bedfont manual.

6. While still wearing the gloves, remove the d-piece and place it in its individual plastic packaging and dispose of the d-piece in a general waste bin. Then dispose of the gloves in a general waste bin.

   NEVER dispose of the d-piece without wearing gloves.

7. Clean hands with Aqium gel after disposing of the d-piece.

Resource 7.1: 5A's Poster

The 5A's

Ask
- Identify and document tobacco use status for every patient at every visit.

Advise
- In a clear, strong, and personalised manner, urge every tobacco user to quit.

Assess
- Assess whether the tobacco user is willing to make a quit attempt at this time.

Assist
- Assist the patient to quit

Arrange
- Arrange follow-up contact

<u>Resource 7.2</u>: Motivational Interviewing Strategies

<u>Motivational Interviewing Strategies</u>

Strategy	How to implement it
Express empathy	Use open-ended questions to explore:<br>• The importance of addressing smoking/tobacco use (*"How important do you think it is for you to quit smoking?"*)<br>• Concerns and benefits of quitting (*"What might happen if you quit?"*)<br><br>Use reflective listening to seek shared understanding<br>• Reflect words or meaning<br>• Summarise<br><br>Normalise feelings and concerns (*"Many people worry about managing without cigarettes"*)<br><br>Support the patient's autonomy and right to choose or reject change (*"I hear you saying you are not ready to quit smoking right now. I'm here to help you when you are ready."*)
Develop discrepancy	Highlight the discrepancy between the patient's present behaviour and their priorities / values / goals (*"It sounds like you are very devoted to your family. How do you think your smoking is affecting your children?"*)<br><br>Reinforce and support "change talk" and "commitment" language<br><br>Build and deepen commitment to change
Roll with resistance	Back off and use reflection when the patient expresses resistance:<br>• "*Sound like you are feeling pressured about your smoking"*<br>Express empathy:<br>• *"You are worried about how you would manage withdrawal symptoms"*<br>Ask permission to provide information:<br>• *"Would you like to hear about some strategies that can help you address that concern when you quit?"*
Support self-efficacy	Help the patient to identify and build on past successes:<br>• *"So you were fairly successful the last time you tried to quit."*<br>Offer options for achievable small steps toward change:<br>• Call the quitline for advice and information<br>• Read about quitting benefits and strategies<br>• Change smoking patterns (e.g. no smoking in home)<br>• Ask the patient to share their ideas about quitting strategies

Resource 7.3: The 5R's – for the smoker unwilling to quit

FOR THE SMOKER UNWILLING TO QUIT: "THE 5 R's"

Smokers may be unwilling to quit due to misinformation, concerned about the effects of quitting, or may be discouraged because of previous unsuccessful quit attempts. These patients may respond to brief motivational interviewing interventions (see Resource 7.2).

After asking about tobacco use, advising the smoker to quit and assessing the willingness to quit, the 5R's motivational interviewing approach should be used.

Strategy	How to Implement
**RELEVANCE**	Encourage the patient to talk about the reasons why quitting smoking is personally relevant to *them*. E.g.<br>• disease status or risk<br>• family or social situation<br>• health concerns<br>• previous quitting experience<br>• personal barriers to cessation
**RISKS**	Ask the patient to identify the negative consequences of continuing smoking – the issues that worry them the most.
**REWARDS**	Ask the patient to identify the benefits they would experience by quitting smoking. E.g.<br>• Improved health<br>• Food will taste better<br>• Improved sense of smell<br>• Saving money<br>• Feeling better about oneself<br>• Home, car, clothing, breath will smell better<br>• Setting a good example for children<br>• Having healthier babies and children<br>• Feeling better physically<br>• Improved appearance: reduced wrinkles/aging skin
**ROADBLOCKS**	Ask the patient to identify barriers to quitting. E.g.<br>• Withdrawal symptoms<br>• Fear of failure<br>• Weight gain<br>• Lack of support<br>• Depression<br>• Enjoyment of smoking<br>• Being around other tobacco users<br>• Limited knowledge of effective treatment options
**REPETITION**	Repeat this process with patients in each session, follow-up with any progress made since the last session.<br><br>Remind patient that most people make repeated quit attempts before they are successful.

Resource 7.4: Stages of Change approach – decision branching tool

STAGES OF CHANGE APPROACH

Looking at the client's stage of readiness to change may also help you in choosing an effective approach to take.[74]

Stage of Readiness	Definition	Suggested Approach
Not ready (pre-contemplation)	Not seriously thinking of quitting in the next 6 months.	**Provide the 5 R's.**<br><br>Show interest and encourage the client to think about the issues.
Unsure (contemplation)	Considering quitting in the next 6 months.	**Provide the 5 R's.**<br><br>Motivate change and offer help to identify and overcome barriers to cessation.
Ready (preparation)	Planning to quit in the next 30 days.	Provide assistance to **develop quit plan**, suggest coping strategies, 4 D's (Delay, Deep breathe, Drink Water, Do something else), encourage social support.
Action	People who have quit.	Congratulate on progress. Check for problems and if present advise or refer appropriately. Offer support and strategies to prevent relapse.
Maintenance	Smokers who've been abstinent for more than 6 months.	Congratulate and reinforce benefits of being a non-smoker.<br>Provide counselling for relapse prevention.
Relapse	Has gone back to smoking.	Reinforce that this is part of the learning experience and not a failure. Encourage and motivate to quit again.

NICOTINE WITHDRAWAL – WHAT IS IT?

When making a quit attempt, a person may experience nicotine withdrawal symptoms. It is important to be aware of these symptoms in order to understand, reassure and remind your client that these will ease (usually after the first 2-4 weeks). Emphasise that urges to smoke may continue, however they are due to stopping smoking, not the use of NRT. Here is a list of possible symptoms and tips for dealing with them:

If this happens…	Try this…
**Irritability, Anxiety, Tenseness**	
As the body adjusts to being without nicotine, feelings of irritability, tenseness and anxiety may be experienced.	Tip: Stress in the first 2 weeks of the quit attempt should be reduced. Ideas – short walks, deep breaths, soak in a bath, meditate.
**Difficulty Concentrating**	
Cigarette cravings may make it harder for a person to concentrate, however concentration levels will return to normal in a few weeks.	Tip: Projects can be broken up into smaller tasks, with regular breaks.
**Restlessness**	
As the body is adjusting to being without nicotine feelings of restlessness may also be experienced.	Tip: Restless energy can be used to get jobs and physical activity done. Caffeine intake should be reduced.
**Insomnia – Problems falling asleep or waking frequently**	
Some people's sleep patterns can be affected from nicotine withdrawal, including problems falling asleep, frequent waking, and strong or unusual dreams.	Tip: Relaxation exercises can be done before bed. Caffeine intake should be reduced. Avoid wearing patches while sleeping.
**Coughing, dry throat and mouth, nasal drip**	
Coughing is a sign that the tar and mucus is being removed from the lungs.	Tip: Drink plenty of water and encourage client to think of the coughing as the lungs cleaning themselves.
**Appetite Changes**	
As nicotine is an appetite suppressant, people making a quit attempt often feel hungrier.	Tip: Keep pre-prepared snacks such as sliced fruit and vegetables on hand. Glucose tablets can be taken for those who develop a sweet-tooth (Caution: people with diabetes should consult their doctor).
**Tingling Sensations and Dizziness**	
As circulation improves, some may experience tingling in fingers and toes, and dizziness.	Tip: Stay calm and sit down and rest until it passes.

NICOTINE REPLACEMENT THERAPY (NRT) USE

What is Nicotine Replacement Therapy (NRT)?

For many smokers it is the urge to smoke at the start that lead to a failed quit attempt. NRT is a way of getting nicotine into the bloodstream without smoking. NRT reduces the symptoms of nicotine withdrawal. NRT acts by providing a 'clean' alternative source of nicotine that the smoker would have otherwise received from tobacco. Nicotine delivered from NRT is absorbed more slowly and generally in a lesser amount than with cigarettes. Examples of NRT include patches, lozenges, gum, oral strips or inhalators.

Is NRT safe?

Yes NRT is safe to use. It only contains the nicotine that would otherwise have been received from cigarettes and not the other harmful constituents of tobacco smoke. It is not the nicotine that causes the health problems associated with smoking but the other things such as tar and carbon monoxide.

Clients with past experiences of NRT:

Given that NRT is now widely available, there is a good chance that smokers may have tried NRT in the past. Some will have found it unhelpful. When using these products without advice smokers may have had unrealistic expectations about how NRT works, may not have liked the initial taste and may not have used it correctly, or for long enough. It is important to encourage clients who have had past negative experiences of NRT to give it another try. You might suggest trying a different type of NRT (i.e. an inhaler instead of a patch) if a client is very hesitant to use.

Talking about NRT with clients:

"NRT is one type of medication available to help you in your quit attempt. It is effective and if used properly, will double your chances of stopping smoking – however it is not a magic cure."

"Medications are an important part of a successful quit attempt, but they are not the only part. Receiving support and advice from a counsellor like me will also roughly double your chances of stopping smoking, but you will need support from other people too. You will also need to make changes to your daily routine and will have to be highly committed to give yourself a good chance of stopping smoking for good."

What types of NRT are available provided as part of the TNT project?

Intervention sites will be provided with the following types of NRT:

Patches | Gum | Inhalator | Oral strips | Cool Drops (lozenge)

How do you use the various types of NRT?

Always inform the clients that they should read the product consumer information that comes with the NRT product.

Nicotine patches are stuck onto the skin and release nicotine into the bloodstream through the outer layer of skin into the blood vessels beneath. Each patch has a special membrane that steadily controls the release of nicotine. Each patch is designed to be worn for 24 hours. If the patch is removed before 24 hours has elapsed the wearer will not receive the full portion of the dose.

It is recommended that a patch is put on just before going to bed and at least 30 minutes after the client has smoked their last cigarette if they continue to smoke. Placing a patch on at night will assist with the cravings the following morning. Wearing a patch may cause sleep disturbances and vivid dreams. Skin irritation beneath the patch occurs in some users. To reduce the risk it is recommended that the site of the patch be changed with each new patch, and that the patch is applied to a clean, dry and hairless area.

Nicotine lozenges are dissolved in the mouth. The lozenge should be placed into the mouth and moved around intermittently. The lozenge should not be chewed, sucked or swallowed whole; just allow it to dissolve. Clients should not eat or drink while using the lozenge. Lozenges should be used whenever there is a strong urge to smoke a cigarette.

Nicotine gum is another oral NRT product**.** Each piece should be chewed slowly to release the nicotine; this will be experienced as a hot peppery taste. The gum should be 'parked' between the cheek and gums so that the nicotine can be absorbed. After a few minutes the gum can be chewed again, and then parked. This should be repeated for 20-30 minutes.

Nicotine inhalers are a cigarette shaped device that delivers nicotine through the mouth. After 20 minutes of continuous use with deep or shallow puffing all of the nicotine will have been used. It is not advised to try to make one cartridge last all day, and although every smoker is different, most successful quitters use around 6 cartridges per day.

Instructions on how to put together the inhaler:

1. Remove the mouthpiece from the plastic wrap.
2. Align the marks on the mouthpiece and pull apart.
3. Take out the blister tray.
4. Peel back to release one cartridge.
5. Press the cartridge firmly into the bottom of the mouthpiece until the seal breaks.
6. Put the top onto the mouthpiece.
7. Again align the marks on the mouthpiece and push the top and bottom firmly together to break the top seal of the cartridge.
8. Twist to misalign the marks.
9. The inhaler is now ready to use.

Nicotine oral strips are thin dissolvable translucent films that are placed on the tongue and pressed against the roof of the mouth until the strip dissolves (approx. 3 mins). At first the client should use one strip every one to two hours. The strips should not be chewed or swallowed.

How much NRT should a client use?
The amount and strength of NRT products used will differ from client to client depending on how heavy their smoking is and their personal preferences. The Heaviness of Smoking Index is a scale that measures, on average, how many cigarettes the client smokes each day and how long after waking they smoke their first cigarette. Depending on their responses clients can be classified as low, moderate or heavy smokers. The Nicotine Protocol provides suggested amounts of NRT depending on this Heaviness of Smoking Index.

DO NOT USE NRT IF:

- The client is allergic to nicotine or any of the other ingredients contained in the product pamphlet.
- The client weighs less than 45kg.

Things to consider

- If the client has Diabetes they will need to monitor their blood sugars more often as their medication requirements for insulin might alter. Encourage the client to discuss this with their General Practitioner (GP).
- Stopping smoking (with or without NRT) can alter the absorption of some medicines, and the dosage of these medicines may need to be changed. Participants should be advised of this. If the client notices an increase in side effects related to medications they should consult their GP or other health professional.
- Medications that can be affected when someone reduces or quits smoking include Warfarin, Benzodiazepines e.g. Valium, Xanax, Chlorpramazine (Largactil), Theophyllinne (used for asthma and other respiratory conditions), some beta blockers, Clozapine, Olanzapine, Haloperidol and Insulin. If the client is taking any of these medications, they should consult their doctor (before quitting smoking/using NRT).

Resource 7.7: NRT Protocol

Low Dependence	Moderate Dependence	High Dependence
1 x (14mg **OR** 21mg) patch<br>**OR**<br>Up to 6 x gum **OR** 6 x 2mg lozenge per day **OR** up to 6 inhaler cartridges per day **OR** 1 oral strip every 1 to 2 hours<br>**TIME: DAILY FOR <u>2 WEEKS</u>**	1 x 21 mg patch<br>**+**<br>Up to 6 x 4mg gum **OR** 6 x 4mg lozenge per day **OR** 6 to 12 inhaler cartridges per day **OR** 1 oral strip every 1 to 2 hours<br>**TIME: DAILY FOR <u>2 WEEKS</u>**	1 x 21 mg patch<br>**+**<br>Up to 6 x 4mg gum **OR** 6 x 4mg lozenge per day **OR** up to 12 cartridges per day **OR** 2 oral strips every 1 to 2 hours<br>**TIME: DAILY FOR <u>2 WEEKS</u>**
1 x (14mg **OR** 21mg) patch<br>+<br>***Reduce gum/lozenge/inhaler / oral strip use***<br>**TIME: DAILY FOR <u>2 WEEKS</u>**	1 x 21 mg patch (24hr)<br>+<br>***Reduce gum/lozenge/inhaler / oral strip use***<br>**TIME: DAILY FOR <u>2 WEEKS</u>**	1 x 21 mg patch (24hr)<br>+<br>***Reduce gum/lozenge/inhaler / oral strip use***<br>**TIME: DAILY FOR <u>2 WEEKS</u>**
1 x (14mg **OR** 21mg) patch<br>**TIME: DAILY FOR <u>3 – 4 WEEKS</u>**	1 x 21 mg patch<br>**TIME: DAILY FOR <u>3 – 4 WEEKS</u>**	1 x 21 mg patch<br>**TIME: DAILY FOR <u>3 – 4 WEEKS</u>**

Resource 7.8: What if the NRT is not working?

WHAT IF THE NRT ISN'T WORKING?

Client's Issue	Potential problem solvers
The patch I was wearing has given me a rash.	Always apply a new patch on a new part of the body that should be clean, hairless, and dry.
I'm having trouble sleeping/vivid dreams.	Remove the patch while sleeping.
I feel nauseous.	Consider using a different form of NRT e.g. if the client is using lozenges then try the inhaler instead.
The NRT isn't working – I'm still craving cigarettes and I'm still smoking.	Consider adding another form of NRT e.g. wearing a patch and chewing gum or having a lozenge when strong cravings occur.<br><br>Make sure the client is using the NRT product correctly – consult the product information pamphlet.
I don't like the NRT product I'm using because it doesn't taste good, it's too hard to use, I can't use it when I want to (for example chewing gum at work) or it's just not for me.	Consider using a different form of NRT e.g. if the client is using lozenges then try the inhaler instead.<br><br>Make sure the client is using the NRT product correctly – ask them to consult the product information pamphlet or go through it with them.<br><br>Talk to their GP or other health professional about other medication and strategies to quit smoking.

Resource 7.9: Relapse and Coping Strategies

RELAPSE AND COPING STRATEGIES

Identify high risk relapse situations, for example:

- Arguments with partners or family
- Work/financial pressures
- Christmas
- Grief
- When drinking alcohol
- On holidays
- In the company of smokers in a place where the client normally used to smoke

Pro-active strategies:

- Relapse back onto NRT instead of cigarettes – encourage patients to keep using NRT
- Develop plans to avoid and deal with the situations identified above
- Remember the reasons for quitting smoking
- Adopt active strategies, i.e. instead of a cigarette – go for a walk, read a book etc.
- Try to avoid major triggers for smoking early in the quit attempt, i.e. alcohol, coffee, smoking friends.

Coping strategies: The 4 D's

Delay	• Delay acting on the urge to smoke. After 5mins the urge to smoke weakens and resolve to quit comes back
Deep Breathing	• Take a long slow breath in and slowly release it out again. Repeat 3 times.
Drink Water	• Drink water, slowly holding it in the mouth a little longer to savour the taste.
Do Something	• Do something else to take your mind off smoking. E.g. exercise is a good alternative.

Resource 7.10: NRT log

Date	Staff Name	NRT Type<br><br>(e.g. patch, inhaler)	NRT strength<br><br>(e.g. 21mg/ gum 4mg)	Amount Provided<br><br>(e.g. 1 box patches/14 pieces gum)

Resource 8.1: Letter to GP

Dear ____________________

This letter is to inform you that Mr / Mrs /Ms____________________________ is interested in quitting smoking.

As you are involved in this patient’s physical and mental health care, we wanted to notify you or their desire to quit smoking. They would like to talk to you about their smoking and different ways to go about quitting and becoming smoke free.

We look forward to your support in helping this patient achieve their goal.

Yours sincerely,

____
