# Supplementary material for: An organisational change intervention for increasing the delivery of smoking cessation support in addiction treatment centres: study protocol for a randomized controlled trial
Source: Trials. 2016 Jun 14;17:290. doi: 10.1186/s13063-016-1401-6 (PMC4907075; doi:10.1186/s13063-016-1401-6)
Supplement: Additional file 3: — TNT Intervention Manual - BRIEF. TNT Project Working Manual. A brief working document containing 1–2 page user-friendly summaries of each of the eight components of the TNT intervention with links to resources developed and/or provided as part of the trial. (PDF 2612 kb) [file 13063_2016_1401_MOESM3_ESM.pdf]

TNT Project Working Manual

This is a collaborative project between

Which is funded by

NHMRC and the Cancer Council NSW

Toll Free Number
1800 993 063

E: TNT@newcastle.edu.au

Postal Address
CTNMH
PO Box 833
Newcastle NSW 2300

TNT STUDY MANAGEMENT & ADVISORY TEAM

**Associate Professor Billie Bonevski – Chief Investigator**<br>School of Medicine & Public Health<br>University of Newcastle<br>P: (02) 4033 5170<br>billie.bonevksi@newcastle.edu.au	
**Prof. Anthony Shakeshaft**<br>National Drug & Alcohol Research Centre<br>University of New South Wales<br>a.shakeshaft@unsw.edu.au	**Prof. Michael Farrell**<br>National Drug & Alcohol Research Centre<br>University of New South Wales<br>michael.farrell@unsw.edu.au
**Dr. Flora Tzelepis**<br>School of Medicine & Public Health<br>University of Newcastle<br>flora.tzelepis@newcastle.edu.au	**Mr. Scott Walsberger**<br>Tobacco Control Unit<br>Cancer Council NSW<br>scottw@nswcc.org.au
**Prof. Catherine d'Este**<br>National Centre for Epidemiology and Population Health (NCEPH)<br>The Australian National University<br>catherine.deste@anu.edu.au	**A/Prof. Christine Paul**<br>School of Medicine & Public Health<br>University of Newcastle<br>chris.paul@newcastle.edu.au
**Dr. Jamie Bryant**<br>School of Medicine & Public Health<br>University of Newcastle<br>jamie.bryant@newcastle.edu.au	**Dr. Peter Kelly**<br>School of Psychology<br>University of Wollongong<br>pkelly@uow.edu.au
**A/Prof. Andrew Searles**<br>Hunter Medical Research Institute &<br>University of Newcastle<br>andrew.searles@newcastle.edu.au	**A/Prof. Adrian Dunlop**<br>Drug and Alcohol Services<br>Hunter New England Lower Health District<br>adrian.dunlop@hnehealth.nsw.gov.au
**Ms Rae Fry**<br>Tobacco Control Unit<br>Cancer Council NSW<br>raef@nswcc.org.au	**A/Prof. Judith Prochaska**<br>Stanford Prevention Research Centre<br>Stanford University<br>jprochaska@stanford.edu
**Dr. Ashleigh Guillaumier – Project Manager**<br>School of Medicine & Public Health<br>University of Newcastle<br>P: (02) 4033 5718<br>ashleigh.guillaumier@newcastle.edu.au	**Eliza Skelton – PhD Student**<br>School of Medicine & Public Health<br>University of Newcastle<br>P: (02) 4033 5711<br>eliza.skelton@newcastle.edu.au

Table of Contents

Components of the TNT intervention 5

1. Engage Organisational Support 6
2. Identify and Support a Champion 8
3. Promote Centre Policies that Support and Provide Tobacco Dependence Services 10
4. Implement a System of Identifying Smokers 12
5. Provide Education and Resources 14
6. Provide Case-Worker and Client Feedback 16
7. Include Evidence-Based Tobacco Dependence Treatments 18
8. Maintenance and Follow-Up 23

TNT Resource Kit 25

Resource 1.1: CCNSW Tackling Tobacco Pamphlet – “Incorporating smoking cessation into drug and alcohol treatment – Information for staff” 26

Resource 1.2: Agenda template for TNT briefing staff meeting 27

Resource 1.3: Newsletter article templates for TNT 28

Resource 1.4: Reminder email or flyer prompts for changes to tobacco polices 29

Resource 2.1: Support Champion “Identifier” Poster 30

Resource 3.1: CCNSW “Tackling Tobacco Policy Toolkit” 31

Resource 4.1: Desktop reminder to assess smoking status 32

Resource 5.1: CCNSW Tackling Tobacco pamphlet – “Not ready to give up” 33

Resource 5.2: CCNSW Tackling Tobacco pamphlet – “Thinking about giving up” 34

Resource 5.3: CCNSW Tackling Tobacco pamphlet – “Ready to give up” 35

Resource 5.4: CCNSW Tackling Tobacco pamphlet – “Staying a non-smoker” 36

Resource 5.5: Quit kits and ordering information 37

Resource 6.1: Quitline Fax Referral Form 38

Resource 6.2: TNT Quit Plan 39

Resource 6.3: CO monitor protocol 40

Resource 7.1: 5A’s Poster 41

Resource 7.2: Motivational Interviewing Strategies 42

Resource 7.3: The 5R’s – for the smoker unwilling to quit 43

Resource 7.4: Stages of Change approach – decision branching tool 44

Resource 7.5: Nicotine Withdrawal – What is it? 45

Resource 7.6: Nicotine Replacement Therapy – General guide 46

Resource 7.7: NRT Protocol 49

Resource 7.8: What if the NRT is not working? 50

Resource 7.9: Relapse and Coping Strategies 51

Resource 7.10: NRT log 52

Resource 8.1: Letter to GP 53

TACKLING NICOTINE TOGETHER: STUDY OVERVIEW

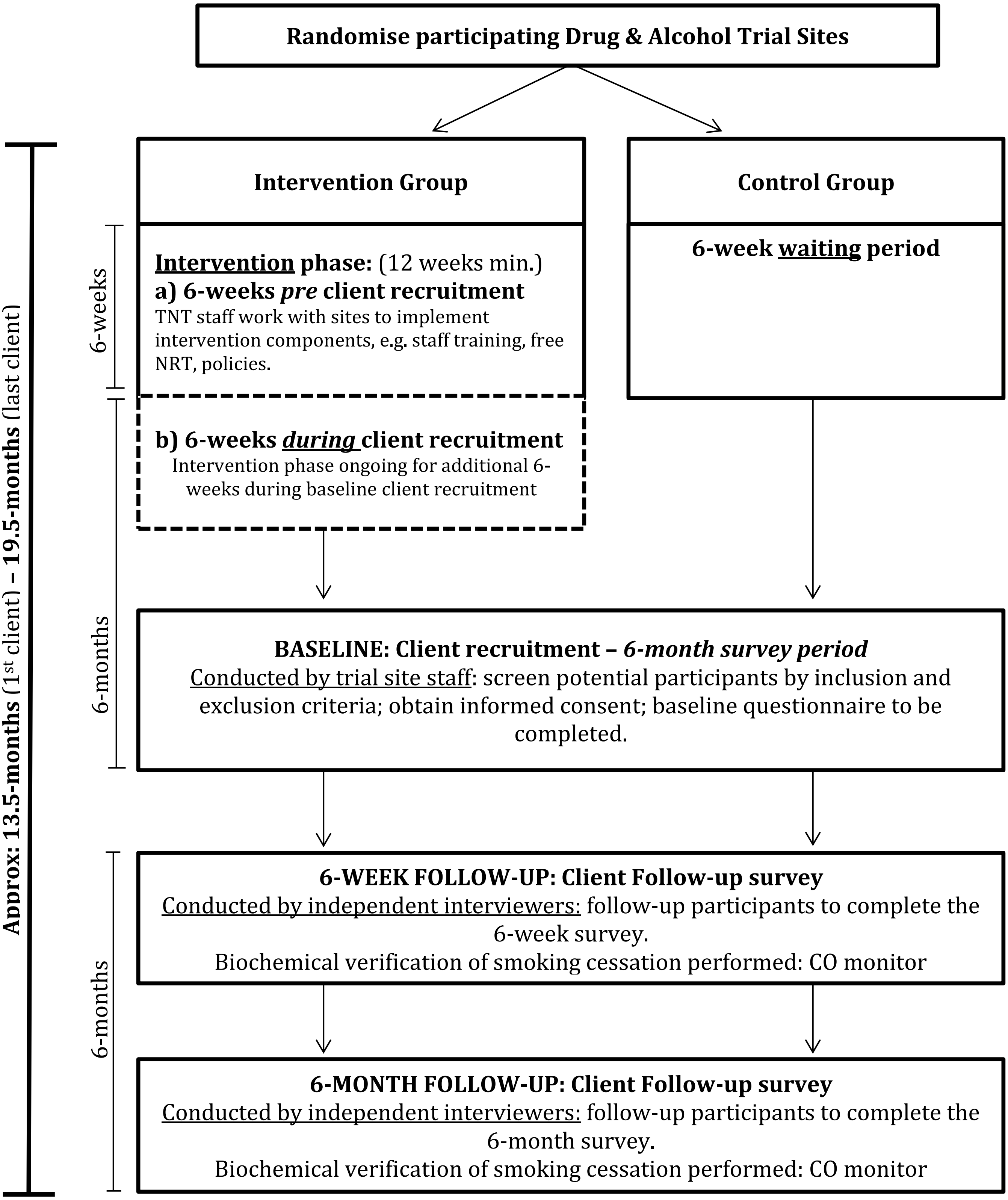

Components of the TNT intervention

The resulting TNT organisational change intervention is devised of eight core components:

1. Engage Organisational Support
2. Identify and support a champion
3. Promote centre policies that support and provide tobacco dependence services
4. Implement a system of identifying smokers
5. Provide education and resources
6. Provide case-worker and client feedback
7. Include evidence-based tobacco dependence treatments
8. Maintenance and follow-up

These eight organisational change components can be each conceptualised as pieces of a puzzle that when combined are the Tackling Nicotine Together intervention (see figure 1).

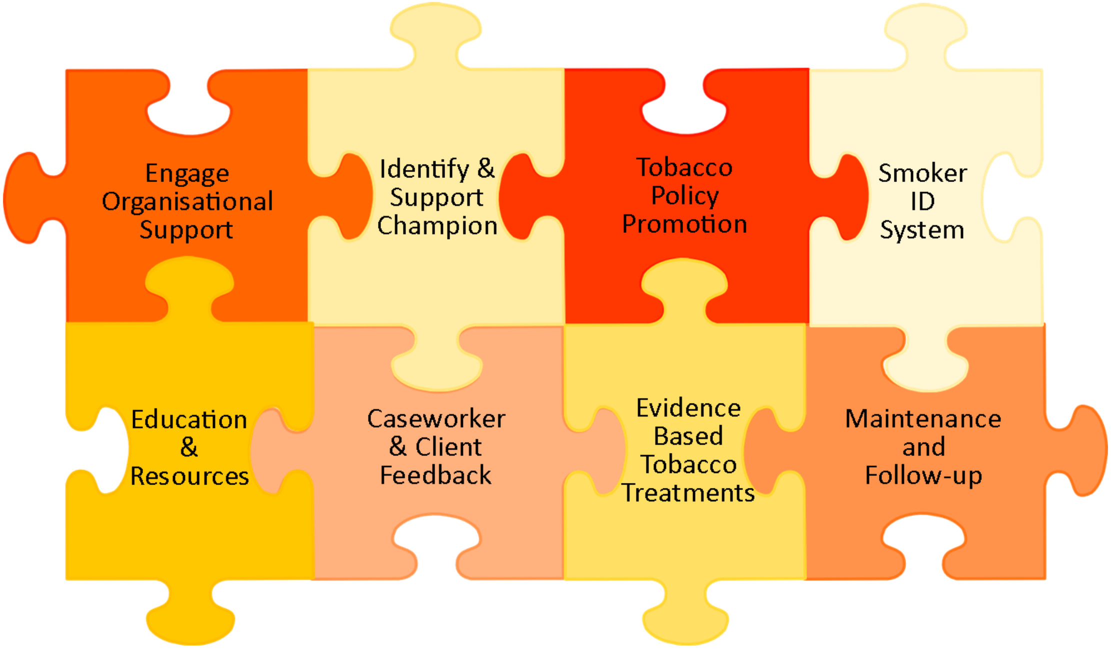

Figure 1. TNT organisational change intervention components

The TNT intervention is flexible and can be tailored to the needs of each of the individual drug and alcohol services participating in the study.

1. Engage Organisational Support

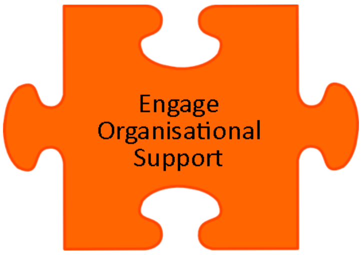

Goal: To engage the support and commitment from management and staff throughout the project period (pre intervention, during implementation and post intervention) in order to increase the likelihood of adoption and maintenance of the Tackling Nicotine Together intervention.

MAIN STEPS TO IMPLEMENTATION OF THIS STRATEGY

A number of strategies can be used to engage and demonstrate organisational support for the TNT intervention.

The following is an excerpt from the Queensland Government's "*Change Management Best Practices Guide*" and lists the fundamentals of change communication:

- Clearly communicate the change vision and do it early
- Outline the benefits and impacts of the change
- Ensure the organisations leaders are actively communicated with throughout the change process
- Use multiple channels to communicate the change message
- Provide opportunities for dialogue
- Repeat change messages often
- Monitor and measure the effectiveness of communications

1.1. *Advocacy*

Build a case for change

Information regarding the need for addressing smoking cessation in the context of drug and alcohol treatment will be provided to management and staff (see Resource 1.1).

1.2. *Staff Meetings*

Initial staff briefing

Prior to the start of the intervention period, a staff meeting should be held to inform all staff in the service of the project and to discuss the best ways to maximise engagement with the intervention program.

Standing meeting agenda item

The smoking cessation intervention program could be added as a standing agenda item for staff meetings to provide an on-going forum for discussion of successes and challenges in implementing the change in practice as part of routine care (see Resource 1.2).

1.3. ***Organisation-wide communications***

To promote on-going awareness of the change in policies and practices involved with the TNT intervention, make use of a number of communication strategies. Methods of message communication may include:

- Articles in existing organisation newsletters (see Resource 1.3)
- Brief reminders about new tobacco policies and practices – these prompts can take the form of emails or flyers (see Resource 1.4)

**RESOURCES FOR THIS STRATEGY**	
Resource 1.1	CCNSW Tackling Tobacco pamphlet "Incorporating smoking cessation into drug and alcohol treatment – Information for staff"
Resource 1.2	Agenda template for TNT briefing staff meeting
Resource 1.3	Example newsletter articles
Resource 1.4	Reminder email or flyer prompts

2. Identify and Support a Champion

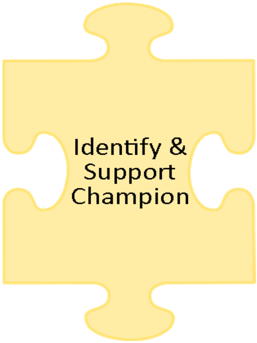

Goal: To identify members of staff from each organization to be appointed the role of support champion who will facilitate the adoption of the organisational change intervention components. The aim is to identify and support staff that will champion the introduction and/or improvement in delivery of smoking cessation care as part of usual care.

MAIN STEPS TO IMPLEMENTATION OF THIS STRATEGY

2.1. *Select a Champion*

Support champions can be selected through one of two ways:

Formal: careful and thoughtful selection process of well-defined eligibility criteria created by managerial staff that individuals must satisfy (e.g. degree in health-related field; communication skills; current opinion leader; charismatic)

Informal: individuals are seen to naturally fulfil the role in their normal work capacity

The support person will be formally recognised at the organisation as the TNT champion. In order to notify all staff of the individual who has fulfilled the role as the support champion a poster will be placed in the staff room of the organisation. The poster will have the name and picture of the support champion as well as a brief outline of their role and how they can help staff and clients (*see Resource 2.1*).

2.2. *Training a Champion*

The support champion is encouraged to attend the staff smoking cessation training provided as part of the TNT intervention (see *section 5. Provide Education and Resources*). The one-day training session will cover:

- How to address smoking in the AOD setting (with a focus on brief advice)
- Motivational interviewing techniques
- Cessation counselling
- How to administer nicotine replacement therapy

2.3. ***Role of a Champion***

<u>Advocate for change</u>

The champion will advocate changes to organisation policies and practices that support the provision of smoking cessation support as part of routine care.

<u>Primary contact for the research team</u>

The champion will be the main point of contact between the research team and the organisation. This relationship will serve as the primary method for dissemination of project materials and key intervention messages. This line of communication will also be open for champions to troubleshoot issues or refer on any concerns relating to the research project.

<u>Maintain the TNT intervention as a priority</u>

The champion will work to maintain the TNT project as a priority among staff and will lead the project activity at their organisation.

The champion will also be responsible for TNT project-related equipment, such as survey data collection devices, paper-based information statements, equipment logs, etc.

<u>Support staff development and troubleshooting</u>

The champion will support other staff members to deliver smoking cessation care as part of routine care and will work to positively implement policy level and environmental changes to the workplace. The champion might achieve this by prompting discussions within the organisation and among staff to identify the barriers preventing them from achieving their goal.

**RESOURCES FOR THIS STRATEGY**	
Resource 2.1	Support Champion “Identifier” Poster
TNT Monthly Newsletter	A monthly newsletter will be provided to the support champion to distribute to staff of the organisation. The newsletter will be created by the research team with the assistance of the support champion. The newsletter will contain information concerning the project objectives, current goals, resources available to staff/clients, upcoming training and ways to contact the support champion about issues/ concerns
Touchscreen Tablet (survey data collection tool)	A touchscreen tablet device will be allocated to each organisation. This device will be used to conduct surveys with clients. A safe lock box for the device will be provided as well as instructions on how to secure the device. The support champion will be responsible for the device and must ensure that it will not be at risk of theft.

3. Promote Centre Policies that Support and Provide Tobacco Dependence Services

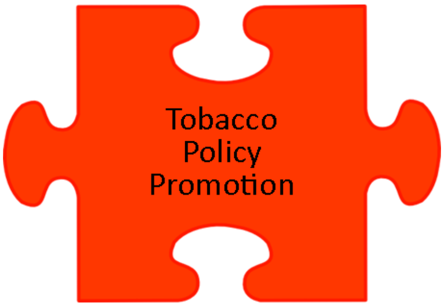

Goal: Develop organisation-specific tobacco policies, and to ensure that all staff and clients of the organisation are aware of the policies and understand what they include.

MAIN STEPS TO IMPLEMENTATION OF THIS STRATEGY

Project staff will assist with implementing smoke-free policies, smoke-free signage, support for staff to quit and changes to processes to create a cessation-supportive environment. Services that have policies in place will be assisted in developing programs to maximize enforcement.

The implementation of this strategy will be largely dependent on the current policies and practices of the organization. All services will be assisted in developing programs to maximize awareness of the service tobacco policy and enforcement strategies.

3.1. *Policy Development*

Policy needs to consist of a statement, policy aims, policy components and a plan for implementation.

Services that do not currently have a smoke-free policy will be encouraged to work through the CCNSW Policy Toolkit (see Resource 3.1) to develop their own policies.

3.2. *Policy Promotion*

The physical environment of the organisation can be restructured in order to be more supportive of the policy changes:

- Restrict access to areas that were once used for smoking or transform them into areas that have a different purpose
- Adding signage to the environment to help ensure non-smoking behaviour is maintained

3.3. *Policy Enforcement*

Rewarding non-smoking

- Provide positive feedback to staff and clients for adhering to the new policy
- Arrange rewards to celebrate any policy successes

Pre-warning of the consequences of smoking

- Decide on appropriate consequences of breaking the policy (e.g. written warning systems, removal of reward)
- Advise clients and staff of penalties for not adhering to the policy

Punishment for not abiding by the organisations policy

The severity of the punishment for performing the prohibited behaviour of smoking at the organisation will depend on what the centre staff and management believe is appropriate. The appropriate punishment can be decided upon after the behaviour has been performed. It can be openly stated that there will be consequences but the exact consequence does not need to be publicly stated or known.

RESOURCES FOR THIS STRATEGY	
Resource 3.1	CCNSW "Tackling Tobacco Policy Toolkit"

4. Implement a System of Identifying Smokers

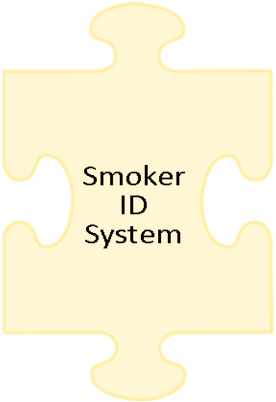

Goal: Develop an organisation-wide system to ensure that all patients are asked about tobacco use as part of every clinical encounter. Such a system would be implemented with the intention that clinicians and care-providers have more opportunity to offer and provide smoking-related care.

MAIN STEPS TO IMPLEMENTATION OF THIS STRATEGY

4.1. *Information to assess and record*

In the initial assessment ask about:

- If they are a smoker
- If they had wanted this visit to provide them with a quit attempt
- If they would like support and information regarding quitting

4.2. *Decide on a service-appropriate method of assessment*

Treatment centres will develop a smoke identification system that is appropriate to their current operations to ensure that all clients are asked about their tobacco use at every clinical encounter. The first step is identifying the methods that work best for the service.

Smoking Status Assessment Options:

<u>Updating current intake / initial assessment forms</u>
If the service does not currently assess tobacco use at intake or initial assessment, include the recording of tobacco use on existing intake and/or assessment forms. For example:

**Tobacco use:**	**Current**	**Former**	**Never**	*(circle one)*

If tobacco use is already recorded as part of existing assessment:

- Move item for assessing smoking status closer to beginning of assessment to increase chances of it being asked and documented

Tobacco Use stickers
Where client files are paper-based a sticker system may be employed. Stickers are to be placed on all client files, which provide a quick and easy visual marker to signify that client is a current tobacco smoker.

Electronic Prompt
In services that use electronic systems to store patient files and document progress, automated messages to appear on screen when a new file is created on a patient may be programmed. Reminders may also be set to track progress in follow-up consultations.

Subsequent Assessment Options to ensure tobacco-use is obtained:
Set a note in client file for follow up discussion surrounding smoking once they have identified as a smoker. This note can be on the paper or electronic patient files.

- Record in clinical assessment or daily progress notes of client NRT use (include type and dosage).
- Repeat assessment will not be necessary in the case of a client who has never used tobacco or not used tobacco for many years.

4.3. *Clarify staff responsibility for implementation*

All staff members that are responsible for gathering initial information from clients, recording daily habits and subsequent follow up documentation should be informed of the importance of this activity.

4.4. *Ways to increase awareness of smoker identification system*

During the period of implementation of a smoker identification system, it may be helpful to use written, visual and verbal reminders prompting staff to assess and document patient smoking status. Prompts and reminders may vary depending on the systems of documentation each service uses. Examples may include desk reminders, posters or organisation wide emails or memos.

- Desk prompts (see Resource 4.1)
- Brief flyer or email reminders (see Resource 1.4)

RESOURCES FOR THIS STRATEGY	
Resource 4.1	Desktop reminder to assess smoking status
Resource 4.2	Poster / email reminder to assess smoking status

5. Provide Education and Resources

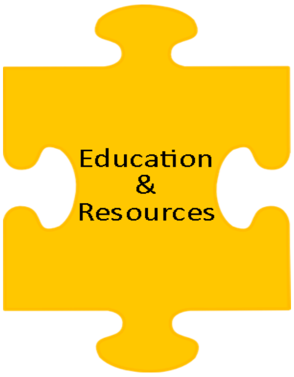

Goal: To provide education, training and resources necessary to ensure that staff have the skills and information to assist their clients in making quit attempts.

MAIN STEPS TO IMPLEMENTATION OF THIS STRATEGY

5.1. *Staff Training*

Staff members are encouraged to attend training on addressing client smoking and the delivery of smoking cessation care. Training will be provided by accredited trainers with content based on the CCNSW's Tackling Tobacco training program.

Aims of training:

- Increase knowledge and understanding of key issues in addressing tobacco use for people in AOD treatment
- This training will cover how to address smoking in the drug and alcohol setting, motivational interviewing techniques, cessation counselling and how to administer nicotine replacement therapy.
- The training focuses on brief advice – the '5As' (Ask, Assess, Advise, Assist, and Arrange Follow-up) and appropriate NRT provision

Training conduct:

- One-day smoking cessation workshops will be held in each geographic region for service staff to attend.

Trainer assessment

- Trainer knowledge will be assessed by completion of the National Centre for Smoking Cessation and Training knowledge test

5.2. *Provision of Resources*

The research team will provide educational material and resources to support implementation of the TNT intervention (contained in the TNT Resource Kit section of the manual). A number of self-help booklets and educational information flyers developed as part of the Cancer Council NSW 'Tackling Tobacco' Program will be provided. Intervention treatment centres will be routinely contacted by the research team to replenish intervention resources during the study period.

RESOURCES FOR THIS STRATEGY	
Resource 1.1	CCNSW Tackling Tobacco pamphlet “Incorporating smoking cessation into drug and alcohol treatment – Information for staff”
Resource 5.1	CCNSW Tackling Tobacco pamphlet “*Not ready to give up*”
Resource 5.2	CCNSW Tackling Tobacco pamphlet “*Thinking about giving up*”
Resource 5.3	CCNSW Tackling Tobacco pamphlet “*Ready to give up*”
Resource 5.4	CCNSW Tackling Tobacco pamphlet “*Staying a non-smoker*”
Resource 5.5	Quit kits and ordering information

6. Provide Case-Worker and Client Feedback

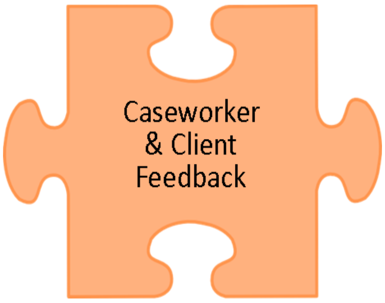

Goal: To keep the service, staff and clients motivated and engaged with the organisational change effort surrounding tobacco policy and smoking cessation care and support.

MAIN STEPS TO IMPLEMENTATION OF THIS STRATEGY

6.1. *Service: Staff Survey Findings*

Prior to the randomisation process where in services were allocated to the intervention or control groups, all participating service staff completed an online survey about current tobacco policies and practices. Services will be fed back aggregated data concerning:

- staff and manager attitudes to the provision of smoking care
- perceptions of current practice regarding smoking and tobacco use
- perceptions of barriers to implementing changes to smoking policy and provision of care

6.2. *Service: Intervention newsletter*

An intervention group newsletter that includes feedback on performance will be circulated by the research team to all intervention services.

6.3. *Service: Benchmarking*

By collating recruitment performance indicators for the top performing 10% of services, a benchmark of what recruitment goals can be achieved in a given period will be promoted to services. These benchmark figures will be specific to the size & type of the service (particularly government vs. non-government) and will be circulated via email attachment to the key contact at each site.

6.4. *Service: Quitline referrals*

Services will be asked to note their service name on any Quitline fax referral forms that are used throughout the project (see Resource 6.1 – Quitline Fax Referral Form). The number of Quitline referrals made by each service can then be tracked in coordination with Quitline research staff. This information will be provided as feedback to service sites.

6.5. ***Service: Reminders***

Reminders about the activities required as part of the research project, as well as reminders of content presented in staff training can be circulated to service staff via emails, newsletters and desk top visual prompts. Content can include:

- Quick tips on providing brief advice to quit
- Reminders to record client smoking status in case notes
- Prompts to provide quit smoking pamphlets
- Reminders to use Quitline fax referral forms

6.6. ***Client: Recording smoking status***

As part of the intervention, services are being encouraged to assess and record smoking status at all clinical encounters. Service providers can use smoking notes in a client's case files to prompt asking the question about smoking and following up on whether the client requires any further information or support about their smoking and interest in quitting.

6.7. ***Use of quit plans***

Services will be encouraged to complete quit plans with their clients and refer back to these quit plans throughout the period of the client's engagement with the service. The quit plan can be used as a progress feedback tool with clients (see Resource 6.2 – Quit Plan).

6.8. ***CO monitors***

Carbon monoxide (CO) monitors will be used to verify smoking status at the 6-month follow-up. However, many services have their own CO monitors; where this is the case, services will be encouraged to use these to provide motivational feedback to clients regarding their progress during quit attempts (see Resource 6.3 – CO monitor protocol).

RESOURCES FOR THIS STRATEGY	
Resource 6.1	Quitline Fax Referral Form
Resource 6.2	TNT Quit Plan
Resource 6.3	CO monitor protocol (including instructions for use)

7. Include Evidence-Based Tobacco Dependence Treatments

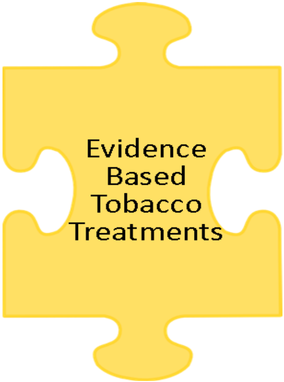

Goal: To ensure that service clients who are smokers have access to effective, evidence-based behavioural support and medication to aid in cessation.

MAIN STEPS TO IMPLEMENTATION OF THIS STRATEGY

Staff will receive training on the delivery of evidence-based tobacco dependence treatment (see Section 5: Provide Education and Resources). Resources such as QUIT Pack, QUITline and the My QuitBuddy app will be made use of where appropriate. Nicotine Replacement Therapy (NRT) will be provided free of cost during treatment.

This section of the manual provides a brief overview of these treatments, as well as useful resources covering delivery, use and troubleshooting for the implementation of smoking cessation as part of routine client care.

7.1. *Brief Advice – The 5A's*

Brief advice is a standardized procedure involving brief advice to quit smoking with brief counselling about methods recommended for smokers in primary care settings. The approach requires relatively little training, assessment or time, and follows a format of assessing smoking, advising the person to quit, providing assistance with quitting and conducting follow-up or booster sessions.

The following table provides an overview of how to implement each of the 5A's, with links to further resources. The strategy table has been adapted from *"Treating Tobacco Use & Dependence: Clinical Practice Guideline 2008 Update"* US Department of Health and Human Services.

A1. ASK

Systematically identify all tobacco users at every visit

Strategies for Implementation	Resources
**Implement a service-wide system that ensures that, for *every* patient at *every* clinic visit, tobacco use status is queried and documented.**	Section 4: Implement a System of Identifying Smokers

A2. ADVISE

In a *clear, strong and personalised* manner, urge every tobacco user to quit

Strategies for Implementation	Resources
**Clear advice:**<br>"*It is important that you quit smoking now, and I can help you*"<br>"*Cutting down while you are ill is not enough*"<br>"*Occasional or light smoking is still dangerous*"<br><br>**Strong advice:**<br>"*The best thing you can do for your health is to stop smoking, and I would advise you to stop as soon as possible*"<br><br>**Personalised advice:**<br>Tie tobacco use to current situation, for example current symptoms and health concerns; social and economic costs; impact of tobacco use on children and others in the household.	**Resource 7.1:** 5A's poster

A.3 ASSESS

Determine every tobacco user's willingness to make a quit attempt at the time.

Strategies for Implementation	Resources
"Are you willing to give quitting a try?"<br><br>**YES: provide assistance (**see – A4. ASSIST**)**<br><br>**NO: provide motivational interviewing**	**Resource 7.2:** Motivational Interviewing Strategies<br><br>**Resource 7.3:** 5R's – the smoker unwilling to quit

A4. ASSIST

Aid the patient in quitting by providing counselling and medication

Strategies for Implementation	Resources
Looking at the patient's **readiness to change** may help in choosing an effective approach to take.	**Resource 7.4:** Stages of Change Approach – decision branching tool
Help the patient with a **QUIT PLAN**.<br><br>A patient's **preparations for quitting** may include:<br>Set a date ideally within 2 weeks<br>Tell others about quit plans<br>Anticipate challenges e.g. nicotine withdrawal symptoms<br>Remove tobacco products from your environment	**Resource 6.2:** Quit Plan<br><br>**Resource 7.5:** Nicotine Withdrawal Symptoms
**NICOTINE REPLACEMENT THERAPY**<br>Explain how these medications increase quitting success and reduce withdrawal symptoms.	**Resource 7.6:** NRT Use<br>**Resource 7.7:** NRT Protocol<br>**Resource 7.8:** What if the NRT isn't working?
Provide practical **COUNSELLING** (problem solving / training):<br>*Abstinence*. Striving for total abstinence is essential. Not even a single puff after the quit date.<br>*Past quit experience*. Identifying what helped/hurt in previous quit attempts and build on past success.<br>*Anticipate triggers or challenges.* Discuss how the patient will overcome these (e.g. avoid triggers, alter routines).<br>*Alcohol.* Alcohol is associated with relapse and should be avoided while quitting.<br>*Others in the household.* Quitting is more difficult when there is another smoker in the household. Patients should encourage housemates to quit with them or to not smoke in their presence.	**Resource 7.9:** Relapse & Coping Strategies<br>**Resource 7.5**: Nicotine Withdrawal Symptoms
**EXISTING QUIT RESOURCES**<br>These resources will be provided to services by the TNT team.	**Resource 5.5:** Quit kit<br>**Resources 1.1, 5.1, 5.2, 5.3, 5.4**: CCNSW Tackling Tobacco pamphlets<br>**Resource 5.5**: Quitline information

A5. ARRANGE

Ensure follow-up contacts, either in person or via telephone.

Strategies for Implementation	Resources
**Clients who <u>do</u> have on-going contact with the service:**<br>*Timing*: follow-up soon after the quit date, preferably in the first week.<br><br>*Actions during follow-up contact*:<br>Identify challenges in the immediate future.<br>Assess medication use and problems.<br>Remind patients of quitline support.<br>Address tobacco use at next clinical visit (treat tobacco use as a chronic disease).<br>For those patients who are abstinent, congratulate them on their success.<br>If tobacco use has occurred, review circumstance and elicit recommitment to total abstinence.	**Resource 6.2:** Quit Plan<br>**Resource 7.9:** Relapse & Coping Strategies<br>**Resource 7.5:** Nicotine Withdrawal Symptoms<br>**Resource 7.7:** NRT Protocol<br>**Resource 7.8:** What if the NRT isn't working?
**Clients who <u>do not</u> have on-going contact with the service:**<br>Arrange for follow-up contact with other existing quit services, such as Quitline, by using the Quitline Fax Referral form. If the client has contact with a GP, you might also consider sending a letter to their GP to inform them of quit interest, attempts and need for further follow-up.	**Resource 6.1:** Quitline Fax Referral Form<br>**Resource 8.1:** GP Letter

7.2. *Use of existing QUIT resources*

Resources such as QUIT Pack, QUITline and the My QuitBuddy app will be made use of where appropriate.

- National Quitline number: 13 QUIT (1378 48) – Quit Kits can also be ordered from here
- My QuitBuddy: personalised, interactive app, free to download on iPhone, iPad and Android

7.3. *Provision of Nicotine Replacement Therapy (NRT)*

Nicotine Replacement Therapy (NRT) will be provided free of cost during treatment. This intervention will provide a flexible range of NRT options. An NRT log will be provided to track usage of the NRT provided to the service (see Resource 7.10 – NRT log). NRT can be in the form of a gum, patch, nasal spray, inhaler and a lozenge. These are first line therapy, and varenicline or bupropion (prescription only; not provided as part of TNT) can be used along or as an adjunct to NRT. Many smokers are unaware of these effective cessation methods and most underestimate their benefit.

Staff and patients are to be provided with access to and encouragement in using medications and nicotine replacement therapies that will enable them to quit smoking. Individuals that are using these supports already should be further encouraged to adhere to the treatment in order to maintain their quit attempt as it will increase their likelihood of successfully quitting.

**RESOURCES FOR THIS STRATEGY**	
Resource 1.1	CCNSW Tackling Tobacco pamphlet "*Incorporating smoking cessation into drug and alcohol treatment – Information for staff*"
Resource 5.1	CCNSW Tackling Tobacco pamphlet "*Not ready to give up*"
Resource 5.2	CCNSW Tackling Tobacco pamphlet "*Thinking about giving up*"
Resource 5.3	CCNSW Tackling Tobacco pamphlet "*Ready to give up*"
Resource 5.4	CCNSW Tackling Tobacco pamphlet "*Staying a non-smoker*"
Resource 5.5	Quit kits and ordering information
Resource 6.1	Quitline Fax Referral Form
Resource 6.2	TNT Quit Plan
Resource 7.1	5A's poster
Resource 7.2	Motivational Interviewing strategies
Resource 7.3	The 5R's (for the smoker unwilling to quit)
Resource 7.4	Stage of Change approach – decision branching
Resource 7.5	Nicotine Withdrawal – What is it?
Resource7.6	NRT Use
Resource 7.7	NRT protocol
Resource 7.8	What if the NRT is not working?
Resource 7.9	Relapse and coping strategies
Resource 7.10	NRT use log (tracking sheet for NRT provided to service by TNT)
Resource 8.1	Letter to GP

8. Maintenance and Follow-Up

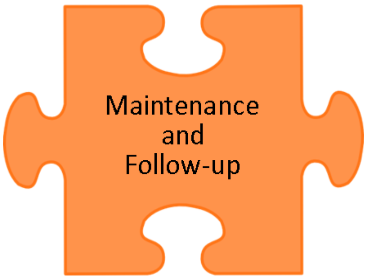

Goal: To ensure that clients have a plan in place to maintain cessation or follow-up on quit interest and intentions upon discharge from the service.

MAIN STEPS TO IMPLEMENTATION OF THIS STRATEGY

8.1. *Quit Plan*

Services will be encouraged to work through a Quit Plan (see Resource 6.2) with their clients and include a copy of this in their case notes, so that progress can be tracked over time. A copy of the quit plan should also be provided to the client upon discharge from the service, so they can refer to it in order to maintain cessation, or to help plan future quit attempts.

8.2. *Linking to Primary Healthcare Providers*

With the client's permission, a copy of their smoking information with instructions for post-discharge management will be faxed to their primary health care provider (see Resource 8.1 – Letter to GP).

8.3. *Quitline Fax Referrals*

Clients will be offered to be linked to smoking cessation services, in particular the Quitline. Services will be asked to use Quitline fax referral forms, the use of which can be tracked over the lifespan of the project (see Resource 6.1).

RESOURCES FOR THIS STRATEGY	
Resource 6.1	Quitline Fax Referral Form
Resource 6.2	TNT Quit Plan
Resource 8.1	Letter to GP

TNT Resource Kit

Resource 1.1: CCNSW Tackling Tobacco Pamphlet – "Incorporating smoking cessation into drug and alcohol treatment – Information for staff"

Available at: http://askthequestion.com.au/wp-content/uploads/2013/08/CAN10471_TT_Factsheet_Treatment_2.pdf

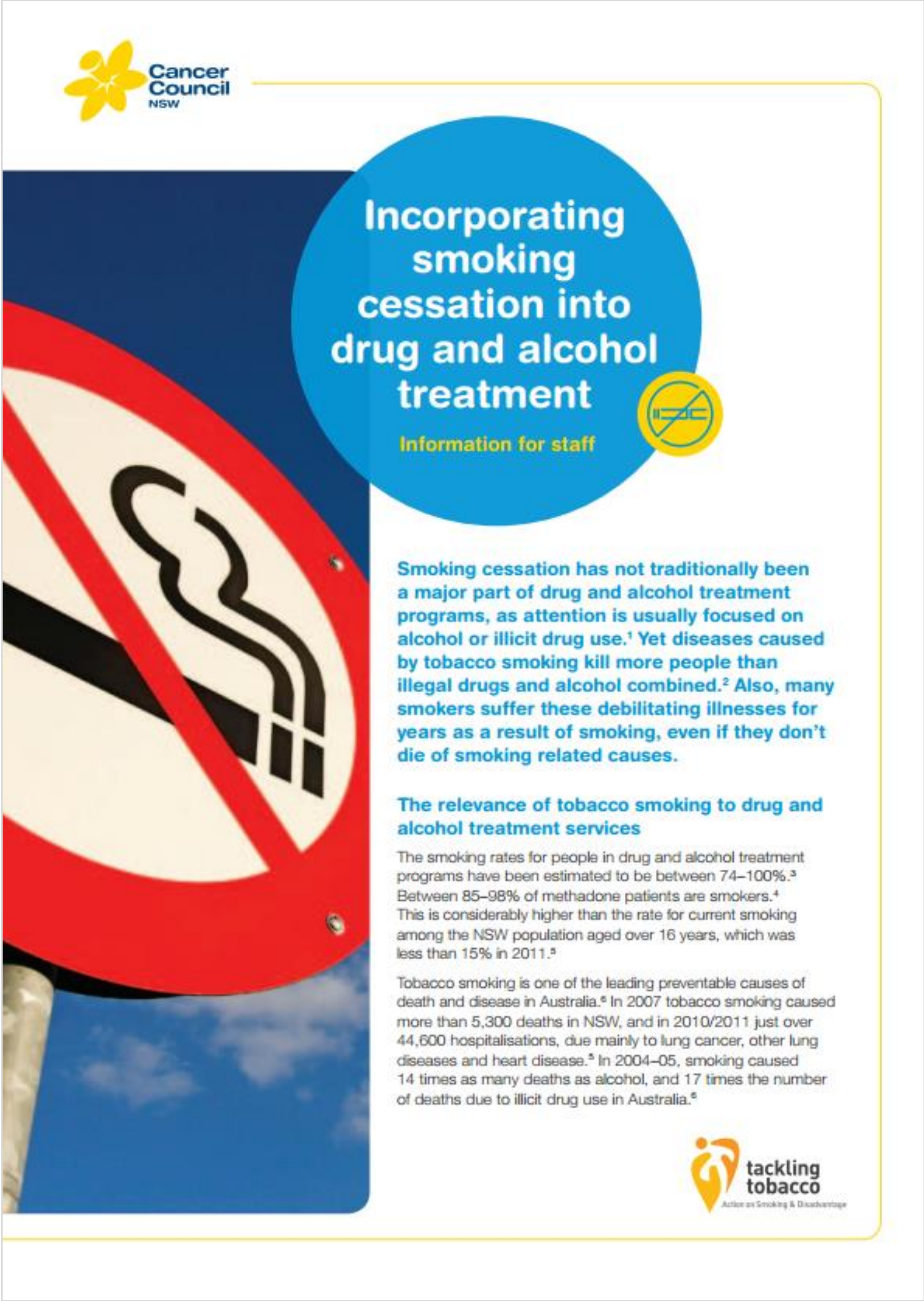

Cancer Council NSW

Incorporating smoking cessation into drug and alcohol treatment

Information for staff

Smoking cessation has not traditionally been a major part of drug and alcohol treatment programs, as attention is usually focused on alcohol or illicit drug use.[1] Yet diseases caused by tobacco smoking kill more people than illegal drugs and alcohol combined.[2] Also, many smokers suffer these debilitating illnesses for years as a result of smoking, even if they don't die of smoking related causes.

The relevance of tobacco smoking to drug and alcohol treatment services

The smoking rates for people in drug and alcohol treatment programs have been estimated to be between 74–100%.[3] Between 85–98% of methadone patients are smokers.[4] This is considerably higher than the rate for current smoking among the NSW population aged over 16 years, which was less than 15% in 2011.[5]

Tobacco smoking is one of the leading preventable causes of death and disease in Australia.[6] In 2007 tobacco smoking caused more than 5,300 deaths in NSW, and in 2010/2011 just over 44,600 hospitalisations, due mainly to lung cancer, other lung diseases and heart disease.[5] In 2004–05, smoking caused 14 times as many deaths as alcohol, and 17 times the number of deaths due to illicit drug use in Australia.[6]

tackling tobacco
Action on Smoking & Disadvantage

<u>Resource 1.2</u>: Agenda template for TNT briefing staff meeting

---

Date: dd/mm/yyyy

<u>Agenda</u>

Item No.	Agenda Item	Discussion
**1.**	**<u>Attendance/Apologies:</u>**<br>1.1. Attendance<br>1.2. Apologies	**1.1 Attendees** –<br><br>**1.2 Apologies** –
**2.**	**Action Items from the Previous Minutes**	
**3.**	**TNT project**	
**4.**	**AOB / Next Meeting**	Next meeting:

<u>Resource 1.3</u>: Newsletter article templates for TNT

The following templates have been adapted from The Cancer Council Queensland's *"Smokefree Policy Guide for Workplaces"* available for download at: http://www.health.qld.gov.au/atod/documents/smokefreepolicy_work.pdf

Article 1 – <u>Informing staff of changes to tobacco policy</u>

Going Smokefree

(name of organization) is committed to providing employees with a healthy environment which encourages high staff morale and productivity and protects the health of all employees.

With this in mind, we are proposing that *(name of organisation)* creates a smokefree policy. This policy will help to promote the health and safety of all employees and clients.

(name of organization)'s management team is setting up a smokefree policy committee which will be made up of *(insert number)* management, employee and union representatives.

The committee welcomes any suggestions or questions that you may have. Please direct these to *(insert name).*

We will keep you up-to-date with the progress of *(name of organisation)'s* new smokefree policy.

Article 2 – <u>Promote implementation of tobacco policy</u>

Ready, set, go smokefree

(Name of organisation)'s much awaited smokefree policy is ready to go!

The smokefree policy will officially commence on *(insert date)*.

The policy will see *all areas/most areas* within the *(name of organisation)*'s premises become smokefree.

A copy of the new smokefree policy will be distributed to all staff. Signage has been displayed around the premises and we appreciate your assistance and cooperation in making *(name of organisation)* a smokefree, healthy environment.

<u>Resource 1.4</u>: Reminder email or flyer prompts for changes to tobacco polices

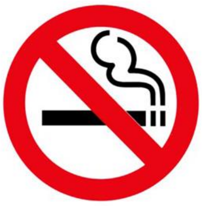

Just a reminder that

____
is now smokefree.

You can find our written smokefree policy at ________________________________

<u>Remember:</u>

- Ask every client if they smoke & record in notes
- Offer smokers help to quit (quit plan, NRT etc.)
- Arrange follow-up (next appointment, quitline etc.)

Resource 2.1: Support Champion "Identifier" Poster

Support Champion

Name has been identified as the TNT project Support Champion!

If you have any questions about the TNT project, or would like some support in implementing smoking cessation care with clients, please ask me.

<u>Resource 3.1</u>: CCNSW "Tackling Tobacco Policy Toolkit"

Available at: http://askthequestion.com.au/wp-content/uploads/2011/05/CAN-1023-Addressing-Smoking-Toolkit.pdf

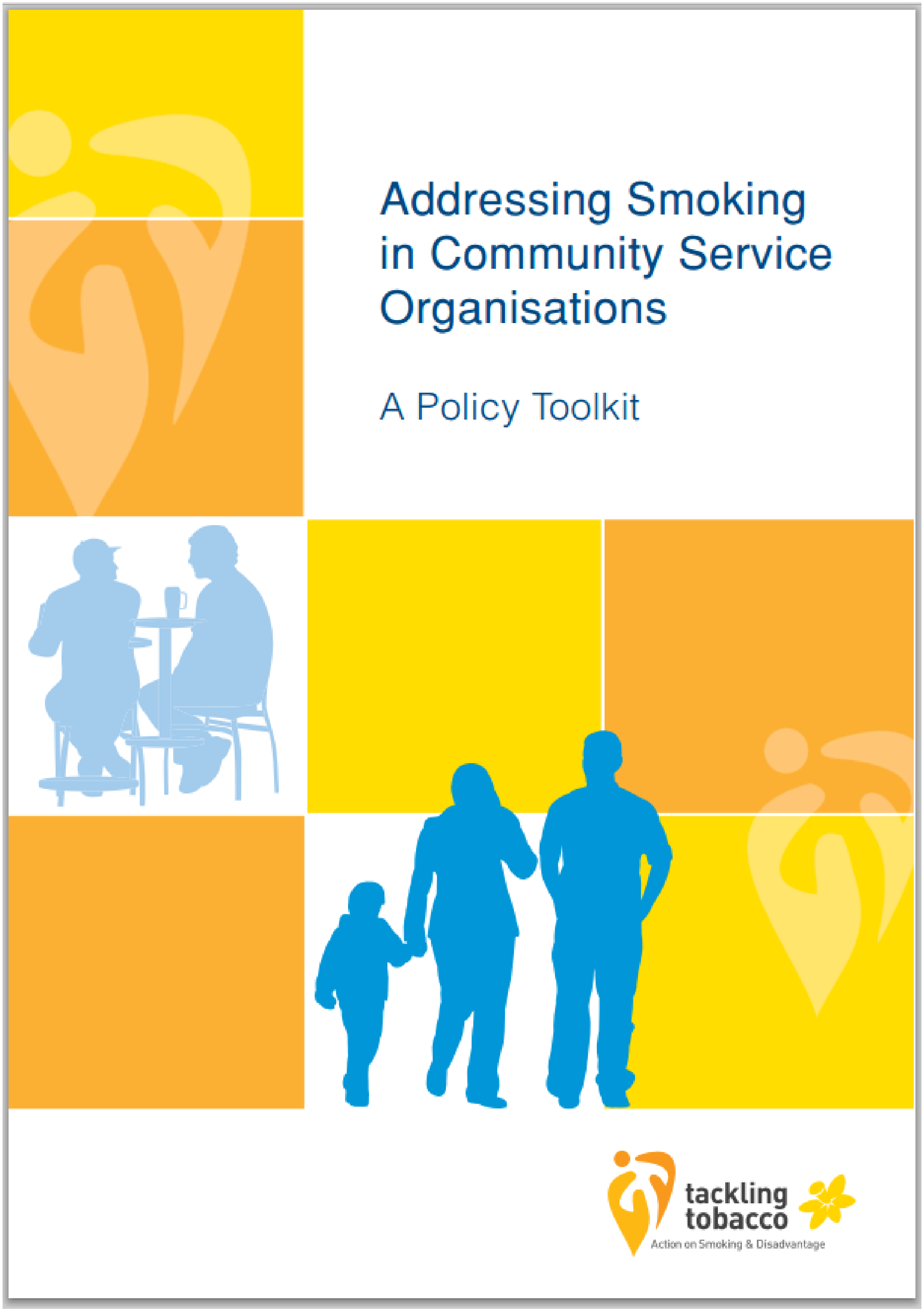

<u>Resource 4.1</u>: Desktop reminder to assess smoking status

The following desktop reminder can be folded in half to display the smoking status check reminder to service staff.

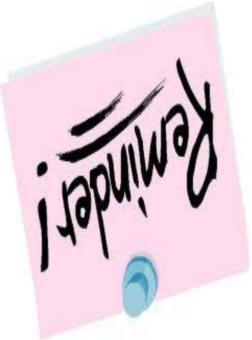

Have you asked your client about their smoking status?

Remember to record in case notes!

TNT TACKLING NICOTINE TOGETHER

Are you a smoker who is interested in quitting? Ask me how I can help.

Resource 5.1: CCNSW Tackling Tobacco pamphlet – "Not ready to give up"

Available at: http://askthequestion.com.au/wp-content/uploads/2011/05/Booklet-1.pdf

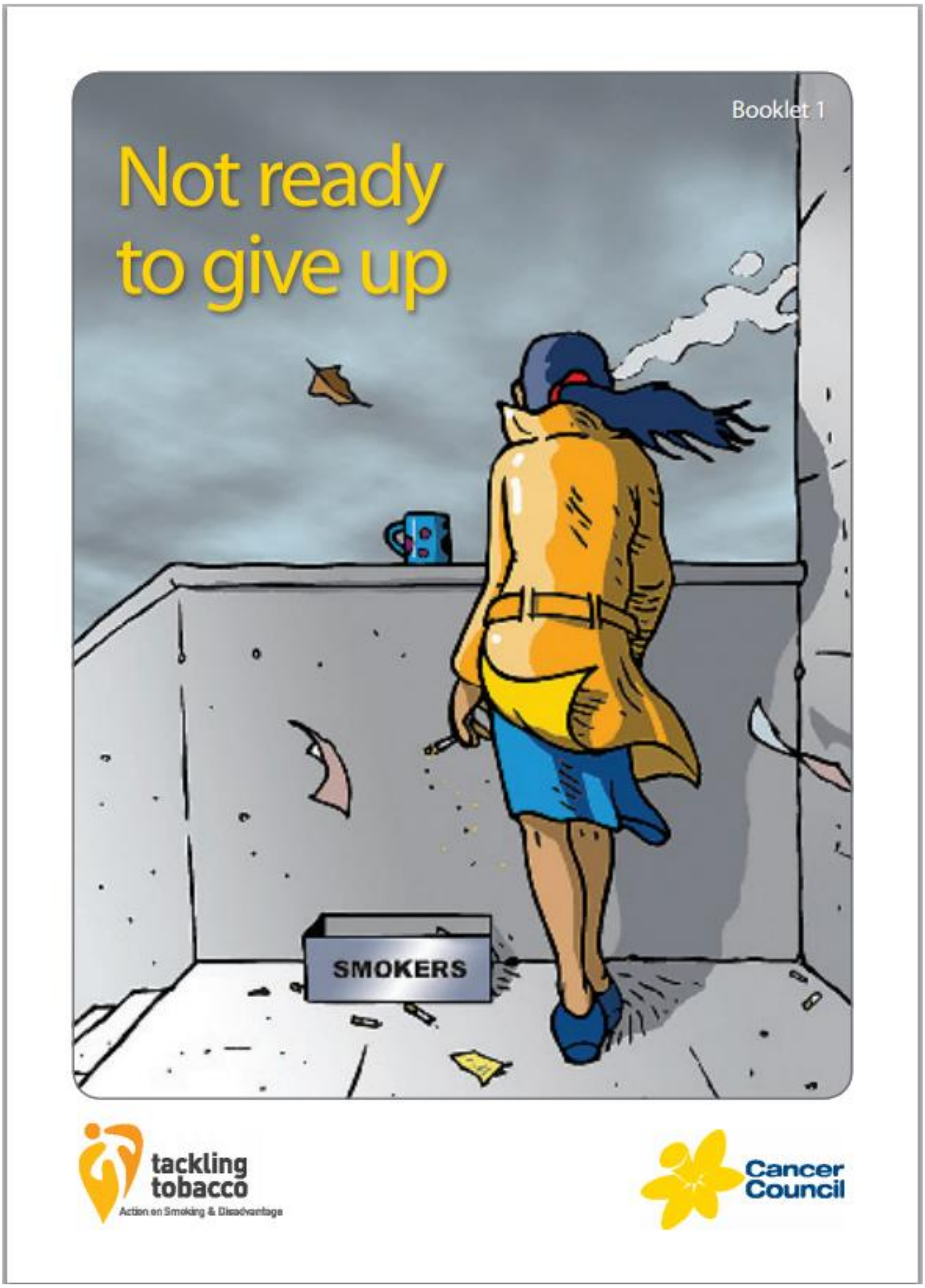

<u>Resource 5.2</u>: CCNSW Tackling Tobacco pamphlet – "Thinking about giving up"

Available at: http://askthequestion.com.au/wp-content/uploads/2011/05/Booklet-2-.pdf

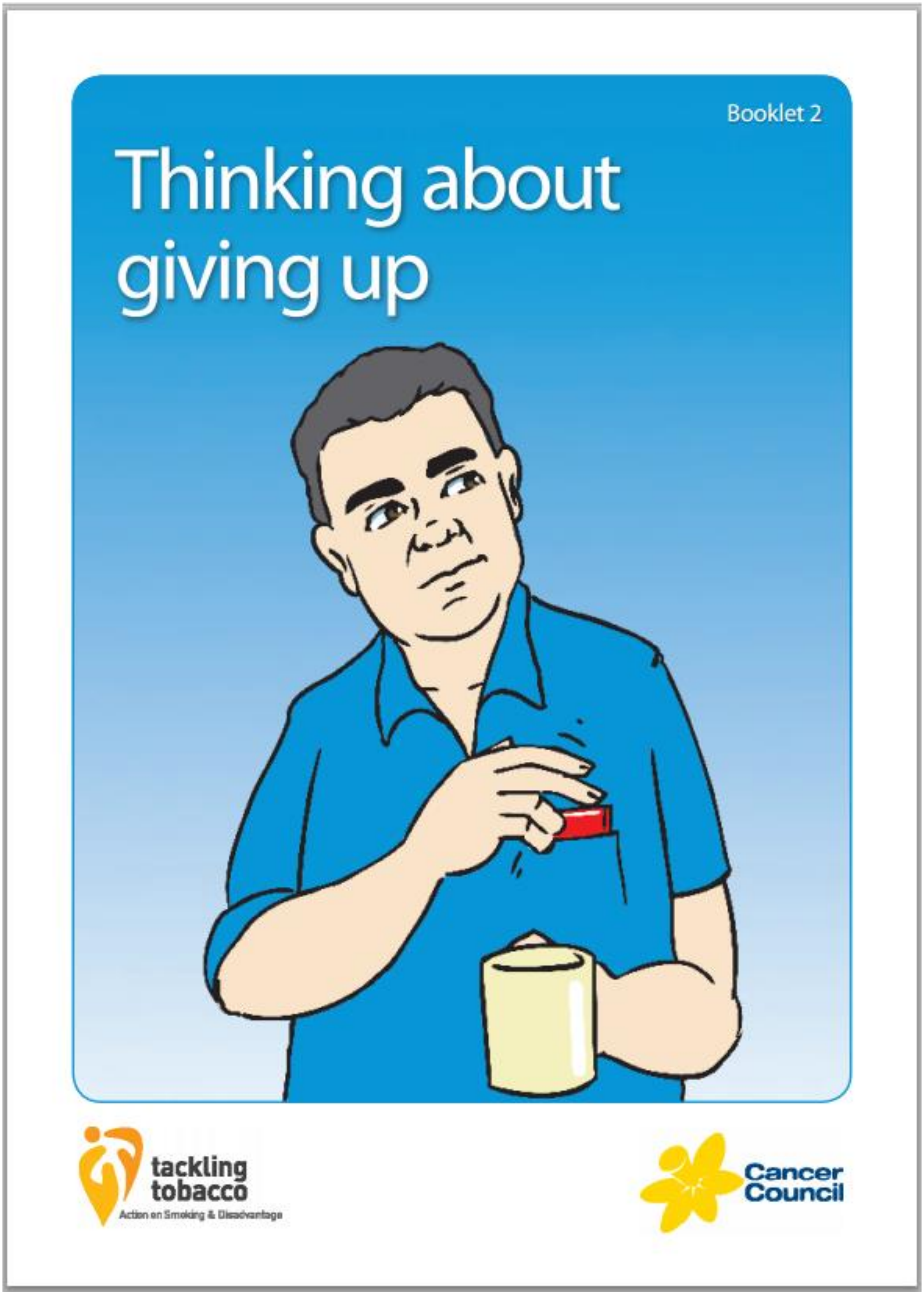

<u>Resource 5.3</u>: CCNSW Tackling Tobacco pamphlet – "Ready to give up"

Available at: http://askthequestion.com.au/wp-content/uploads/2011/05/Booklet-3.pdf

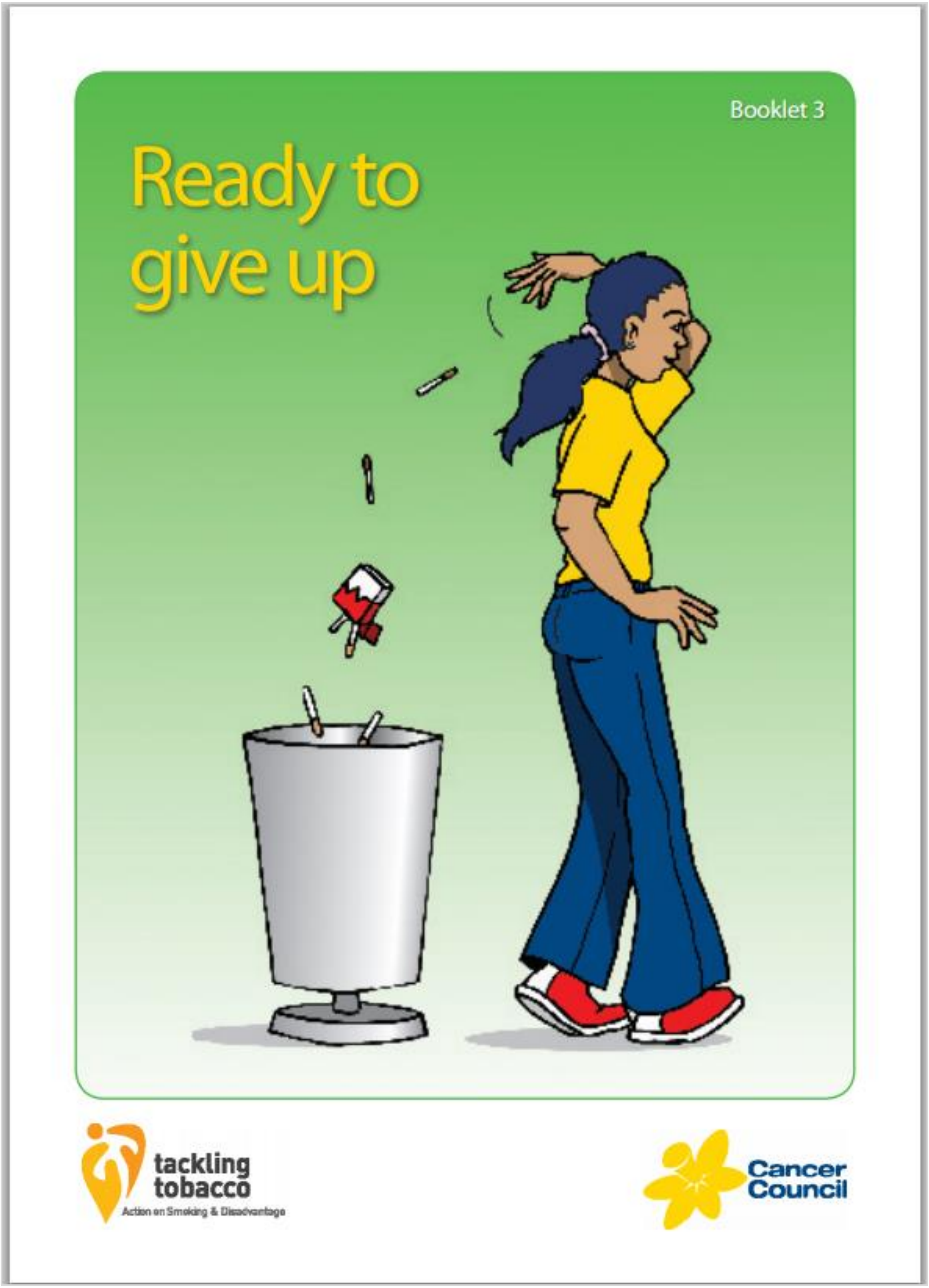

<u>Resource 5.4</u>: CCNSW Tackling Tobacco pamphlet – "Staying a non-smoker"

Available at: http://askthequestion.com.au/wp-content/uploads/2011/05/Booklet-4.pdf

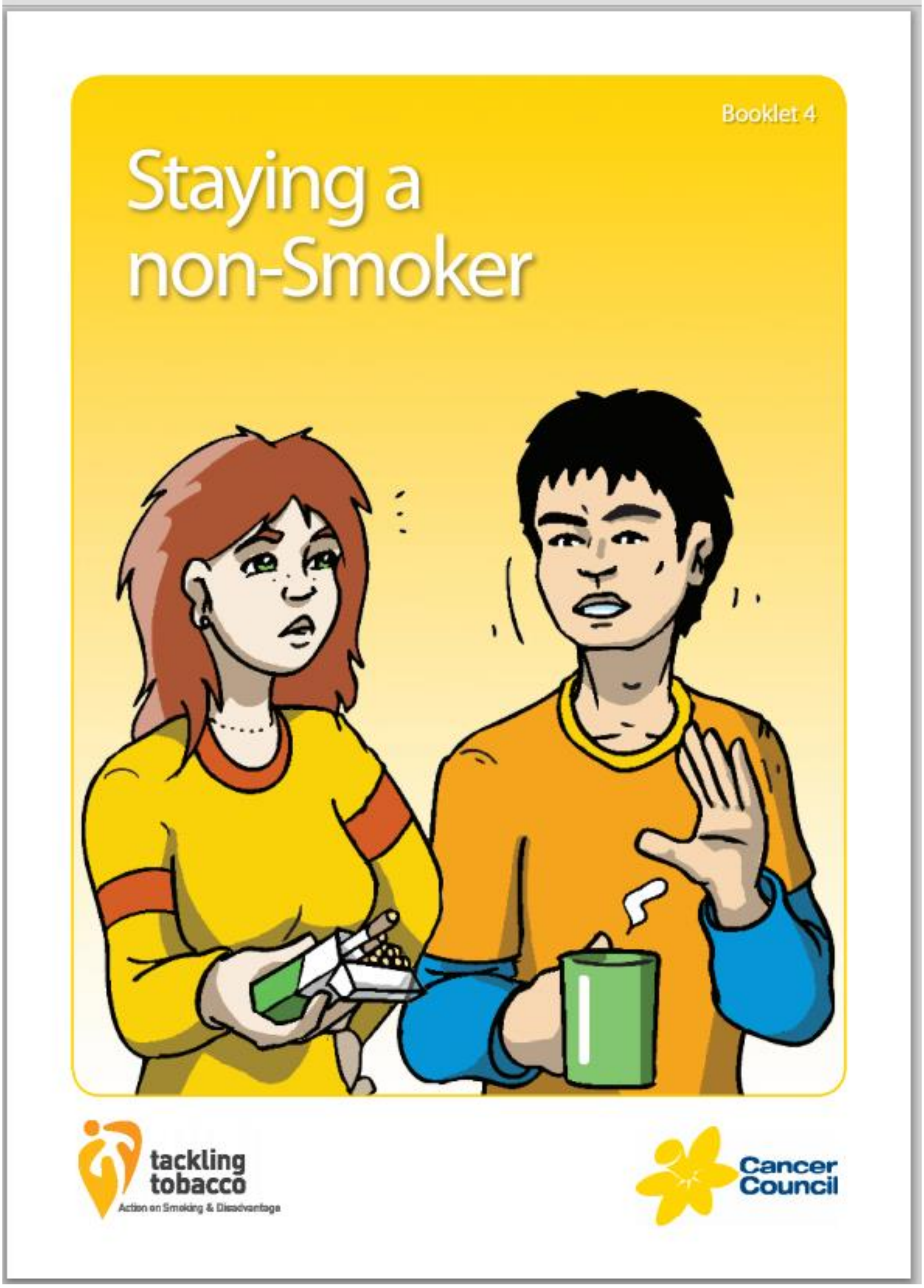

Quit Kit / Quit Pack Ordering Information

***Quit kits can be ordered from the Quitline **13 78 48**

NSW
Available online at:
http://www.icanquit.com.au/further-resources/online-quit-kit

QLD
Resource order form:
http://www.health.qld.gov.au/quitsmoking/documents/quit-orderform.pdf

ACT
Cancer Council ACT
Ph: (02) 6257 9999
E: tobaccocontrol@actcancer.org

Australian Government Campaign Resources
Request form available at:
http://www.quitnow.gov.au/internet/quitnow/publishing.nsf/Content/fact-sheets

Resource 6.1: Quitline Fax Referral Form

Available at:
http://www.quitnow.gov.au/internet/quitnow/publishing.nsf/Content/C267B0382618D7ECCA257A0D001F11DB/$File/smoking%20cessation%20form%20-%20August%202013.pdf

Smoking Cessation Referral Form

Last update August 2013

For use by health professionals to refer patients to Quitline

Fax Numbers:
ACT & NSW (02) 9361 8011 **NT** (07) 3837 5914 **Qld** (07) 3259 8217 **SA** (08) 8291 4280 **Tas** (03) 6242 8111 **Vic** (03) 9635 5520 **WA** (08) 9442 5020

Referrer Details

From: ______________________________

Address: ______________________________

Phone: ______________________________

Fax: ______________________________

Health Professional: ☐ General practitioner ☐ Dentist ☐ Pharmacist ☐ Nurse ☐ Mental health worker ☐ Aboriginal health worker

☐ Other (please specify) ....

Privacy Warning: The information contained in this fax message is intended for Quitline Staff only. If you are not the intended recipient you must not copy, distribute, take any action reliant on, or disclose any details of the information in this fax to any other person or organisation.

Patient Information – CONFIDENTIAL

Name: ______________________________ D.O.B __ __/__ __/__ __ __ __

Preferred Phone: (h) ____________ (w) ____________ (m) ____________

Email: ______________________________

Is the patient of Aboriginal or Torres Strait Islander origin?

☐ No ☐ Yes, Aboriginal ☐ Yes, Torres Strait Islander

What is the best time and day for Quitline to call?

Monday-Friday ☐ 9am-1pm ☐ 1pm-5pm ☐ 5pm-8pm

Is it okay for Quitline to leave a message?

☐ Yes ☐ No

Smoking status

☐ Daily ☐ Weekly ☐ Less than weekly Number per day ☐

What stage is your patient at with quitting?

☐ Not ready (not currently thinking of quitting) ☐ Unsure (thinking about quitting within 6 months)
☐ Ready (planning to quit within 1 month) ☐ Recent quitter (within the last year)

Use of Medication?

☐ Currently using/ planning to use Bupropion Hydrochloride (Zyban®)
☐ Currently using/ planning to use Varenicline (Champix®)
☐ Currently using/ planning to use nicotine patches/ gum/ inhaler/ lozenge/ micotab

What are the patient's health issues relevant to Quitline counsellors?

☐ Heart/lung disease ☐ Respiratory disease ☐ Diabetes ☐ Depression ☐ Anxiety
☐ Psychosis ☐ Pregnancy ☐ Other – please specify ____________

Please note
The interaction of chemicals in cigarettes and some medications e.g. Insulin, some antidepressants / antipsychotics, and the interplay between the chemicals and some symptoms can mean some smokers need monitoring of drug levels and symptoms by their GP through the quitting process.

Health Professional is monitoring the above

☐ Yes
☐ No

I consent to this information being faxed to Quitline and for Quitline Staff to call me at a time that I have suggested on this form. I understand that persons within the organisation with access to the fax machine, who may not be Quitline staff, might view this form. I understand that in Queensland my telephone calls will be recorded for the purposes of quality monitoring and service improvement.

____________________ Health Professional Signature

____________________ Patient's Signature

__ __/__ __/__ __ Date

For use by Quitline staff

Quitline Confirmation of Action on Referral Date: __ __/__ __/__ __, your referral for ____________________

has been received by Quitline on __ __/__ __/__ __ , a call back time has been organised for __ __/__ __/__ __.

Referral feedback sent back to ____________________ (referrer / GP name) on __ __/__ __/__ __.

www.quitnow.info.au

The Quitline is answered 24 hours a day. Counselling is available with hours varying dependent on State or Territory. Specialist staff will call your referred patient back at an agreed time within the next week to provide information, support and advice on smoking cessation.

Resource 6.2: TNT Quit Plan

Name: ______________________ Quit Date: ____________________

Reasons for Quitting
□ Health □ Family □ Money □ Other: ____________________________

My QUIT GOAL is: _________________________
____
____

As part of my Quit Plan I will:

□ My support person will be: ______________

□ Tell people I'm quitting

□ Ask others for support

□ Throw away lighters, cigarettes, ashtrays

□ Identify situations where I get strong cravings

□ Plan weekly rewards: __________________

□ Save the money I spend on cigarettes.

Each week I will save $____________

I will use the money to____________

Preparing to Quit

Try this activity at least once or twice during the next week:

When you feel like a smoke, try not to have one, or at least hold off for a few minutes. Observe what happens – notice your craving; how you feel; how long it takes for the craving to pass; take note of what you do to get through the craving.

Quitline

Have the following resources been set up?

□ Quitline fax referral form completed

□ Quit kit supplied

□ My QuitBuddy app signed up for

Identifying triggers and coping strategies

____
____
____
____
____

Nicotine Withdrawal Management
Please select one of the following nicotine withdrawal management plans for the patient to follow based on their Heaviness of Smoking Index score.

	Dependence	Combination Therapy	NRT Dosage
□	High<br><br>(smokes within 5mins of waking)	**Patches** 21mg (24hr)<br>AND<br>**Lozenge or Gum or Inhaler or Oral Strip**	**Patches:** 21mg/24hr x 1<br>**Lozenge:** 4mg x 6 (maximum)<br>**Gum:** 4mg x 6 (maximum)<br>**Inhaler:** up to 12 cartridges/day<br>**Oral Strip:** 2 x every 1-2 hrs
□	Moderate<br><br>(smokes within 6mins – 1hr of waking)	**Patches** 21mg (24hr)<br>AND<br>**Lozenge or Gum or Inhaler or Oral Strip**	**Patches:** 21mg/24hr x 1<br>**Lozenge:** 4mg x 6 (maximum)<br>**Gum:** 4mg x 6 (maximum)<br>**Inhaler:** up to 12 cartridges/day<br>**Oral Strip:** 1 x every 1-2 hrs
□	Low<br><br>(has first smoke an hour or more after waking)	**Patches** 21mg (24hr) or 14mg/16hr<br>AND<br>**Lozenge or Gum or Inhaler or Oral strip**	**Patches:** 1 x 21mg/24hr or 14mg/16hr<br>**Lozenge:** 2mg x up to 6/day<br>**Gum:** 2mg x 6 (maximum)<br>**Inhaler:** up to 6 cartridges/day<br>**Oral Strip:** 1 x every 1-2 hrs

Resource 6.3: CO monitor protocol

Instructions for using CO monitor

1. Confirm that participant consents to completing the CO breath analysis.

2. Explain to the participant that this machine only measures levels of carbon monoxide in the breath and cannot be used to test for other substances (for example use of alcohol or illicit substances).

3. Ask the participant if they are able to comfortably hold their breath for 15 seconds. If they are unsure, reduce the countdown timer to 10 or 5 seconds. If the participant is unable to comfortably hold their breath for 5 seconds do not continue with the CO breath analysis.

4. Using a pair of latex or rubber gloves, attach a new d-piece to the CO monitor unit.

   DO NOT carry out the CO analysis if there are no gloves available.

5. Follow the instructions per Bedfont manual. If the participant is unable to hold their breath for 15 seconds you can alter the countdown timer to go for 10 or 5 seconds. Refer to page 12 of the Bedfont manual.

6. While still wearing the gloves, remove the d-piece and place it in its individual plastic packaging and dispose of the d-piece in a general waste bin. Then dispose of the gloves in a general waste bin.

   NEVER dispose of the d-piece without wearing gloves.

7. Clean hands with Aqium gel after disposing of the d-piece.

Resource 7.1: 5A's Poster

The 5A's

Ask	• Identify and document tobacco use status for every patient at every visit.
Advise	• In a clear, strong, and personalised manner, urge every tobacco user to quit.
Assess	• Assess whether the tobacco user is willing to make a quit attempt at this time.
Assist	• Assist the patient to quit
Arrange	• Arrange follow-up contact

<u>Resource 7.2</u>: Motivational Interviewing Strategies

<u>Motivational Interviewing Strategies</u>

Strategy	How to implement it
Express empathy	Use open-ended questions to explore:<br>• The importance of addressing smoking/tobacco use (*"How important do you think it is for you to quit smoking?"*)<br>• Concerns and benefits of quitting (*"What might happen if you quit?"*)<br><br>Use reflective listening to seek shared understanding<br>• Reflect words or meaning<br>• Summarise<br><br>Normalise feelings and concerns (*"Many people worry about managing without cigarettes"*)<br><br>Support the patients autonomy and right to choose or reject change (*"I hear you saying you are not ready to quit smoking right now. I'm here to help you when you are ready."*)
Develop discrepancy	Highlight the discrepancy between the patient's present behaviour and their priorities / values / goals (*"It sounds like you are very devoted to your family. How do you think your smoking is affecting your children?"*)<br><br>Reinforce and support "change talk" and "commitment" language<br><br>Build and deepen commitment to change
Roll with resistance	Back off and use reflection when the patient expresses resistance:<br>• "*Sound like you are feeling pressured about your smoking"*<br>Express empathy:<br>• *"You are worried about how you would manage withdrawal symptoms"*<br>Ask permission to provide information:<br>• *"Would you like to hear about some strategies that can help you address that concern when you quit?"*
Support self-efficacy	Help the patient to identify and build on past successes:<br>• *"So you were fairly successful the last time you tried to quit."*<br>Offer options for achievable small steps toward change:<br>• Call the quitline for advice and information<br>• Read about quitting benefits and strategies<br>• Change smoking patterns (e.g. no smoking in home)<br>• Ask the patient to share their ideas about quitting strategies

Resource 7.3: The 5R's – for the smoker unwilling to quit

FOR THE SMOKER UNWILLING TO QUIT: "THE 5 R's"

Smokers may be unwilling to quit due to misinformation, concerned about the effects of quitting, or may be discouraged because of previous unsuccessful quit attempts. These patients may respond to brief motivational interviewing interventions (see Resource 7.2).

After asking about tobacco use, advising the smoker to quit and assessing the willingness to quit, the 5R's motivational interviewing approach should be used.

Strategy	How to Implement
**RELEVANCE**	Encourage the patient to talk about the reasons why quitting smoking is personally relevant to *them*. E.g.<br>• disease status or risk<br>• family or social situation<br>• health concerns<br>• previous quitting experience<br>• personal barriers to cessation
**RISKS**	Ask the patient to identify the negative consequences of continuing smoking – the issues that worry them the most.
**REWARDS**	Ask the patient to identify the benefits they would experience by quitting smoking. E.g.<br>• Improved health<br>• Food will taste better<br>• Improved sense of smell<br>• Saving money<br>• Feeling better about oneself<br>• Home, car, clothing, breath will smell better<br>• Setting a good example for children<br>• Having healthier babies and children<br>• Feeling better physically<br>• Improved appearance: reduced wrinkles/aging skin
**ROADBLOCKS**	Ask the patient to identify barriers to quitting. E.g.<br>• Withdrawal symptoms<br>• Fear of failure<br>• Weight gain<br>• Lack of support<br>• Depression<br>• Enjoyment of smoking<br>• Being around other tobacco users<br>• Limited knowledge of effective treatment options
**REPETITION**	Repeat this process with patients in each session, follow-up with any progress made since the last session.<br><br>Remind patient that most people make repeated quit attempts before they are successful.

Resource 7.4: Stages of Change approach – decision branching tool

STAGES OF CHANGE APPROACH

Looking at the client's stage of readiness to change may also help you in choosing an effective approach to take.

Stage of Readiness	Definition	Suggested Approach
Not ready<br>(pre-contemplation)	Not seriously thinking of quitting in the next 6 months.	**Provide the 5 R's.**<br><br>Show interest and encourage the client to think about the issues.
Unsure (contemplation)	Considering quitting in the next 6 months.	**Provide the 5 R's.**<br><br>Motivate change and offer help to identify and overcome barriers to cessation.
Ready (preparation)	Planning to quit in the next 30 days.	Provide assistance to **develop quit plan**, suggest coping strategies, 4 D's (Delay, Deep breathe, Drink Water, Do something else), encourage social support.
Action	People who have quit.	Congratulate on progress. Check for problems and if present advise or refer appropriately. Offer support and strategies to prevent relapse.
Maintenance	Smokers who've been abstinent for more than 6 months.	Congratulate and reinforce benefits of being a non-smoker.<br>Provide counselling for relapse prevention.
Relapse	Has gone back to smoking.	Reinforce that this is part of the learning experience and not a failure. Encourage and motivate to quit again.

NICOTINE WITHDRAWAL – WHAT IS IT?

When making a quit attempt, a person may experience nicotine withdrawal symptoms. It is important to be aware of these symptoms in order to understand, reassure and remind your client that these will ease (usually after the first 2-4 weeks). Emphasise that urges to smoke may continue, however they are due to stopping smoking, not the use of NRT. Here is a list of possible symptoms and tips for dealing with them:

IF THIS HAPPENS…	TRY THIS…
**Irritability, Anxiety, Tenseness**	
As the body adjusts to being without nicotine, feelings of irritability, tenseness and anxiety may be experienced.	Tip: Stress in the first 2 weeks of the quit attempt should be reduced. Ideas – short walks, deep breaths, soak in a bath, meditate.
**Difficulty Concentrating**	
Cigarette cravings may make it harder for a person to concentrate, however concentration levels will return to normal in a few weeks.	Tip: Projects can be broken up into smaller tasks, with regular breaks.
**Restlessness**	
As the body is adjusting to being without nicotine feelings of restlessness may also be experienced.	Tip: Restless energy can be used to get jobs and physical activity done. Caffeine intake should be reduced.
**Insomnia – Problems falling asleep or waking frequently**	
Some people's sleep patterns can be affected from nicotine withdrawal, including problems falling asleep, frequent waking, and strong or unusual dreams.	Tip: Relaxation exercises can be done before bed. Caffeine intake should be reduced. Avoid wearing patches while sleeping.
**Coughing, dry throat and mouth, nasal drip**	
Coughing is a sign that the tar and mucus is being removed from the lungs.	Tip: Drink plenty of water and encourage client to think of the coughing as the lungs cleaning themselves.
**Appetite Changes**	
As nicotine is an appetite suppressant, people making a quit attempt often feel hungrier.	Tip: Keep pre-prepared snacks such as sliced fruit and vegetables on hand. Glucose tablets can be taken for those who develop a sweet-tooth (Caution: people with diabetes should consult their doctor).
**Tingling Sensations and Dizziness**	
As circulation improves, some may experience tingling in fingers and toes, and dizziness.	Tip: Stay calm and sit down and rest until it passes.

NICOTINE REPLACEMENT THERAPY (NRT) USE

What is Nicotine Replacement Therapy (NRT)?

For many smokers it is the urge to smoke at the start that lead to a failed quit attempt. NRT is a way of getting nicotine into the bloodstream without smoking. NRT reduces the symptoms of nicotine withdrawal. NRT acts by providing a 'clean' alternative source of nicotine that the smoker would have otherwise received from tobacco. Nicotine delivered from NRT is absorbed more slowly and generally in a lesser amount than with cigarettes. Examples of NRT include patches, lozenges, gum, oral strips or inhalators.

Is NRT safe?

Yes NRT is safe to use. It only contains the nicotine that would otherwise have been received from cigarettes and not the other harmful constituents of tobacco smoke. It is not the nicotine that causes the health problems associated with smoking but the other things such as tar and carbon monoxide.

Clients with past experiences of NRT:

Given that NRT is now widely available, there is a good chance that smokers may have tried NRT in the past. Some will have found it unhelpful. When using these products without advice smokers may have had unrealistic expectations about how NRT works, may not have liked the initial taste and may not have used it correctly, or for long enough. It is important to encourage clients who have had past negative experiences of NRT to give it another try. You might suggest trying a different type of NRT (i.e. an inhaler instead of a patch) if a client is very hesitant to use.

Talking about NRT with clients:

"NRT is one type of medication available to help you in your quit attempt. It is effective and if used properly, will double your chances of stopping smoking – however it is not a magic cure."

"Medications are an important part of a successful quit attempt, but they are not the only part. Receiving support and advice from a counsellor like me will also roughly double your chances of stopping smoking, but you will need support from other people too. You will also need to make changes to your daily routine and will have to be highly committed to give yourself a good chance of stopping smoking for good."

What types of NRT are available provided as part of the TNT project?

Intervention sites will be provided with the following types of NRT:

Patches | Gum | Inhalator | Oral strips | Cool Drops (lozenge)

How do you use the various types of NRT?

Always inform the clients that they should read the product consumer information that comes with the NRT product.

Nicotine patches are stuck onto the skin and release nicotine into the bloodstream through the outer layer of skin into the blood vessels beneath. Each patch has a special membrane that steadily controls the release of nicotine. Each patch is designed to be worn for 24 hours. If the patch is removed before 24 hours has elapsed the wearer will not receive the full portion of the dose.

It is recommended that a patch is put on just before going to bed and at least 30 minutes after the client has smoked their last cigarette if they continue to smoke. Placing a patch on at night will assist with the cravings the following morning. Wearing a patch may cause sleep disturbances and vivid dreams. Skin irritation beneath the patch occurs in some users. To reduce the risk it is recommended that the site of the patch be changed with each new patch, and that the patch is applied to a clean, dry and hairless area.

Nicotine lozenges are dissolved in the mouth. The lozenge should be placed into the mouth and moved around intermittently. The lozenge should not be chewed, sucked or swallowed whole; just allow it to dissolve. Clients should not eat or drink while using the lozenge. Lozenges should be used whenever there is a strong urge to smoke a cigarette.

Nicotine gum is another oral NRT product**.** Each piece should be chewed slowly to release the nicotine; this will be experienced as a hot peppery taste. The gum should be 'parked' between the cheek and gums so that the nicotine can be absorbed. After a few minutes the gum can be chewed again, and then parked. This should be repeated for 20-30 minutes.

Nicotine inhalers are a cigarette shaped device that delivers nicotine through the mouth. After 20 minutes of continuous use with deep or shallow puffing all of the nicotine will have been used. It is not advised to try to make one cartridge last all day, and although every smoker is different, most successful quitters use around 6 cartridges per day.

Instructions on how to put together the inhaler:

1. Remove the mouthpiece from the plastic wrap.
2. Align the marks on the mouthpiece and pull apart.
3. Take out the blister tray.
4. Peel back to release one cartridge.
5. Press the cartridge firmly into the bottom of the mouthpiece until the seal breaks.
6. Put the top onto the mouthpiece.
7. Again align the marks on the mouthpiece and push the top and bottom firmly together to break the top seal of the cartridge.
8. Twist to misalign the marks.
9. The inhaler is now ready to use.

Nicotine oral strips are thin dissolvable translucent films that are placed on the tongue and pressed against the roof of the mouth until the strip dissolves (approx. 3 mins). At first the client should use one strip every one to two hours. The strips should not be chewed or swallowed.

How much NRT should a client use?

The amount and strength of NRT products used will differ from client to client depending on how heavy their smoking is and their personal preferences. The Heaviness of Smoking Index is a scale that measures, on average, how many cigarettes the client smokes each day and how long after waking they smoke their first cigarette. Depending on their responses clients can be classified as low, moderate or heavy smokers. The Nicotine Protocol provides suggested amounts of NRT depending on this Heaviness of Smoking Index.

DO NOT USE NRT IF:

- The client is allergic to nicotine or any of the other ingredients contained in the product pamphlet.
- The client weighs less than 45kg.

Things to consider

- If the client has Diabetes they will need to monitor their blood sugars more often as their medication requirements for insulin might alter. Encourage the client to discuss this with their General Practitioner (GP).
- Stopping smoking (with or without NRT) can alter the absorption of some medicines, and the dosage of these medicines may need to be changed. Participants should be advised of this. If the client notices an increase in side effects related to medications they should consult their GP or other health professional.
- Medications that can be affected when someone reduces or quits smoking include Warfarin, Benzodiazepines e.g. Valium, Xanax, Chlorpramazine (Largactil), Theophyllinne (used for asthma and other respiratory conditions), some beta blockers, Clozapine, Olanzapine, Haloperidol and Insulin. If the client is taking any of these medications, they should consult their doctor (before quitting smoking/using NRT).

<u>Resource 7.7</u>: NRT Protocol

**Low Dependence**	**Moderate Dependence**	**High Dependence**
1 x (14mg **OR** 21mg) patch<br>**OR**<br>Up to 6 x gum **OR** 6 x 2mg lozenge per day **OR** up to 6 inhaler cartridges per day **OR** 1 oral strip every 1 to 2 hours<br>**TIME: DAILY FOR <u>2 WEEKS</u>**	1 x 21 mg patch<br>**+**<br>Up to 6 x 4mg gum **OR** 6 x 4mg lozenge per day **OR** 6 to 12 inhaler cartridges per day **OR** 1 oral strip every 1 to 2 hours<br>**TIME: DAILY FOR <u>2 WEEKS</u>**	1 x 21 mg patch<br>**+**<br>Up to 6 x 4mg gum **OR** 6 x 4mg lozenge per day **OR** up to 12 cartridges per day **OR** 2 oral strips every 1 to 2 hours<br>**TIME: DAILY FOR <u>2 WEEKS</u>**
1 x (14mg **OR** 21mg) patch<br>+<br>***Reduce gum/lozenge/inhaler / oral strip use***<br>**TIME: DAILY FOR <u>2 WEEKS</u>**	1 x 21 mg patch (24hr)<br>+<br>***Reduce gum/lozenge/inhaler / oral strip use***<br>**TIME: DAILY FOR <u>2 WEEKS</u>**	1 x 21 mg patch (24hr)<br>+<br>***Reduce gum/lozenge/inhaler / oral strip use***<br>**TIME: DAILY FOR <u>2 WEEKS</u>**
1 x (14mg **OR** 21mg) patch<br>**TIME: DAILY FOR <u>3 – 4 WEEKS</u>**	1 x 21 mg patch<br>**TIME: DAILY FOR <u>3 – 4 WEEKS</u>**	1 x 21 mg patch<br>**TIME: DAILY FOR <u>3 – 4 WEEKS</u>**

Resource 7.8: What if the NRT is not working?

WHAT IF THE NRT ISN'T WORKING?

Client's Issue	Potential problem solvers
The patch I was wearing has given me a rash.	Always apply a new patch on a new part of the body that should be clean, hairless, and dry.
I'm having trouble sleeping/vivid dreams.	Remove the patch while sleeping.
I feel nauseous.	Consider using a different form of NRT e.g. if the client is using lozenges then try the inhaler instead.
The NRT isn't working – I'm still craving cigarettes and I'm still smoking.	Consider adding another form of NRT e.g. wearing a patch and chewing gum or having a lozenge when strong cravings occur.<br><br>Make sure the client is using the NRT product correctly – consult the product information pamphlet.
I don't like the NRT product I'm using because it doesn't taste good, it's too hard to use, I can't use it when I want to (for example chewing gum at work) or it's just not for me.	Consider using a different form of NRT e.g. if the client is using lozenges then try the inhaler instead.<br><br>Make sure the client is using the NRT product correctly – ask them to consult the product information pamphlet or go through it with them.<br><br>Talk to their GP or other health professional about other medication and strategies to quit smoking.

<u>Resource 7.9</u>: Relapse and Coping Strategies

RELAPSE AND COPING STRATEGIES

Identify high risk relapse situations, for example:

- Arguments with partners or family
- Work/financial pressures
- Christmas
- Grief
- When drinking alcohol
- On holidays
- In the company of smokers in a place where the client normally used to smoke

Pro-active strategies:

- Relapse back onto NRT instead of cigarettes – encourage patients to keep using NRT
- Develop plans to avoid and deal with the situations identified above
- Remember the reasons for quitting smoking
- Adopt active strategies, i.e. instead of a cigarette – go for a walk, read a book etc.
- Try to avoid major triggers for smoking early in the quit attempt, i.e. alcohol, coffee, smoking friends.

Coping strategies: The 4 D's

Delay	• Delay acting on the urge to smoke. After 5mins the urge to smoke weakens and resolve to quit comes back
Deep Breathing	• Take a long slow breath in and slowly release it out again. Repeat 3 times.
Drink Water	• Drink water, slowly holding it in the mouth a little longer to savour the taste.
Do Something	• Do something else to take your mind off smoking. E.g. exercise is a good alternative.

Resource 7.10: NRT log

Date	Staff Name	NRT Type (e.g. patch, inhaler)	NRT strength (e.g. 21mg/ gum 4mg)	Amount Provided (e.g. 1 box patches/14 pieces gum)

Resource 8.1: Letter to GP

Dear ___________________

This letter is to inform you that Mr / Mrs /Ms__________________________ is interested in quitting smoking.

As you are involved in this patient's physical and mental health care, we wanted to notify you or their desire to quit smoking. They would like to talk to you about their smoking and different ways to go about quitting and becoming smoke free.

We look forward to your support in helping this patient achieve their goal.

Yours sincerely,

____
